# Global trends in Alzheimer’s disease and other dementias in adults aged 55 and above (1992–2021): An age-period-cohort analysis based on the GBD 2021

**DOI:** 10.1371/journal.pone.0331204

**Published:** 2025-08-29

**Authors:** Qianqian Zhang, Yanwen Deng, Mo Xue, Zihan Ni, Guangyan Luo, Kan Tian

**Affiliations:** 1 School of Health Economics and Management, Nanjing University of Chinese Medicine, Nanjing, China; 2 School of Medicine, Nanjing University of Chinese Medicine, Nanjing, China; 3 School of Elderly Care Services and Management, Nanjing University of Chinese Medicine, Nanjing, China; Universitas Syiah Kuala, INDONESIA

## Abstract

**Background:**

Alzheimer’s disease and other dementias (ADOD) are growing global health challenges. While existing studies primarily focus on dementia prevention and management in individuals aged 65 and older, evidence suggests that cognitive decline and pathological changes begin earlier (≥55 years). This study focuses on this younger group to enable earlier risk identification and preventive interventions.

**Methods:**

This study used GBD 2021 data to extract incidence, prevalence, mortality, and DALYs related to ADOD. Trends from 1992 to 2021 were assessed using the Age-Period-Cohort (APC) model. Future burden from 2022 to 2046 was projected with the Nordpred model and validated using the Bayesian Age-Period-Cohort (BAPC) model.

**Results:**

From 1992 to 2021, ADOD incidence among individuals aged ≥55 increased by 143.88%. The age-standardized prevalence rate (ASPR) rose from 3,870.6 to 3,975.8 per 100,000. Deaths in 2021 were 1.75 times higher than in 1992. The age-standardized DALY rate was consistently higher in females, while males showed an upward trend (net drift, 0.05). APC analysis revealed the steepest incidence increase in the 60–64 age group, with earlier rises in males. Period effects indicated unfavorable incidence trends in high-middle SDI and middle-SDI regions, and similarly adverse mortality trends in high-middle and low-middle SDI regions. Projections suggest a slight increase in ASIR and ASMR by 2046, with females maintaining higher rates than males.

**Conclusion:**

The global burden of ADOD among individuals aged 55 years and above remains substantial, particularly in East Asia and among females. Given regional heterogeneity, this study recommends developing and implementing region-specific interventions for more effective improvements.

## Introduction

Dementia represents a clinical syndrome involving the progressive impairment of multiple cognitive domains. Its etiology is associated with a variety of factors, including primary neurological disorders, neuropsychiatric conditions, local medical circumstances, and potentially other contributing factors [1]. This decline affects executive, language, and visuospatial abilities, resulting in the inability to perform daily and basic activities independently [2]. Alzheimer’s disease (AD), vascular dementia (VD), Pick’s disease dementia (PiD), and dementia brought on by other particular illnesses are all included in Alzheimer’s disease and other dementias (ADOD) [3]. AD constitutes 60% to 80% of all ADOD cases, making it the most common type of dementia [4].

ADOD is currently ranked among the top ten diseases globally as a leading cause of nervous system DALYs [5]. Between 1990 and 2019, the global burden of ADOD increased substantially, with incidence and prevalence rising by 147.95% and 160.84%, respectively [6]. ADOD-induced cognitive impairment and decline in social functioning not only substantially compromise patients’ quality of life but also impose considerable burdens. The prolonged disease course, frequent comorbidities, and irreversible progression of ADOD place heavy caregiving demands on families and healthcare providers. They also generate considerable economic pressure on medical and social systems worldwide [7]. For example, in the United States, annual Medicare costs for ADOD patients aged 65 years and older were approximately threefold higher than for non-patients, with Medicaid spending differing by a factor of up to 22 [8]. ADOD-related expenditures represented 1.47% of China’s GDP, whereas globally, the share of total ADOD spending relative to global GDP was 1.09% [9]. The global number of ADOD patients is projected to surpass 130 million by 2050 [10], placing increasing strain on public health systems worldwide.

Considering the high prevalence, prolonged duration, and escalating economic burden of ADOD, early recognition and timely intervention are of paramount importance. Current research indicates that approximately 26%–42% of individuals with mild cognitive impairment (MCI) will progress to develop ADOD [11]. Furthermore, some individuals with normal cognitive function or subjective cognitive impairment test positive for β-amyloid plaques through biomarker testing and are subsequently diagnosed with preclinical AD [12]. Given the potential prevalence of MCI and preclinical AD, early identification and intervention for ADOD in this population are critical. A prospective cohort study involving approximately 10,000 participants over 30 years found that multimorbidity was linked to a 136% increase in the risk of ADOD among adults aged 55 years and older [13]. Another study confirmed the health benefits of MCI screening in individuals aged over 55 years [14]. Most existing studies focus on dementia prevention and treatment in populations aged 65 years and older. However, since cognitive decline often begins earlier, this study included individuals aged 55 years and above to explore effective intervention strategies at an earlier stage.

Based on Global Burden of Diseases (GBD) 2021, we analyzed and compared the global burden of ADOD among populations aged 55 years and older from 1992 to 2021, incorporating age-period-cohort (APC) effects and predicted morbidity and mortality trends of ADOD up to 2046 using the Nordpred package [15], validated through Bayesian age-period-cohort (BAPC) modeling. Building on prior research, this study presents several refinements. First, we leveraged the most recent GBD 2021 data to enhance the timeliness and accuracy of ADOD burden estimation. Second, given the limited prior use of the APC model, we employed this method to analyze ADOD trends, aiming to uncover disease characteristics that have remained underexplored. Third, by restricting the analysis to individuals aged 55 years and older, we sought to identify early-stage risk patterns and inform prevention strategies with epidemiological evidence.

## Materials and methods

### GBD source

The Global Burden of Disease (GBD) 2021 provided the study data, and all of the materials are openly accessible on the website (https://ghdx.healthdata.org/gbd-2021/sources). GBD 2021 is a comprehensive global database, representing the largest observational epidemiological survey, documenting the burden of 371 diseases and injuries in 204 countries and territories. Additionally, GBD 2021 complies with the Guidelines for Accurate and Transparent Health Estimates Reporting Guidelines, and earlier research has comprehensively documented its wide data sources and analysis methods [16]. Data on ADOD-related incidence, prevalence, mortality, and disability-adjusted life years (DALYs) are available in GBD 2021. The years of healthy life lost as a result of sickness are known as DALYs, which are the total of years of life lost (YLLs) and years lived with disability (YLDs). In the current study, countries were classified into 21 distinct regions based on their geographical locations for analysis purposes. The socio-demographic index (SDI) is a composite metric positively correlated with per capita income and average years of schooling and negatively correlated with the total fertility rate among women under the age of 25 [17]. Based on SDI, GBD 2021 divides 204 nations and regions into five groups: high-SDI, high-middle SDI, middle-SDI, low-middle SDI, and low-SDI regions.

In this study, ADOD was defined as a chronic, progressive, and degenerative neurological disorder characterized by memory impairment and neurological dysfunction. This definition follows the International Classification of Diseases, 10th edition (ICD-10), and the GBD 2021. ADOD was classified according to the ICD-10 coding system, encompassing codes F00 to F02.0, F02.8 to F03.91, F06.2, G30 to G31.1, and G31.8 to G32.89, which include AD, VD, PiD, and other cause-specific dementias [18]. All estimates were reported with 95% uncertainty intervals (UIs), derived from 1,000 repeated samplings, with bounds corresponding to the 2.5th and 97.5th percentiles of the uncertainty distribution [19]. In this study, data on ADOD, including incidence, prevalence, mortality, and DALYs, as well as their corresponding rates, were extracted from the GBD 2021 database, covering the period from 1992 to 2021 at national, regional, and global levels. The data are categorized by gender (male, female, both), nine age groups (55–59, 60–64, 65–69, 70–74, 75–79, 80–84, 85–89, 90–94 and 95 + years), and year (1992–2021).

### Statistical analysis

The global burden of ADOD in individuals aged 55 and above is analyzed descriptively in this study. To compare incidence, prevalence, mortality, and DALYs across genders, countries, and regions, we used age-standardized rates (ASRs) to adjust for differences in population age structures. These indicators are calculated per 100,000 individuals, with standardized values according to the GBD standard population [20]. The direct method was applied to calculate the ASRs, where age-specific rates (a_i_) for each age group were multiplied by the corresponding weights (w_i_) of individuals in the standard population. The weighted products were subsequently aggregated, normalized by the total weights of the standard population, and then multiplied by 100,000 to obtain the ASR, calculated as follows:


$$ASR=\frac{\sum_{i=1}^{A}a_{i}w_{i}}{\sum_{i=1}^{A}w_{i}}\times 100,000.$$

This study utilized the age-period-cohort (APC) model for statistical analysis. APC is a statistical model used to analyze disease-related information [21], separating research indicators into three components: age, period, and cohort, and examining the effects by isolating these three factors. The APC model can estimate overall temporal trends, incidence, and mortality trends across different age groups. It can also hypothesize the impact of changes in lifestyle habits and healthcare levels on disease incidence and mortality. Local drift refers to the APC effect specific to particular age groups or cohorts, quantified as the APC (% per year) for each group. Net drift represents the overall APC trend in the population, expressed as the annual percentage change (% per year). Even minor changes in the drift coefficients can affect the fitted rates for the period 1992–2021. This study utilized net drift and local drift to assess the ASR trends of ADOD from 1992 to 2021. Drift coefficients and their corresponding 95% confidence intervals (CIs) were calculated. The study analyzed the data from the APC model through the freely available APC web tool (https://analysistools.cancer.gov/apc/#) [22]. The introduction of the APC web tool and its approach to resolving the identifiability challenge of the APC model are comprehensively detailed in the supplementary materials. The study categorized the incidence and mortality rates of ADOD into 5-year age groups, starting from 55–59 and continuing up to 95 + years, and divided the years 1992–2021 into successive 5-year periods (1992–1996 to 2017–2021). Because the APC model requires equal intervals for both age and period, specific years (1992, 1997, …, 2017) were used instead of 5-year periods, ensuring no overlap in time between consecutive birth cohorts from 1992 to 2021. Consequently, this study included 14 partially overlapping cohorts, each covering a 10-year birth range from 1892–1901 to 1957–1966. In the APC model, the age effect is captured by age-specific rates, while the period and cohort effects are evaluated by comparing the age-specific rates of each period or cohort to a reference, thereby identifying the relative risk (RR) of incidence and mortality. RR is employed to evaluate the effects of age, period, and cohort [23]. For explanations of RR, please refer to the supplementary materials. The Wald χ² test was utilized to examine the statistical significance of the annual percentage change trend, where **p* *< 0.05 was considered significant. All statistical analyses were conducted using two-tailed tests. The reference period and cohort were selected at random and did not affect the interpretation of the results [24].

To forecast the future incidence and mortality rates of ADOD, we employed the Nordpred model to analyze the number of incident cases, deaths, incidence rates, and mortality rates of ADOD across different age groups from 2022 to 2046. Additionally, ASRs were calculated separately by gender [25]. To validate the reliability of the projection results, we employed the BAPC model for verification. For detailed methodological descriptions of the Nordpred and BAPC models, refer to the supplementary materials. All statistical analyses were performed using R software (version 4.4.1) and Excel, with a significance level set at **p* *< 0.05.

### Ethics statement

This research did not require permission from an Ethics committee because it utilized solely data from publicly accessible secondary databases.

### Patient and public involvement statement

It was neither suitable nor feasible to include patients or the general public in the research’s conception, implementation, reporting, or dissemination plans.

## Results

### Descriptive analysis of results

#### Trends in incidence rate of ADOD.

From 1992 to 2021, the number of new ADOD cases worldwide among individuals aged 55 and older rose from 3,856,613.86 to 9,405,626.27, with the incidence of ADOD increasing by 143.88%. The age-standardized incidence rate (ASIR) slightly increased from 667.22 per 100,000 (95% UI, 465.83 to 898.17) to 679.44 per 100,000 (95% UI, 466.33 to 922.85). However, the net drift was 0 (95% CI, −0.02 to 0.01), suggesting that the overall ASIR remained stable during this period. Females demonstrated a greater susceptibility to ADOD compared to males, as reflected by the consistent sex ratio of 1.28:1 in both 1992 and 2021. However, from 1992 to 2021, the net drift for males was 0.03 (95% CI, 0.01 to 0.06), suggesting a slight upward trend in ASIR among males. (Table 1)

**Table 1:** Global, sex, and SDI trends in the incidence of Alzheimer’s disease and other dementias among adults aged 55 years and older, from 1992 to 2021.

Characteristics	1992		2021		1992–2021
	Incident cases, n (95% UI)	ASIR per 100 000, n (95% UI)	Incident cases, n (95% UI)	ASIR per 100 000, n (95% UI)	Net Drift (%/year)
Global	3856613.86 (2680994.62 to 5201269.7)	667.22 (465.83 to 898.17)	9405626.27 (6449244.73 to 12788018.87)	679.44 (466.33 to 922.85)	0 (−0.02 to 0.01)
Sex					
Male	1336053.52 (916524.03 to 1819707.32)	571.41 (394.48 to 775.69)	3447541 (2344534.62 to 4715262.01)	585.06 (398.91 to 798.96)	0.03 (0.01 to 0.06)
Female	2520560.33 (1763882.83 to 3384134.85)	730.59 (512.24 to 980.66)	5958085.27 (4106756.27 to 8087478.4)	751.18 (517.67 to 1019.3)	0.01 (0 to 0.03)
SDI					
High SDI	1448931.49 (1032724.18 to 1915647.28)	720.69 (513.17 to 954.92)	2880559.56 (2013630.61 to 3871095.21)	695.38 (485.15 to 935.17)	−0.04 (−0.06 to −0.01)
High-middle SDI	1002315.73 (689904.02 to 1361906.26)	677.95 (467.98 to 920.24)	2483945.08 (1697255.99 to 3389685.86)	751.7 (513.68 to 1025.46)	0.22 (0.18 to 0.26)
Middle SDI	857671.02 (582973.33 to 1174142.94)	654.3 (446.55 to 893.28)	2744827.26 (1870380.44 to 3766015.17)	701.84 (479.57 to 961.29)	0.07 (0.05 to 0.1)
Low-middle SDI	405028.45 (275250.34 to 554557.47)	538.67 (367.39 to 735.65)	989968.19 (671273.61 to 1356015.02)	523.25 (355.73 to 715.32)	−0.16 (−0.17 to −0.14)
Low SDI	138190.61 (93883.04 to 189775.48)	534.5 (364.81 to 731.06)	298154.99 (202194.73 to 407467.66)	512.69 (349 to 698.09)	−0.2 (−0.23 to −0.17)

SDI, socio-demographic index; ASIR, age-standardized incidence rate; 95% UI, 95% Uncertainty Interval; 95% CI, 95% Confidence Interval.

Between 1992 and 2021, the global ASIR of ADOD increased slightly from 667.22 per 100,000 (95% UI, 465.83 to 898.17) to 679.44 per 100,000 (95% UI, 466.33 to 922.85). Females consistently demonstrated greater susceptibility than males, with a stable sex ratio of 1.28:1 in both years. At the SDI regional level, the ASIR increased in high-middle SDI and middle-SDI regions in 2021 compared to 1992, in contrast to decreases observed in high-SDI, low-middle SDI, and low-SDI regions. (Interpretation of Table 1)

Among the five SDI regions, the ASIR of ADOD increased in high-middle SDI and middle-SDI regions by 2021, while it decreased in high-SDI, low-middle SDI, and low-SDI regions compared to 1992. From 1992 to 2021, the ASIR in the high-SDI regions consistently remained the most elevated over the long term, whereas the ASIR in the high-middle SDI regions exhibited a continuous upward trend. By 2014, the high-middle SDI regions surpassed the high-SDI regions, becoming the region with the highest incidence rate. The high-middle SDI regions demonstrated the most substantial increase among all regions, with a net drift of 0.22 (95% CI, 0.18 to 0.26). The ASIR rose from 677.95 per 100,000 (95% UI, 467.98 to 920.24) in 1992 to 751.7 per 100,000 (95% UI, 513.68 to 1025.46) in 2021. Among the regions with a decline in ASIR, the low-SDI regions experienced the steepest reduction, with a net drift of −0.2 (95% CI, −0.23 to −0.17). The ASIR declined from 534.5 per 100,000 (95% UI, 364.81 to 731.06) in 1992 to 512.69 per 100,000 (95% UI, 349 to 698.09) in 2021. (Table 1, S1 Fig in S1 File)

**Fig 1:**
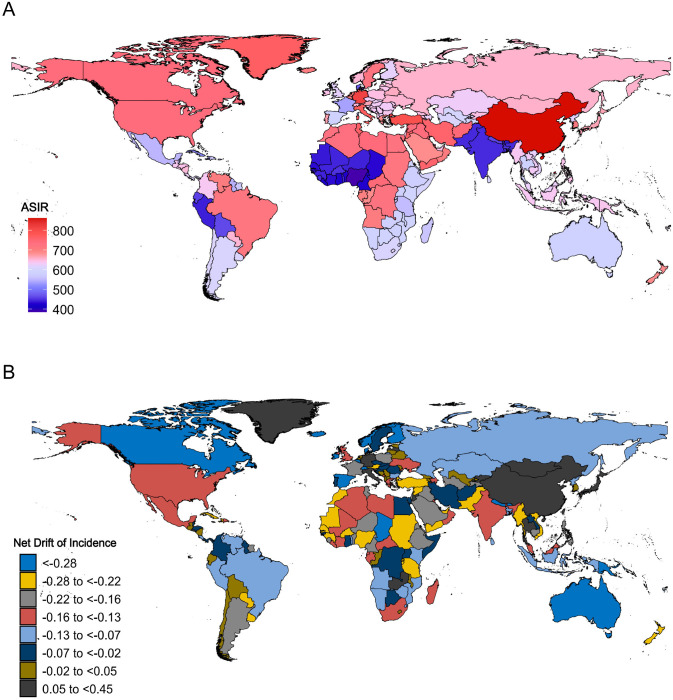
Incidence of Alzheimer’s disease and other dementias among adults aged 55 years and older across 204 countries and territories from 1992 to 2021. A: World map of ASIR for Alzheimer’s disease and other dementias in 2021; B: World map of net drifts for Alzheimer’s disease and other dementias incidence (i.e., estimated annual percentage change in incidence from the APC model). Net drift captures components of the trends attributable to calendar time and successive birth cohorts. ASIR, age-standardized incidence rate; APC, age-period-cohort. At the country level, Nigeria and Sao Tome and Principe recorded the lowest ASIR of Alzheimer’s disease and other dementias among adults aged 55 years and older in 2021, whereas China and Germany reported the highest ASIR. Between 1992 and 2021, geographic heterogeneity characterized global Alzheimer’s disease and other dementias ASIR patterns, manifested as rising trends in select countries and territories while a majority demonstrated declining trajectories. (Interpretation of Fig 1).

The analysis revealed a statistically significant positive correlation between ASIR and SDI levels (ρ = 0.38, **p* *< 0.001). (S2 Fig in S1 File) At the regional level, East Asia consistently exhibited high ASIR, rising to the highest and fastest-growing ASIR among the 21 regions by 2021 (net drift, 0.37, 95% CI, 0.32 to 0.43). (S1 Table in S1 File) North Africa and Middle East, High-income North America, and Tropical Latin America followed in ASIR. Unlike East Asia and High-income Asia Pacific, where ASIR increased rapidly, these regions showed a declining trend. (S3 Fig in S1 File) At the country level, Tokelau and Niue reported the lowest number of cases in 2021, while China and the United States had the highest. Nigeria and Sao Tome and Principe recorded the lowest ASIR, whereas China and Germany had the highest ASIR. Overall, Micronesia (Federated States of), Maldives, and China exhibited the most significant increases in ASIR. Across the 1992–2021 period, many countries exhibited declining ASIR trends, with Denmark, Norway, and Canada showing the most pronounced decreases. (S2 Table in S1 File, Figs 1A and 1B)

### Trends in prevalence rate of ADOD

By 2021, the global prevalence of ADOD reached 54,905,286.05 cases, representing 1.47 times the number of cases in 1992. Over the same period, the age-standardized prevalence rate (ASPR) rose slightly from 3,870.6 per 100,000 (95% UI, 3080.04 to 4799.91) to 3,975.78 per 100,000 (95% UI, 3131.76 to 4965.09), exhibiting a net drift of 0.01 (95% CI, 0 to 0.02), indicating a relatively stable trend. The ASPR for females was consistently about 1.3 times that for males in both 1992 and 2021. While males had a lower prevalence of ADOD, their ASPR showed a modestly higher growth trend compared to females over 1992–2021 (net drift, 0.04, 95% CI, 0.02 to 0.05). (Table 2)

**Table 2:** Global, sex, and SDI trends in the prevalence of Alzheimer’s disease and other dementias among adults aged 55 years and older, from 1992 to 2021.

Characteristics	1992		2021		1992–2021
	Prevalence cases, n (95% UI)	ASPR per 100 000, n (95% UI)	Prevalence cases, n (95% UI)	ASPR per 100 000, n (95% UI)	Net Drift (%/year)
Global	22219463.89 (17659952.46 to 27594696.06)	3870.6 (3080.04 to 4799.91)	54905286.05 (43228738.62 to 68594042.73)	3975.78 (3131.76 to 4965.09)	0.01 (0 to 0.02)
Sex					
Male	7688456.83 (6050374.27 to 9613280.24)	3273.84 (2581.81 to 4087.21)	19855926.85 (15474985.23 to 24933535.22)	3367.78 (2626.54 to 4227.63)	0.04 (0.02 to 0.05)
Female	14531007.06 (11592656.12 to 17989874.75)	4247.11 (3389.18 to 5254.13)	35049359.2 (27727089.28 to 43712875.64)	4414.52 (3492.78 to 5504.77)	0.03 (0.02 to 0.04)
SDI					
High SDI	8240297.5 (6643299.65 to 10110877.68)	4135.57 (3331.34 to 5076.53)	16871817.32 (13408805.74 to 20903119.14)	4056.36 (3222.64 to 5026.88)	0 (−0.03 to 0.03)
High-middle SDI	5815572.9 (4590172.26 to 7252114.57)	3961.89 (3130.01 to 4936.95)	14480533.5 (11331175.76 to 18179790.92)	4393.63 (3439 to 5514.2)	0.21 (0.17 to 0.24)
Middle SDI	4998893.34 (3924576.62 to 6271364.49)	3811.7 (2992.43 to 4778.82)	16101011.37 (12596761.11 to 20247236.97)	4145.89 (3245.57 to 5208.98)	0.09 (0.06 to 0.12)
Low-middle SDI	2337611.61 (1834183.61 to 2936370.88)	3089.31 (2424.44 to 3877.49)	5683826.73 (4457243.19 to 7148543.95)	2992.19 (2345.49 to 3763.73)	−0.17 (−0.17 to −0.16)
Low SDI	801287.88 (627826.64 to 1004440.26)	3065.66 (2400.08 to 3840.6)	1720808.15 (1351030.99 to 2157652.95)	2930.09 (2300.71 to 3672.62)	−0.21 (−0.23 to −0.18)

SDI, socio-demographic index; ASPR, age-standardized prevalence rate; 95% UI, 95% Uncertainty Interval; 95% CI, 95% Confidence Interval.

In 2021, the global prevalence of Alzheimer’s disease and other dementias reached 54,905,286.05 cases, which was 1.47 times the 1992 case count. The ASPR in females was 1.3 times higher than in males, a consistent disparity observed in both 1992 and 2021. Geographically, only high-middle SDI and middle-SDI regions exhibited significant increases in ASPR over this three-decade period. (Interpretation of Table 2)

From 1992 to 2021, an increasing trend in ASPR was noted exclusively in the high-middle SDI and middle-SDI regions (high-middle SDI net drift: 0.21, 95% CI, 0.17 to 0.24; middle-SDI net drift: 0.09, 95% CI, 0.06 to 0.12). In the high-middle SDI regions, ADOD prevalence increased from 5,815,572.9 cases in 1992 to 14,480,533.5 cases in 2021, with the highest ASPR recorded at 4,393.63 per 100,000 (95% UI, 3,439–5,514.2). (Table 2)

A positive correlation was observed between SDI levels and ASPR across 21 regions (ρ = 0.37, **p* *< 0.001). (S4 Fig in S1 File) Prevalence rates decreased in all areas between 1992 and 2021, with the exception of East Asia and High-income Asia Pacific. East Asia exhibited the fastest ASPR increase (net drift, 0.37, 95% CI, 0.34 to 0.41), climbing from fifth to the highest ASPR among the 21 regions (5106.19 per 100,000, 95% UI, 3979.84 to 6412.27). Conversely, Australasia recorded the largest ASPR decline (net drift, −0.49, 95% CI, −0.53 to −0.44). (S3 Table in S1 File, S5 Fig in S1 File)

**Fig 2:**
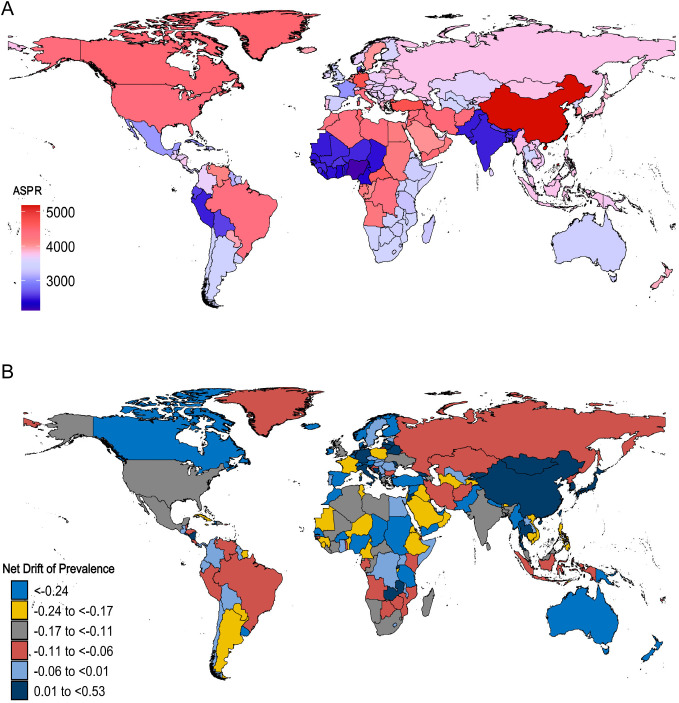
Prevalence of Alzheimer’s disease and other dementias among adults aged 55 years and older across 204 countries and territories from 1992 to 2021. A: World map of ASPR for Alzheimer’s disease and other dementias in 2021; B: World map of net drifts for the prevalence of Alzheimer’s disease and other dementias (i.e., estimated annual percentage change in prevalence from the APC model).Net drift captures components of the trends attributable to calendar time and successive birth cohorts. ASPR, age-standardized prevalence rate; APC, age-period-cohort. In 2021, China exhibited the highest ASPR globally. While most countries exhibited a declining trend in ASPR from 1992 to 2021, China exhibited a distinct pattern, with the most marked increase in prevalence. (Interpretation of Fig 2).

In 2021, China had the highest ASPR, while Canada, which ranked first in ASPR in 1992, became one of the countries with the fastest ASPR decline, second only to Denmark and Norway. Over the past 30 years, most countries experienced a downward trend in ASPR, although a few demonstrated an increase. The country with the largest upward trend in prevalence was China (net drift, 0.38, 95% CI, 0.34 to 0.41), with Japan coming in second (net drift, 0.34, 95% CI, 0.26 to 0.41). (S4 Table in S1 File, Figs 2A and 2B)

### Trends in mortality rate of ADOD

The global number of deaths from ADOD increased to 1,943,723.27 in 2021, reflecting a 1.75-fold increase compared to 1992. While deaths increased, the age-standardized mortality rate (ASMR) remained relatively stable from 1992 to 2021 (net drift, 0, 95% CI, −0.01 to 0.01). Female ADOD patients consistently had higher mortality than male ADOD patients, with ASMR ratios of 1.37:1 in 1992 and 1.35:1 in 2021. During the study period, ASMR trends for both genders exhibited a similar upward trajectory. However, the increase was more pronounced in males (net drift, 0.07, 95% CI, 0.05 to 0.09). (Table 3) In the five SDI regions, the ASMR in the high-SDI regions demonstrated a downward trend (net drift, −0.07, 95% CI, −0.09 to −0.04), decreasing from 159.42 per 100,000 (95% UI, 39.5 to 423.11) in 1992 to 154.51 per 100,000 (95% UI, 39.91 to 393.7) in 2021. The upward trend in ASMR was most prominent in low-middle SDI and low-SDI regions, particularly in the low-middle SDI regions (net drift, 0.39, 95% CI, 0.36 to 0.43). However, the low-middle SDI regions had the lowest ASMR in both 1992 and 2021. Notably, ASMR in the low-SDI regions consistently exceeded that in the low-middle SDI regions, with the trend being especially pronounced among females. (Table 3, S6 Fig in S1 File)

**Table 3:** Global, sex, and SDI trends in the mortality of Alzheimer’s disease and other dementias among adults aged 55 years and older, from 1992 to 2021.

Characteristics	1992		2021		1992–2021
	Death cases, n (95% UI)	ASMR per 100 000, n (95% UI)	Death cases, n (95% UI)	ASMR per 100 000, n (95% UI)	Net Drift (%/year)
Global	707375.35 (170043.68 to 1959602.03)	147.57 (35.66 to 402.45)	1943723.27 (484157.15 to 5169896.02)	148.24 (37 to 392.52)	0 (−0.01 to 0.01)
Sex					
Male	212663.2 (49450.94 to 607770.61)	118.68 (27.68 to 333.47)	622675.85 (147603.8 to 1759059.42)	121.94 (28.99 to 340.71)	0.07 (0.05 to 0.09)
Female	494712.15 (120666.56 to 1347844.4)	162.8 (39.83 to 438.84)	1321047.42 (336176.84 to 3415771.88)	164.29 (41.79 to 425.37)	0.01 (0 to 0.03)
SDI					
High SDI	300099.7 (74295.19 to 800754.41)	159.42 (39.5 to 423.11)	717978.89 (186248.75 to 1812752.85)	154.51 (39.91 to 393.7)	−0.07 (−0.09 to −0.04)
High-middle SDI	181523.14 (43418.13 to 508584.31)	152.61 (36.58 to 421.4)	488133.01 (119543.42 to 1324387.31)	155.71 (38.15 to 420.95)	0 (−0.02 to 0.03)
Middle SDI	140604.87 (33077.66 to 400081.8)	139.93 (33 to 390.46)	490156.43 (118840.27 to 1339405.74)	144.69 (35.22 to 390.96)	−0.04 (−0.06 to −0.01)
Low-middle SDI	62607.42 (14398.64 to 180795.45)	105.03 (24.29 to 298.4)	187922.62 (44170.65 to 528620.28)	117.77 (27.85 to 327.01)	0.39 (0.36 to 0.43)
Low SDI	21809.89 (4930.35 to 63529.7)	115.12 (26.11 to 329.09)	57943.17 (13319.93 to 165598)	129.99 (30.16 to 364.6)	0.37 (0.32 to 0.43)

SDI, socio-demographic index; ASMR, age-standardized mortality rate; 95% UI, 95% Uncertainty Interval; 95% CI, 95% Confidence Interval.

During the study period, ASMR trends showed a consistent increase in both sexes, though the rise was significantly steeper in males (net drift, 0.07, 95% CI, 0.05 to 0.09). Across SDI regions, ASMR trends diverged markedly, with high-SDI regions exhibiting a downward trend (net drift, −0.07, 95% CI, −0.09 to −0.04). In contrast, the most pronounced upward trends were observed in low-middle and low-SDI regions. (Interpretation of Table 3)

ASMR showed a positive correlation with SDI across the 21 regions (ρ = 0.32, *p* < 0.001). (S7 Fig in S1 File) In 2021, East Asia had the highest number of ADOD deaths among individuals aged 55 and older, amounting to 504,860.84 cases. In South Asia, Central Sub-Saharan Africa, Eastern Sub-Saharan Africa, Western Sub-Saharan Africa, Southeast Asia, and Southern Sub-Saharan Africa, ASMR demonstrated an increasing pattern over time. In 1992, South Asia had the lowest ASMR (84.16 per 100,000; 95% UI, 18.58 to 244.32). However, its rate of increase over the course of the study (net drift, 0.61, 95% CI, 0.57 to 0.65) significantly exceeded that of other regions. Between 1992 and 2021, ASMR remained consistently high in both Central Sub-Saharan Africa and East Asia. However, the former demonstrated a growth trend (net drift, 0.5, 95% CI, 0.34 to 0.66), reaching the highest value by 2021 (ASMR: 205.65 per 100,000, 95% UI, 48.15 to 566.13), while the latter demonstrated a clear downward trend (net drift, −0.19, 95% CI, −0.21 to −0.16). (S5 Table in S1 File, S8 Fig in S1 File)

**Fig 3:**
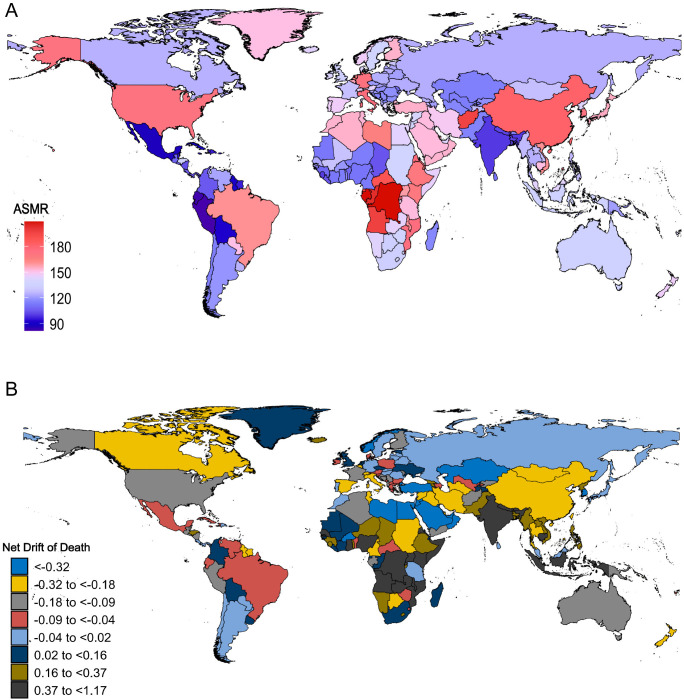
Mortality from Alzheimer’s disease and other dementias among adults aged 55 years and older across 204 countries and territories, 1992 to 2021. A: World map of ASMR for Alzheimer’s disease and other dementias in 2021; B: World map of net drifts for Alzheimer’s disease and other dementias mortality (i.e., estimated annual percentage change in mortality from the APC model). Net drift captures components of the trends attributable to calendar time and successive birth cohorts. ASMR, age-standardized mortality rate; APC, age-period-cohort. In 2021, China, the United States, and Japan had the greatest number of deaths from Alzheimer’s disease and other dementias, while Tokelau and Niue reported the fewest cases. The most significant growth in ASMR was documented in Saint Kitts and Nevis, followed by Kiribati, whereas Palau, the Marshall Islands, and Bahrain saw the largest declines in ASMR. (Interpretation of Fig 3).

In 2021, China, the United States, and Japan accounted for the largest number of deaths due to ADOD, whereas Tokelau and Niue reported the fewest. Gabon, the Democratic Republic of the Congo, and the Republic of the Congo had the highest ASMR, while Peru, Ecuador, and Mexico had the lowest. Although India and Nepal had the lowest ASMR in 1992, both countries nevertheless showed a significant increase from 1992 to 2021. The most significant growth in ASMR was documented in Saint Kitts and Nevis, followed by Kiribati, whereas Palau, Marshall Islands, and Bahrain saw the largest declines in ASMR. (S6 Table in S1 File, Figs 3A and 3B)

### Trends in DALYs of ADOD

From 1992 to 2021, global DALYs attributable to ADOD increased from 14,101,639.87 to 35,625,499.28. ASR of DALYs increased from 2,595.87 per 100,000 (95% UI, 1,173.17 to 5,832.09) in 1992 to 2,621.1 per 100,000 (95% UI, 1,192.88 to 5,766.16) in 2021. The global net drift was 0 (95% CI, −0.01 to 0), indicating an overall stability in global DALYs. Female DALYs exceeded male DALYs, but males demonstrated an upward trend in ASR of DALYs from 1992 to 2021, with a net drift of 0.05 (95% CI, 0.04 to 0.07). (Table 4)

**Table 4:** Global, sex, and SDI trends in DALYs due to Alzheimer’s disease and other dementias among adults aged 55 years and older, from 1992 to 2021.

Characteristics	1992		2021		1992–2021
	DALYs, n (95% UI)	ASR-DALYs per 100 000, n (95% UI)	DALYs, n (95% UI)	ASR-DALYs per 100 000, n (95% UI)	Net Drift (%/year)
Global	14101639.87 (6459705.64 to 31769303.63)	2595.87 (1173.17 to 5832.09)	35625499.28 (16274212.21 to 78478461.82)	2621.1 (1192.88 to 5766.16)	0 (−0.01 to 0)
Sex					
Male	4592937.56 (2075089.91 to 10660013.61)	2107.06 (936.04 to 4894.38)	12202362.18 (5523213.91 to 28099714.48)	2161.17 (967.82 to 4977.54)	0.05 (0.04 to 0.07)
Female	9508702.31 (4365032.26 to 21096167.49)	2886.72 (1312.06 to 6394.16)	23423137.1 (10758455.48 to 50304931.19)	2935.7 (1349.85 to 6306.48)	0.01 (0 to 0.02)
SDI					
High SDI	5384336.44 (2488746.12 to 11766194.75)	2760.32 (1266.98 to 6032.22)	11616224.56 (5340754.1 to 24601306.68)	2678.83 (1243.05 to 5685.58)	−0.05 (−0.07 to −0.04)
High-middle SDI	3688764.12 (1682649.8 to 8378239.47)	2685.15 (1202.32 to 6094.16)	9085513.25 (4192100.49 to 19979625.3)	2800.58 (1286.23 to 6161.8)	0.07 (0.05 to 0.08)
Middle SDI	3112813.37 (1414609.82 to 7111172.97)	2543.57 (1136.51 to 5804.25)	9884977.88 (4551968.54 to 21972268.5)	2645.21 (1207.99 to 5862.83)	0 (−0.02 to 0.02)
Low-middle SDI	1398381.06 (636239.72 to 3210488.09)	1949.64 (877.73 to 4465.75)	3800505.27 (1677834.08 to 8797773.54)	2089.61 (912.95 to 4816.92)	0.22 (0.21 to 0.24)
Low SDI	502223.29 (223512.04 to 1174710.82)	2075.71 (903.79 to 4869.4)	1208919.44 (519188.02 to 2880007.91)	2222.14 (936.36 to 5274.99)	0.22 (0.18 to 0.27)

SDI, socio-demographic index; DALYs, disability-adjusted life-years; ASR, age-standardized rate; 95% UI, 95% Uncertainty Interval; 95% CI, 95% Confidence Interval.

From 1992 to 2021, global DALYs attributable to Alzheimer’s disease and other dementias increased from 14,101,639.87 to 35,625,499.28. Although female DALYs consistently exceeded male DALYs, males exhibited an upward trend in the ASR of DALYs (net drift, 0.05, 95% CI, 0.04 to 0.07). Notably, the high-SDI regions showed a significant decline in ASR of DALYs (net drift, −0.05, 95% CI, −0.07 to −0.04), whereas the low-middle SDI and low-SDI regions experienced marked increases. (Interpretation of Table 4)

ASR of DALYs in the high-SDI regions underwent a substantial decrease during the study period (net drift, −0.05, 95% CI, −0.07 to −0.04). Conversely, the low-middle SDI and low-SDI regions encountered significant increases (low-middle SDI net drift, 0.22, 95% CI, 0.21 to 0.24; low-SDI net drift, 0.22, 95% CI, 0.18 to 0.27). ASR of DALYs for females in the low-SDI regions was substantially greater than in the low-middle SDI regions, and this difference was sustained from 1992 to 2021. (Table 4, S9 Fig in S1 File) The SDI and ASR of DALYs were positively correlated across the 21 regions (ρ = 0.3, *p* < 0.001). (S10 Fig in S1 File) Regionally, South Asia exhibited the most rapid growth in ASR over the past 30 years (net drift, 0.39, 95% CI, 0.36 to 0.42), with Central Sub-Saharan Africa showing the second-largest increase (net drift, 0.36, 95% CI, 0.33 to 0.4). Furthermore, Central Sub-Saharan Africa exhibited the largest ASR of DALYs as of 2021, reaching 3443.11 per 100,000 (95% UI, 1423.61 to 8141.58). (S7 Table in S1 File, S11 Fig in S1 File)

**Fig 4:**
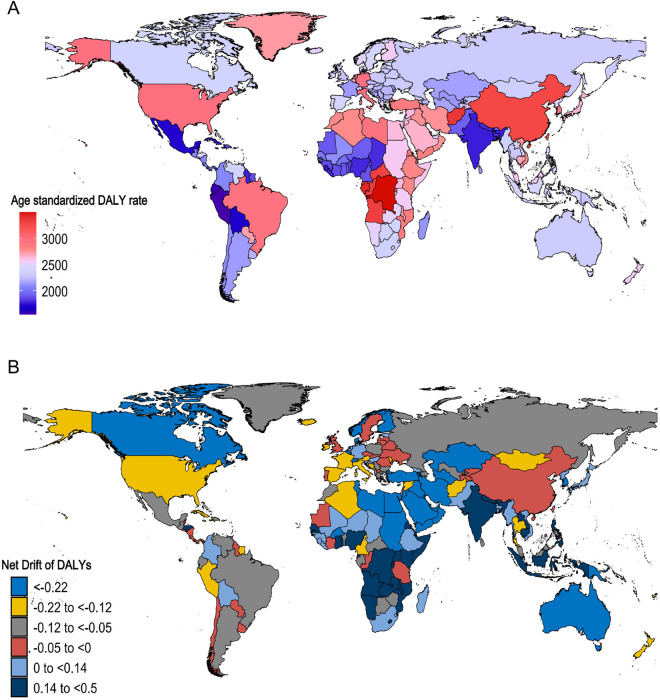
DALYs due to Alzheimer’s disease and other dementias among adults aged 55 years and older across 204 countries and territories from 1992 to 2021. A: World map of ASR of DALYs for Alzheimer’s disease and other dementias in 2021; B: World map of net drifts for Alzheimer’s disease and other dementias DALYs (i.e., estimated annual percentage change in DALYs from the APC model). Net drift captures components of the trends attributable to calendar time and successive birth cohorts. DALYs, disability-adjusted life-years; ASR, age-standardized rate; APC, age-period-cohort. In 2021, the ASR of DALYs showed marked geographic disparities, with the highest burdens observed in the Democratic Republic of the Congo, Gabon, and Afghanistan, while Peru had the lowest rate. During the 1992–2021 period, India showed the steepest rise in ASR of DALYs (net drift, 0.5, 95% CI, 0.46 to 0.54). (Interpretation of Fig 4).

On a national scale, Afghanistan and Gabon recorded the greatest ASR of DALYs in 1992, while India and Peru reported the lowest. By 2021, the countries with higher ASR of DALYs included the Democratic Republic of the Congo, Gabon, and Afghanistan, whereas Peru had the lowest. Between 1992 and 2021, India experienced the most substantial growth in ASR of DALYs (net drift, 0.5, 95% CI, 0.46 to 0.54). (S8 Table in S1 File, Figs 4A and 4B)

### Age-period-cohort effects on incidence and mortality of ADOD

#### APC effects on incidence.

**Fig 5:**
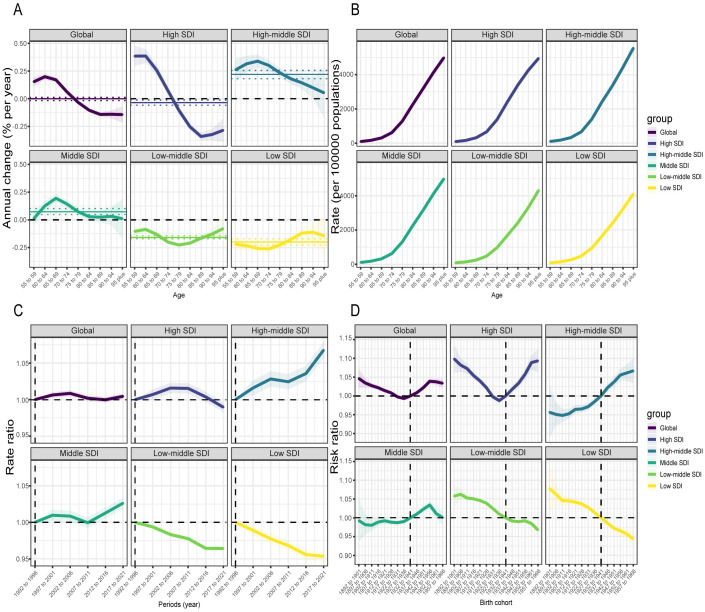
Local drift of ADOD incidence (1992–2021) across SDI quintiles, along with age, period, and birth cohort effects, was analyzed using APC models. A: Local drift of ADOD incidence across nine age groups (1992–2021). The local drift (i.e., annual percentage change of age-specific incidence, % per year) and its associated 95% CIs are indicated by the dots and shaded areas; B: The age effect is illustrated by longitudinal age-specific rates, adjusted for variations across birth cohorts while accounting for period-specific deviations; C: Period effects are quantified as the relative risk of ADOD incidence across time periods, calculated by comparing age-specific rates in each observation period (e.g., 2017–2021) to those in the baseline period (1992–1996); D: Cohort effects are shown as the cohort relative risk of incidence, calculated as the ratio of age-specific rates from the 1892–1901 cohort to the 1957–1966 cohort, with the reference cohort set at 1932–1941. The dots and shaded areas represent the incidence rates or rate ratios and their corresponding 95% CIs. ADOD, Alzheimer’s disease and other dementias; SDI, socio-demographic index.

Globally, the incidence rates for ADOD across all age groups exhibited an overall decline throughout the study period. For age groups under 75–79 years, all local drift coefficients were positive. The highest local drift coefficient was 0.20 in the 60–64 age group, which experienced the most pronounced increase over time. Beyond the age of 75–79 years, the incidence rates declined more sharply with each successive age group, eventually stabilizing around the 85–89 years range. The local drift coefficients approached −0.14. (Fig 5A) Both the general trend and the overall trends for males and females were similar. Notably, males showed their steepest increase at an earlier age, with the local drift coefficient of 0.17 for the 55–59 age group closely approaching their highest local drift coefficient of 0.18. For females, the age group with the most pronounced increase occurred slightly later. (S12 Fig in S1 File) In the five SDI regions, the region with the highest SDI experienced the largest variations in age group trends. The most notable upward trend occurred in the 60–64 age group, while the sharpest decline was recorded in the 85–89 age group, with the local drift coefficient difference reaching 0.73. In the low-middle SDI and low-SDI regions, the decrease in incidence rates after age 85–89 diminished significantly. (Fig 5A)

The age effect curves among various SDI regions were similar, demonstrating a substantial rise in incidence risk after 70–74 years, with the peak risk recorded in those aged 95 and above. In both high-SDI and middle-SDI regions, the incidence rates for individuals aged 95 years and older were approximately 5,000 per 100,000, aligning closely with the global incidence rate. In the high-middle SDI regions, the incidence rate exceeded 5,000 per 100,000, whereas in the low-middle SDI and low-SDI regions, the corresponding rates were below 5,000 per 100,000. (Fig 5B)

The period effect indicated that global incidence rates fluctuated slightly but remained relatively stable overall. In the high-middle SDI and middle-SDI regions, incidence rates displayed a consistent upward trajectory, reflecting predominantly unfavorable period risks. In contrast, incidence rates in the low-middle SDI and low-SDI regions demonstrated a consistent decline, indicative of favorable period risks. The high-SDI regions revealed a dynamic pattern, different from the single trend observed in other SDI regions. Although the high-SDI regions generally experienced unfavorable period risks, their incidence rate declined after 2016 when compared to the 1992–1996 reference period. In the 2017–2021 period, the RR for these regions was 0.9896 (95% CI, 0.9829 to 0.9964), suggesting a shift toward favorable period risks. (Fig 5C)

After controlling for age and period effects, global incidence risk initially increased, followed by a decrease, and ultimately exhibited a sustained upward trend after the 1932–1941 cohort, consistent with the incidence pattern observed in the high-SDI regions. Relative to the 1932–1941 cohort, the risk of ADOD was comparatively smaller in the high-middle SDI and middle-SDI regions for earlier cohorts. After this cohort, the high-middle SDI regions displayed a sustained increase, while the middle-SDI regions showed a gradual reduction in risk among individuals born after the 1947–1956 cohort. In contrast to the reference cohort, individuals born prior to that cohort in low-middle SDI and low-SDI regions had a greater risk of developing ADOD. For individuals born after that cohort, the risk decreased and continued to demonstrate a declining trend. (Fig 5D)

#### APC effects on mortality.

**Fig 6:**
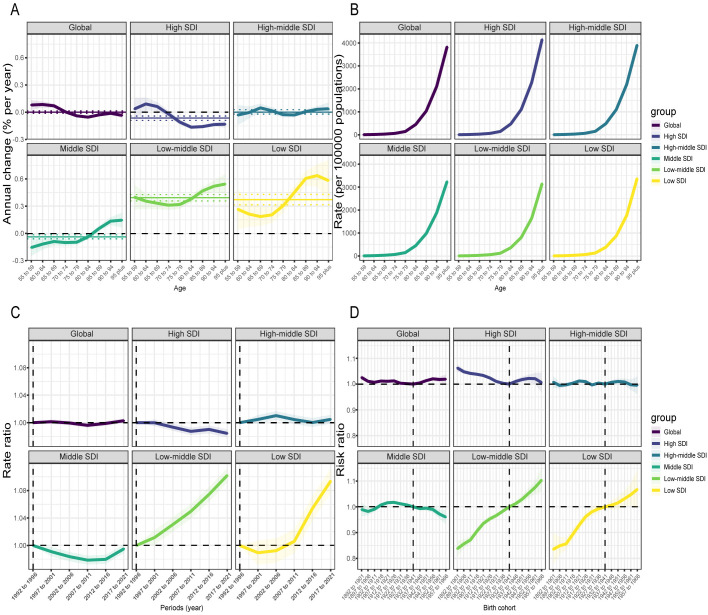
Local drift of ADOD mortality (1992–2021) across SDI quintiles, along with age, period, and birth cohort effects, was analyzed using APC models. A: Local drift of ADOD mortality across nine age groups (1992–2021). The local drift (i.e., annual percentage change of age-specific mortality, % per year) and its associated 95% CIs are indicated by the dots and shaded areas; B: The age effect is illustrated by longitudinal age-specific rates, adjusted for variations across birth cohorts while accounting for period-specific deviations; C: Period effects are quantified as the relative risk of ADOD mortality across different time periods, calculated by comparing age-specific rates in each observation period (e.g., 2017–2021) to those in the baseline period (1992–1996); D: Cohort effects are shown as the cohort relative risk of mortality, calculated as the ratio of age-specific rates from the 1892–1901 cohort to the 1957–1966 cohort, with the reference cohort set at 1932–1941. The dots and shaded areas represent the incidence rates or rate ratios and their corresponding 95% CIs. ADOD, Alzheimer’s disease and other dementias; SDI, socio-demographic index.

The fluctuations in mortality across age groups older than 55 were relatively small globally, while mortality rates in younger age brackets exhibited an upward trend. After the age of 70–74 years, the local drift values for global mortality began to decline below zero. (Fig 6A) The mortality trend for females aligned with the overall trend, whereas males exhibited greater heterogeneity. The mortality rate for males rose in all age groups, with the most significant increases occurring in the 85–89 years and 90–94 years groups, followed by those aged 95 and above. (S13 Fig in S1 File) The mortality trends across age groups differed depending on the SDI regions. The variation in overall mortality rates in high-middle SDI regions was comparatively lower than that observed in the remaining SDI regions. Although mortality rates in the low-SDI regions exhibited an upward trend akin to that in the low-middle SDI regions, the differences in local drift coefficients across age brackets were larger compared to the low-middle SDI regions. Furthermore, the range of variation in the low-SDI regions was the most pronounced across all SDI regions. The trend trajectories of the high-SDI and middle-SDI regions exhibited opposite directions. In the middle-SDI regions, the mortality rate for every age group above 80–84 years exhibited a rising pattern, whereas in the high-SDI regions, these same age groups demonstrated a clear decline. (Fig 6A)

The age effect demonstrated that the mortality risk of ADOD among various SDI regions followed a similar trend, with the lowest risk identified in individuals aged 55–59 years and an increase in mortality risk with advancing age. When contrasted with other SDI regions, the mortality rate for individuals over 95 years of age in the high-SDI regions was the most elevated, exceeding 4,000 per 100,000. (Fig 6B)

Mortality rates in the high-middle SDI and low-middle SDI regions exhibited an upward trend, reflecting adverse period risks, particularly in the low-middle SDI regions, where the mortality rate consistently exhibited rapid growth. In the high-SDI regions, the mortality rate ceased to remain stable after 1997–2001 and began a steady decline. In the middle-SDI regions, the mortality rate displayed a declining trend after 1992–1996, which progressively weakened after 2012–2016. Mortality rates in the low-SDI regions demonstrated a general upward trend. Notably, from 2002 to 2006 onward, the RR in the low-SDI regions surpassed 1, signaling a shift from favorable to unfavorable period risks. Furthermore, this upward trend accelerated after 2007–2011. (Fig 6C)

The mortality risk trends for global continuous birth cohorts before the 1932–1941 cohort were similar to those observed in the high-middle SDI regions, characterized by minor fluctuations and a modest upward trend. After this reference cohort, the global trend continued on a steady upward trajectory, whereas the high-middle SDI regions observed a slight decline following the 1952–1961 cohort. In the high-SDI regions, individuals born before the 1932–1941 cohort experienced an increasing mortality risk, though this upward trend gradually diminished. Following the reference cohort, the mortality risk in this region rose, but the upward trend diminished after the 1952–1961 cohort. In the middle-SDI regions, individuals born after the 1902–1911 cohort experienced an increasing mortality risk, while individuals born following the 1932–1941 cohort showed a lower mortality risk. The mortality risk patterns in low-middle SDI and low-SDI regions were strikingly similar. In comparison to individuals born before the reference cohort, those born after this cohort exhibited a continuous upward trend in mortality risk. (Fig 6D)

### Predictions of the global disease burden of ADOD until 2046

**Fig 7:**
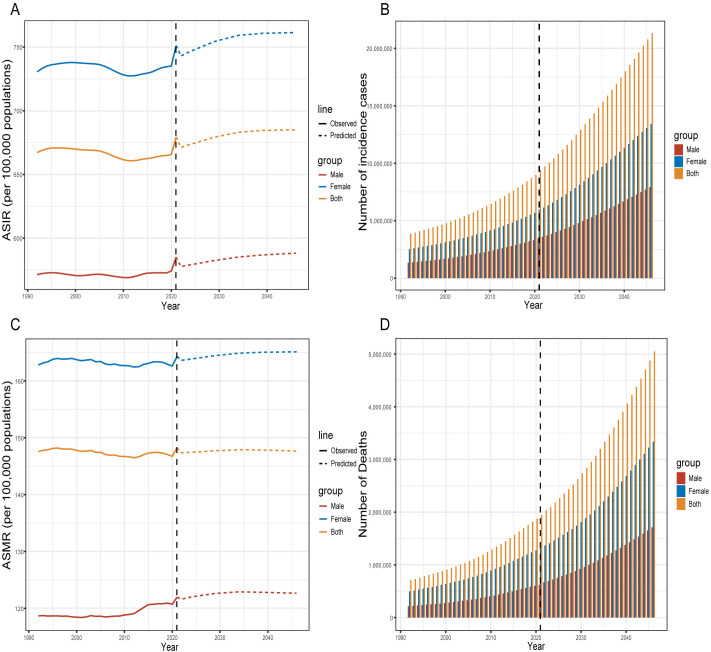
Future projections of ASIR, ASMR, and the number of ADOD cases (1992–2046). A: Projected ADOD incidence rates (1992–2046) for both sexes combined, males, and females in the population aged 55 years and older; B: Projected incident cases (1992–2046) for both sexes combined, as well as separately for males and females, in the population aged 55 years and older; C: Projected ADOD mortality rates (1992–2046) for both sexes combined, males, and females in the population aged 55 years and older; D: Projected number of deaths (1992–2046) for both sexes combined, as well as separately for males and females, in the population aged 55 years and older. Solid lines represent observed ASIR and ASMR, while dashed lines indicate ASIR and ASMR projections based on the Nordpred model. ASIR, age-standardized incidence rate; ASMR, age-standardized mortality rate; ADOD, Alzheimer’s disease and other dementias.

We employed the Nordpred software package to forecast the future global trend of ADOD and validated these predictions using BAPC. The results indicated that the predicted trends for ADOD’s ASIR and ASMR were consistent between the Nordpred software package and BAPC. (S9 and S10 Tables in S1 File, S14A–S14D Figs in S1 File) Projections show that by 2046, the ASIR for individuals aged 55 and older will reach 684.96 per 100,000. The overall number of new ADOD cases globally is projected to increase, with an estimated 21,334,937 cases by 2046. Both new ADOD cases in females and males exhibit a steady upward trend, with the count of new cases in females consistently exceeding that in males. (S9 Table in S1 File, Figs 7A and 7B)

The prediction results indicate that the global ASMR increases from 147.34 per 100,000 in 2022 to 147.64 per 100,000 in 2046, reflecting a slight upward trend overall. At the gender level, the ASMR trends for both males and females are expected to be similar, with both projected to exhibit a small increase. The forecasts suggest that ADOD fatalities will keep increasing, with female ADOD fatalities remaining greater than those of males. (S10 Table in S1 File, Figs 7C and 7D)

## Discussion

Globally, the ASIR, ASPR, ASMR, and ASR of DALYs in females aged 55 and above were higher than those in males. A study from Sweden suggested that these higher values in females, compared to males, may have been attributed to women’s longer life expectancy, which provided them with more opportunities for participation in ADOD screening [26]. Furthermore, women may also utilize healthcare services more frequently due to menopause and reproductive health issues, which makes them more susceptible to a diagnosis of ADOD compared to men. At the same time, specific physiological factors in women, such as later onset of menstruation, earlier menopause, elevated estradiol levels, and higher levels of S-nitrosylation of C3 in the brain, are linked to a higher risk of ADOD [27–29]. Depression, from a psychological perspective, is a key risk factor for the development of ADOD [30]. Previous studies have indicated that women face a significantly higher risk of depression compared to men. This risk is attributed to hormonal fluctuations during life stages such as postpartum and menopause, the inhibitory effects of estrogen on sympathetic adrenal responses, and a stronger tendency towards rumination [31]. The interplay of these factors may have contributed to a higher incidence of ADOD diagnosis in women. Alongside physical and psychological factors, socio-environmental determinants may further exacerbate the disease burden in women. Women in low-SDI regions consistently demonstrated higher disease burden than those in low-middle SDI regions, particularly in ASMR and the ASR of DALYs. This indicates that structural socioeconomic disparities not only amplify the unequal distribution of health resources but also gradually weaken women’s resilience to chronic neurodegenerative diseases. Research by Gong et al. lends support to this perspective [32]. It suggests that socioeconomic deprivation and the associated inequities in resource allocation may be key factors influencing both ADOD risk and women’s health outcomes. Meanwhile, males exhibited greater net drift for all indicators than females, indicating that the ADOD disease burden grew at a quicker rate in males. This may be attributed to men’s greater propensity to adopt unhealthy lifestyles, such as frequent alcohol consumption and smoking [33,34].

Across the five SDI regions, only the high-SDI regions exhibited a declining trend in both mortality and DALYs for individuals aged 55 and older, signifying that the disease burden of ADOD had been decreasing in these regions. This trend is likely the result of the convergence of improved healthcare infrastructure, broader health policy coverage, and advancements in public health literacy. High-SDI regions have developed comparative advantages, including early disease detection, standardized therapeutic interventions, and integrated care management. This progress is driven by continuous optimization of clinical technologies, strengthened health promotion initiatives, and the rigorous enforcement of public health policies. Increased public health awareness plays a crucial role in the early detection and intervention of ADOD, thereby improving therapeutic outcomes. Such improvements underscore the essential need for systemic, integrative disease control strategies to effectively reduce the overall disease burden [35].

Between 1992 and 2021, significant regional differences in ASIR, ASPR, and ASMR were observed. In the East Asia region, both incidence and prevalence rates increased rapidly. However, the mortality rate declined, particularly in China and Japan, which are representative of the region. China hosts the largest elderly population globally, with approximately 173 million individuals aged 65 and above [36]. The rising incidence and prevalence of ADOD in China may be associated with the accelerating aging population and increased life expectancy. Continuous efforts in ADOD control may have contributed to the observed reductions in DALYs and mortality. China’s urban-rural integrated basic medical insurance scheme has enhanced healthcare access and affordability for ADOD patients by expanding coverage and alleviating financial burdens [37]. In 2019, the medical consultation rate among AD patients in China reached 77.43% [38]. This suggests that expanded healthcare coverage and improved public health literacy may have contributed to better disease outcomes. In 2021, the incidence and prevalence rates of ADOD in individuals aged 55 and older in Japan were not the highest. However, both indicators demonstrated an upward trend, placing them among the top, while the mortality rate showed a slight decline. Overall, Japan’s efforts in managing ADOD have received positive feedback [39]. Contrasting with China, the DALYs for ADOD in Japan showed an upward trend. This suggests that, in the context of an aging population, the impact of ADOD on the quality of life among middle-aged and elderly individuals is steadily increasing. This also suggests that, even in countries with well-established healthcare systems, extreme aging continues to pose a significant challenge in managing ADOD [40,41]. Future governance strategies should not only focus on advancing medical technologies but also emphasize strengthening the development of care and long-term care systems for the elderly. South Asian countries such as India, Bhutan, and Nepal, and Sub-Saharan African countries like Ghana and the Democratic Republic of Congo have shown a rapid increase in ASMR and ASR of DALYs, reflecting a heavy ADOD disease burden. This may be due to lower education levels and weaker health awareness, leading to insufficient prevention, treatment, and awareness of ADOD’s potential consequences [42,43].

The age effect demonstrated that the incidence and mortality risk of ADOD rose with advancing age. The interaction between Aβ and phosphorylated tau (P-tau) causes cognitive dysfunction in ADOD patients [44]. Alleviating brain aging can improve cognitive impairment caused by Aβ and P-tau, highlighting the key role aging plays in the pathological and physiological processes of ADOD [41]. Additionally, chronic multimorbidity is highly prevalent in the elderly population. Studies confirm that chronic diseases such as diabetes [45], hypertension [46], and myocardial infarction [47] are significantly linked to a heightened risk of MCI, which is considered a transitional stage to ADOD [11]. Consequently, the cumulative burden of chronic disease with age works in conjunction with brain aging to further increase the risk of developing ADOD. In the future, strengthening the prevention and management of chronic diseases will be crucial. Although ADOD predominantly affects the elderly population, our research revealed that the incidence rate among individuals aged 60–64 years showed the highest growth trend globally, while the incidence rate declined after 75–79 years. This suggests that the disease burden is gradually shifting towards the ‘relatively younger’ elderly population. With advances in screening tools and improvements in public health coverage, more cases are being identified at earlier stages in a timely manner [48]. Therefore, extending the age range for health risk screening and intervening in the early stages of ADOD development is critical for effectively preventing further disease progression. Meanwhile, individuals aged 95 and above have been identified as the group with the highest mortality risk from ADOD. Although the mortality risk is already elevated in the elderly population, a study further shows that once individuals develop ADOD, their mortality risk increases 5.9 times compared to their peers without the condition [49]. This further emphasizes the need to enhance functional assessments and quality of life interventions for the ultra-elderly population.

From a period effect perspective, after 1992–1996, the incidence risk of ADOD in high-SDI, high-middle SDI, and middle-SDI regions showed a significant increase. This may be related to the worsening levels of environmental pollution during rapid industrialization. Multiple studies have confirmed the association between air particulate matter, such as PM2.5, and neurodegenerative changes [50,51]. The reduced ADOD incidence risk in the high-SDI regions after 2012–2016 may be attributed to sustained investment in environmental pollution prevention research and the implementation of effective environmental management measures, which have significantly improved environmental quality [52]. Metabolic syndrome has been identified as a potential risk factor for AD. A study indicated that individuals with metabolic syndrome have an 11.48-fold increased risk of developing AD compared to those without the condition [53]. In high-middle SDI regions and middle-SDI regions, diets rich in sugar and energy contribute to elevated BMI [54], which may lead to metabolic abnormalities and increase the risk of AD [53]. This may account for the increasing incidence rate of ADOD in these regions. The widespread prevalence of ultra-processed food (UPF), a consequence of food industrialization, represents a significant factor contributing to the issue [55]. The proportion of UPF in the dietary patterns of high- and middle-income countries has increased over time [56]. This increased intake has been associated with cognitive decline [57]. A study involving 72,083 participants further confirmed that a high intake of UPF significantly elevates the risk of ADOD [56]. We hypothesize that the increasing incidence of ADOD results from the combined effects of metabolic abnormalities, unbalanced dietary patterns, and the consumption of industrialized foods.

Lower levels of perceived socio-environmental support represent a significant factor influencing the increased mortality risk in ADOD patients [58]. The impact is particularly significant in regions with low-SDI and low-middle SDI values. Insufficient social support reduces the life expectancy of patients with ADOD. The uneven distribution of healthcare resources and limited accessibility further hinder advancements in treatment and prognosis. Compared to high-SDI regions, these areas encounter systemic disparities in early screening, comorbidity management, and long-term care support. As a result, the quality of care following an ADOD diagnosis is generally lower. The observed paradox of stable ADOD incidence and rising mortality in low-SDI regions may be attributed to a range of factors. These include the use of inadequate screening tools, the uneven distribution of specialist resources, and the incomplete implementation of standardized diagnostic protocols [59], all of which may result in missed or delayed diagnoses. Cultural factors may also contribute. For example, limited public awareness of dementia, stigma associated with cognitive impairment, and prevailing preferences for family-based care can influence care-seeking behavior and contribute to delays in timely intervention [60]. The decline in ADOD mortality in the high-SDI regions likely reflects improvements in early detection, comorbidity management, and access to long-term care. In these regions, population ageing is one of the main drivers of the increasing prevalence and burden of ADOD. Early public health initiatives in these settings have enabled earlier dementia identification and care planning. By comparison, low-SDI regions remain in the early stages of both population ageing and dementia awareness. With ongoing ageing, limited screening infrastructure and poor public awareness may contribute to diagnostic delays, suboptimal care, and widening disparities in survival outcomes. In these regions, improvements in recognition and diagnostic capacity may result in higher reported mortality, indicative of more complete case ascertainment. Furthermore, the low-SDI regions experience a higher incidence of AIDS [61]. HIV infection is an etiological factor in the development of ADOD [62]. To enhance the current situation, it is crucial to strengthen primary healthcare systems, particularly in regions with limited medical resources. Coordinate HIV prevention and control efforts with the enhancement of the ADOD diagnostic system. Specific measures should focus on enhancing the diagnostic and treatment capabilities of healthcare professionals and ensuring a consistent supply of antiretroviral drugs and ADOD treatment medications. Additionally, developing a community support system through caregiver training and regular follow-ups is essential.

The cohort effect revealed that the incidence risk of ADOD significantly rose in birth cohorts after 1932–1941 in the high-SDI regions. This could be attributed to lifestyle changes linked to rapid urbanization, such as increased sedentary behavior and the prolonged accumulation of mental stress [63–65]. According to WHO data, middle- and low-income nations account for 91% of premature deaths linked to air pollution [66]. In low-middle SDI and low-SDI regions, air pollution and the lack of sufficient industrial structural transformation may represent significant environmental factors. These factors contribute to the rising mortality rate from ADOD in subsequent birth cohorts [67]. These regions exhibit a relatively low reliance on clean energy and environmental protection technologies, coupled with weak ecological health protection capabilities. Consequently, there is an urgent need to implement structural reforms at the policy level.

Building on the above research findings, we believe that targeted prevention and control measures can be developed in the following areas going forward. First, it ought to be considered that gender differences in physiological structure and disease risk factors exist, with a particular emphasis on addressing the needs of the female population. Improving women’s education levels, promoting health literacy, and enhancing the accessibility of healthcare services could help mitigate the burden of ADOD, especially in the low-SDI regions. Second, we propose establishing a comprehensive “Prevention-Intervention-Care” health management system that spans the entire care cycle. This approach aims to mitigate the risk of ADOD onset by implementing robust early warning systems and optimizing health screening strategies. Concurrently, enhance the tiered care framework for the aging population, fortifying essential daily living support and expanding long-term care services. Third, encourage the adoption of healthier eating habits in regions with higher SDI. Guide residents to reduce their consumption of sugar-sweetened beverages and UPF, while promoting a shift towards natural, minimally processed alternatives to mitigate associated health risks. Fourth, enhancing the coverage and quality of public health services, alongside strengthening social support networks, is recommended, particularly in regions with lower SDI. Fifth, to promote physical and mental well-being, adopting healthy lifestyle habits is recommended, such as reducing sedentary behavior, strategically planning physical and leisure activities, and focusing on stress management. Sixth, governments in lower SDI regions should actively promote the development of green industries, facilitate the adoption of low-pollution technologies in industry and infrastructure, and advocate for the use of clean energy.

However, there are several limitations to this research. First, the GBD 2021 lacks a clear classification of ADOD subtypes, which may limit our ability to analyze the disease burden of specific subtypes. Second, low-income countries often lack epidemiological data and raw medical records due to underdeveloped healthcare systems and limited resources. Furthermore, the quality and validity of data from these regions in the GBD 2021 cannot be assured, which may affect trend analysis using the APC model. Third, the GBD 2021 does not encompass data from all regions and populations, meaning our findings reflect only a general overview of specific areas. Fourth, although this study used the Nordpred and BAPC models to project and validate the global burden of ADOD, they have limited ability to capture regional disparities and contextual complexities. Revisions in diagnostic criteria and the expansion of screening coverage may contribute to an apparent increase in reported incidence and prevalence. Furthermore, demographic uncertainties, such as population ageing and increased life expectancy, may undermine the reliability of long-term projections. Future research should aim to improve and build upon the aforementioned aspects.

## Conclusion

ADOD continues to constitute a major component of the global neurological disease burden, and by 2046, the burden of ADOD among individuals aged 55 and above is projected to remain significant. Although the global ADOD burden has moderately declined over the past three decades, significant regional heterogeneity persists. Countries should develop and implement targeted public health interventions for high-risk populations, tailored to the epidemiological characteristics and specific risk factors of ADOD within their regions, to mitigate its disease burden.

## Supporting information

S1 File All supplementary figures, tables, and materials. The files include fourteen figures (S1–S14 Figs), ten tables (S1–S10 Tables), and supplementary materials.
(PDF)
