# Supplementary material for: Global trends in Alzheimer’s disease and other dementias in adults aged 55 and above (1992–2021): An age-period-cohort analysis based on the GBD 2021
Source: PLoS One. 2025 Aug 29;20(8):e0331204. doi: 10.1371/journal.pone.0331204 (PMC12396692; doi:10.1371/journal.pone.0331204)
Supplement: S1 File — The files include fourteen figures (S1–S14 Figs), ten tables (S1–S10 Tables), and supplementary materials. (PDF) [file pone.0331204.s001.pdf]

Global Trends in Alzheimer's Disease and Other Dementias in Adults Aged 55 and Above (1992–2021): An Age-Period-Cohort Analysis Based on the GBD 2021

Qianqian Zhang[1¶], Yanwen Deng[2¶], Mo Xue[1], Zihan Ni[3], Guangyan Luo[1], Kan Tian[3*]

[1]School of Health Economics and Management, Nanjing University of Chinese Medicine, Nanjing, China

[2]School of Medicine, Nanjing University of Chinese Medicine, Nanjing, China

[3]School of Elderly Care Services and Management, Nanjing University of Chinese Medicine, Nanjing, China

Qianqian Zhang, Yanwen Deng contributed equally to this work and share first authorship.

*** Correspondence:**

Kan Tian

Email: tiankan@njucm.edu.cn

Supporting Information

This document contains Supplementary Figures S1, S2, S3, S4, S5, S6, S7, S8, S9, S10, S11, S12, S13 and S14; Supplementary Tables S1, S2, S3, S4, S5, S6, S7, S8, S9 and S10; and Supplementary Materials.

Figures

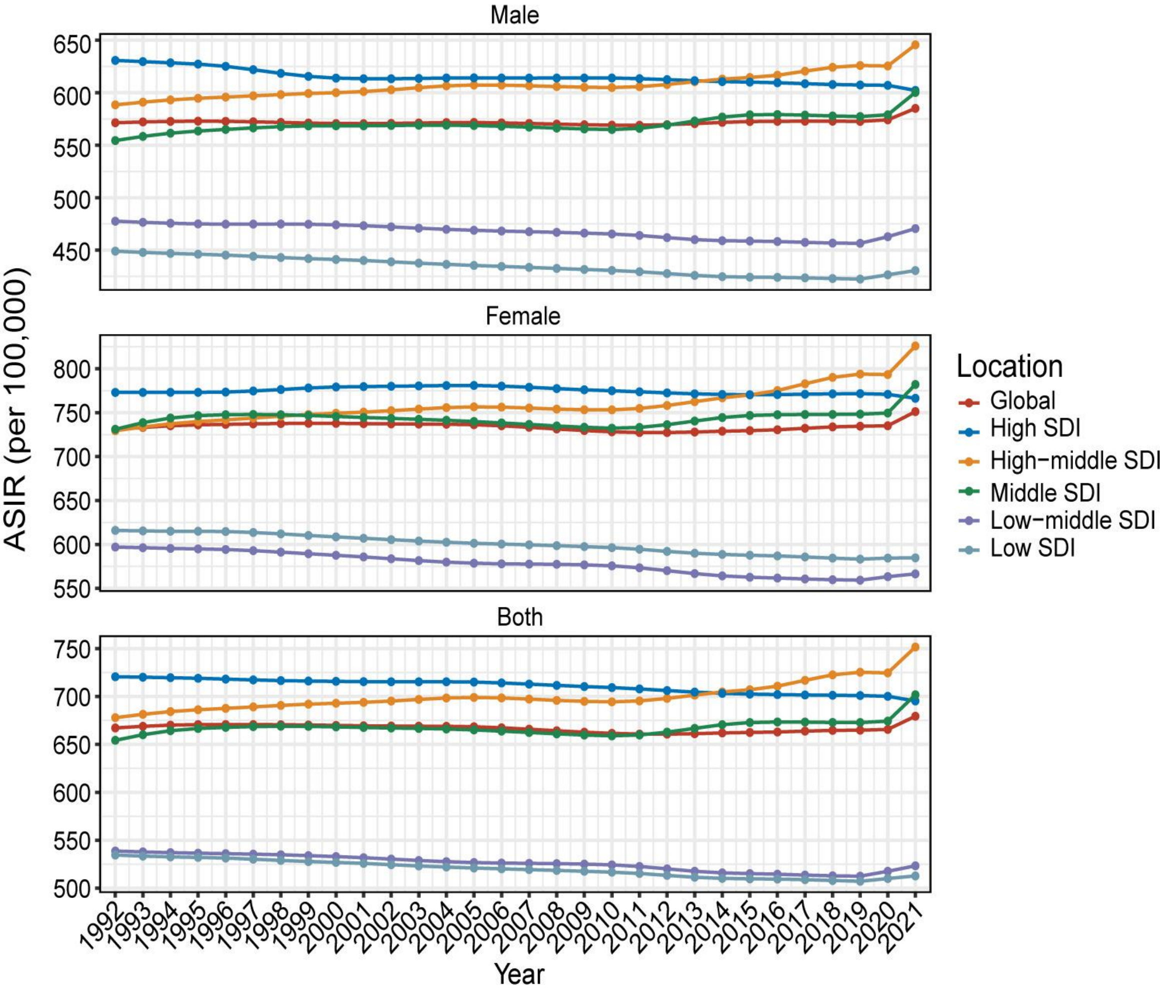

S1 Fig. ASIR of ADOD globally and across SDI regions for both sexes, males, and females aged 55 and older (1992–2021). ASIR, age-standardized incidence rate; ADOD, Alzheimer's disease and other dementias; SDI, socio-demographic index.

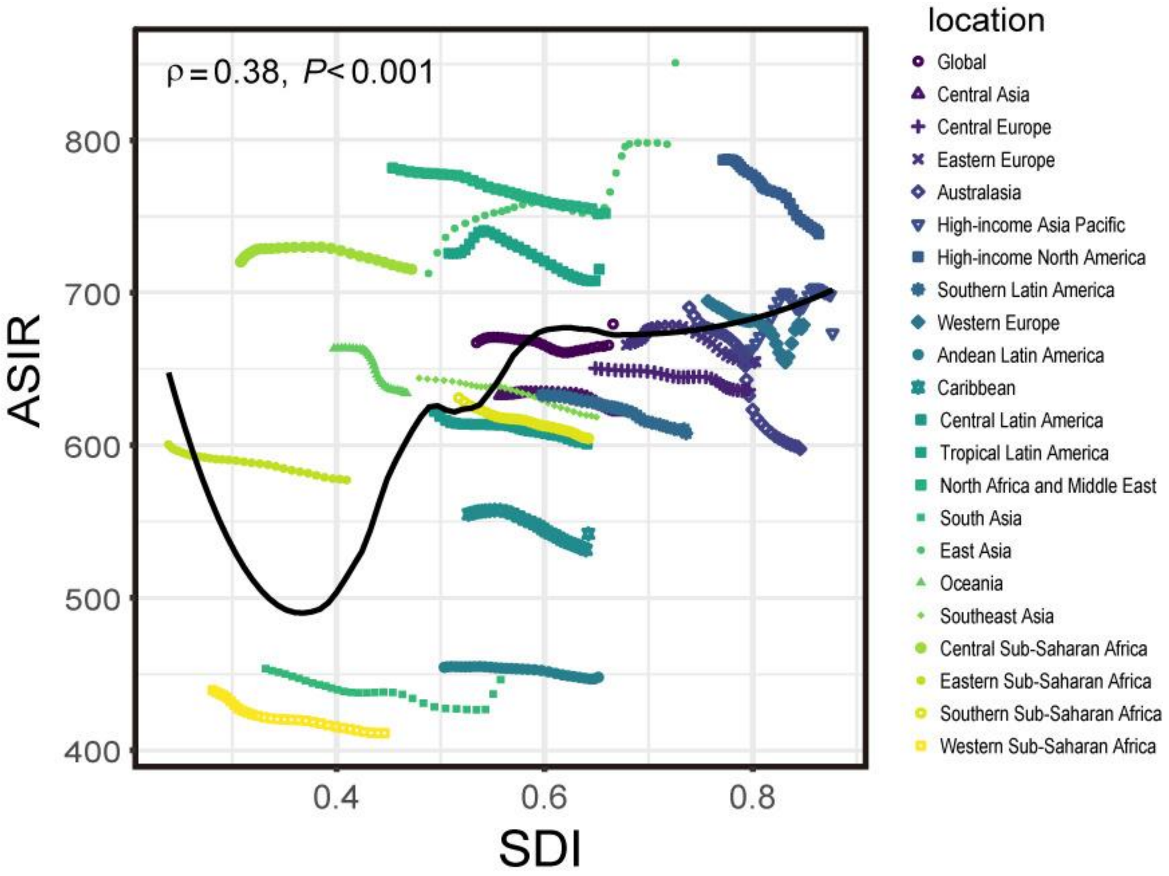

S2 Fig. Spearman correlation between ASIRs of Alzheimer's disease and other dementias and SDI levels across regions. ASIR, age-standardized incidence rate; SDI, socio-demographic index.

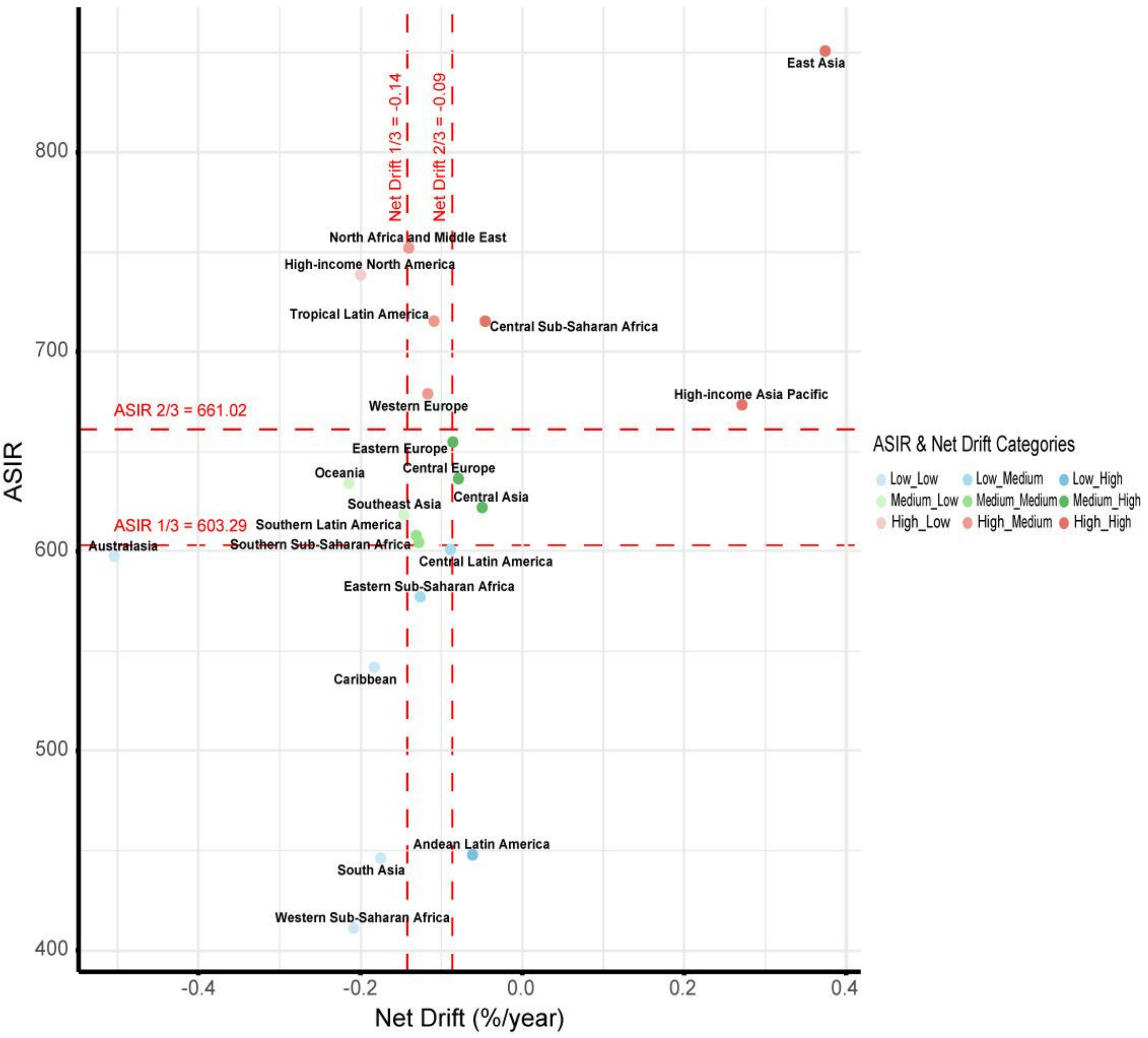

S3 Fig. Classification of 21 regions in 2021 based on ASIRs and net drift. ASIR, age-standardized incidence rate.

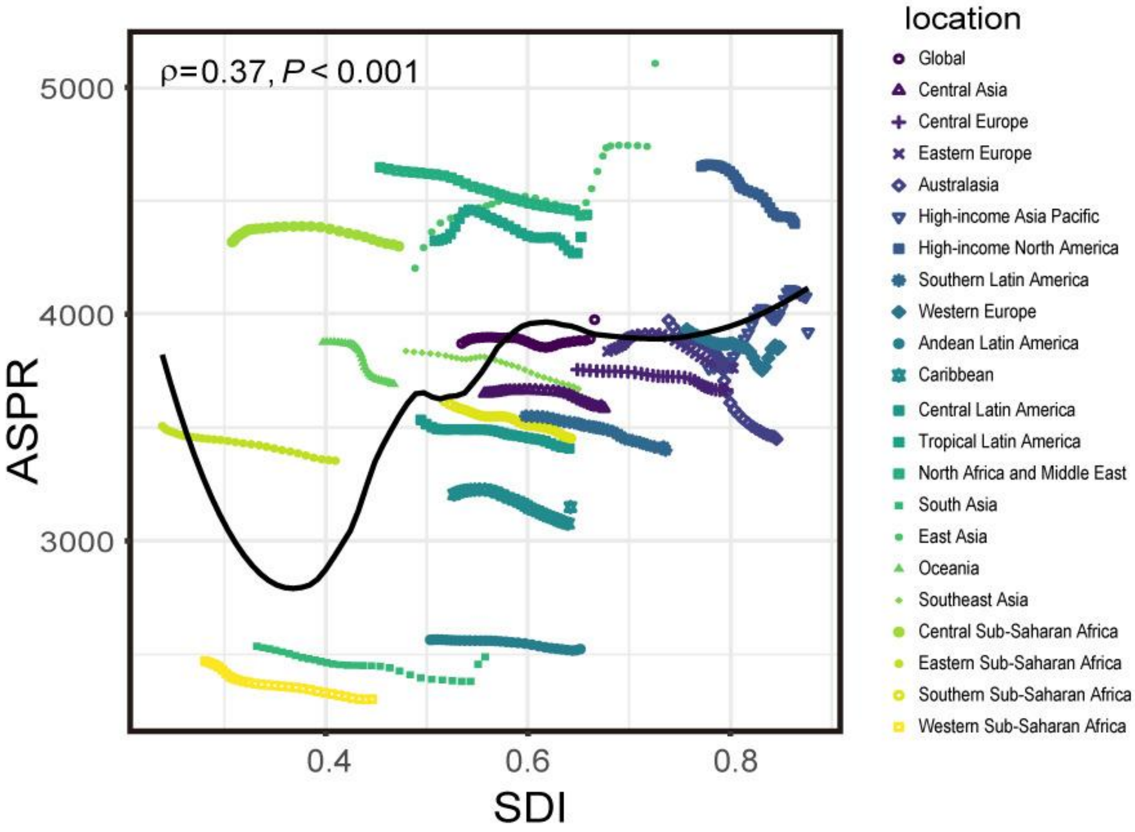

S4 Fig. Spearman correlation between ASPRs of Alzheimer's disease and other dementias and SDI levels across regions. ASPR, age-standardized prevalence rate; SDI, socio-demographic index.

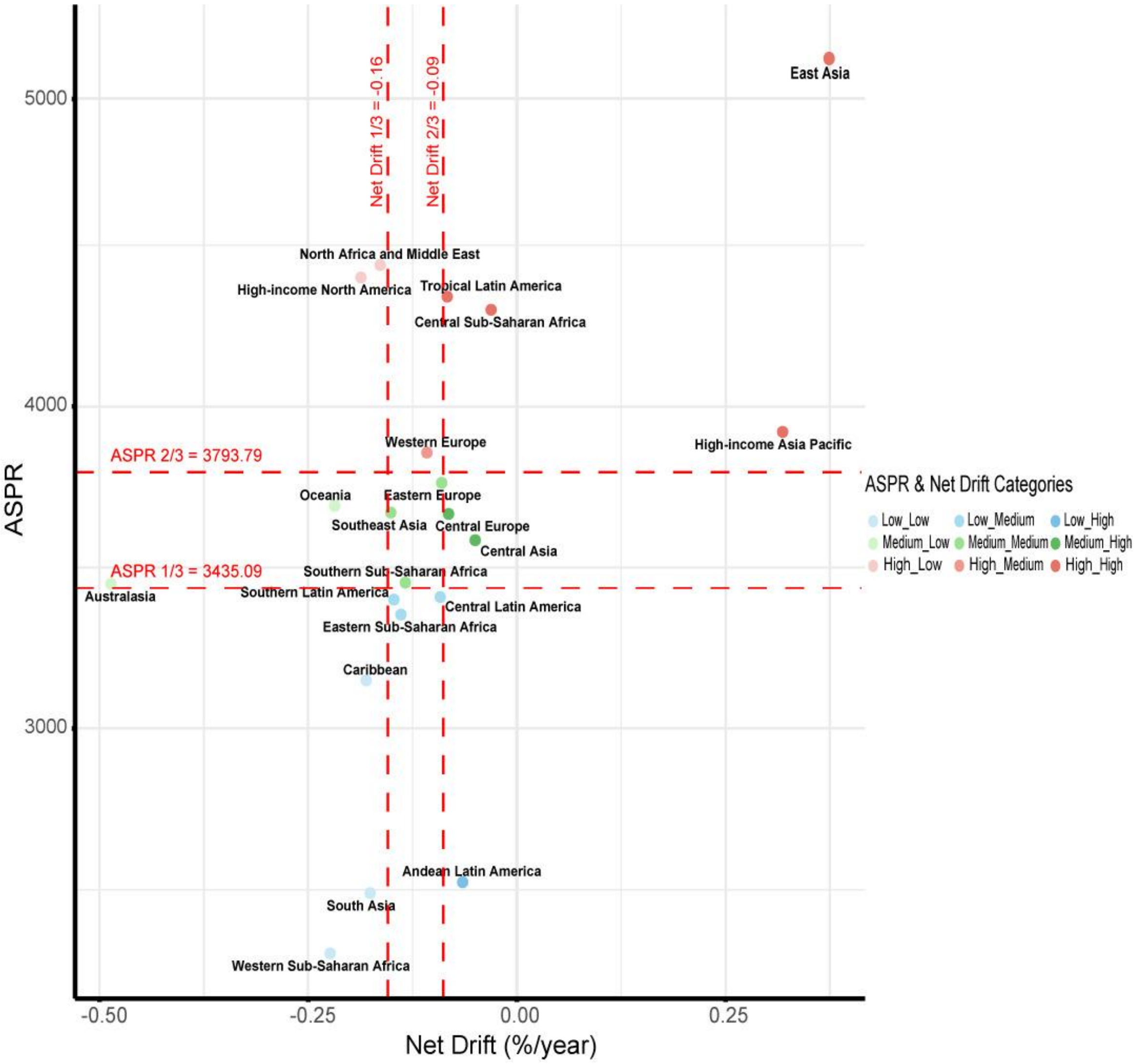

S5 Fig. Classification of 21 regions in 2021 based on ASPRs and net drift. ASPR, age-standardized prevalence rate.

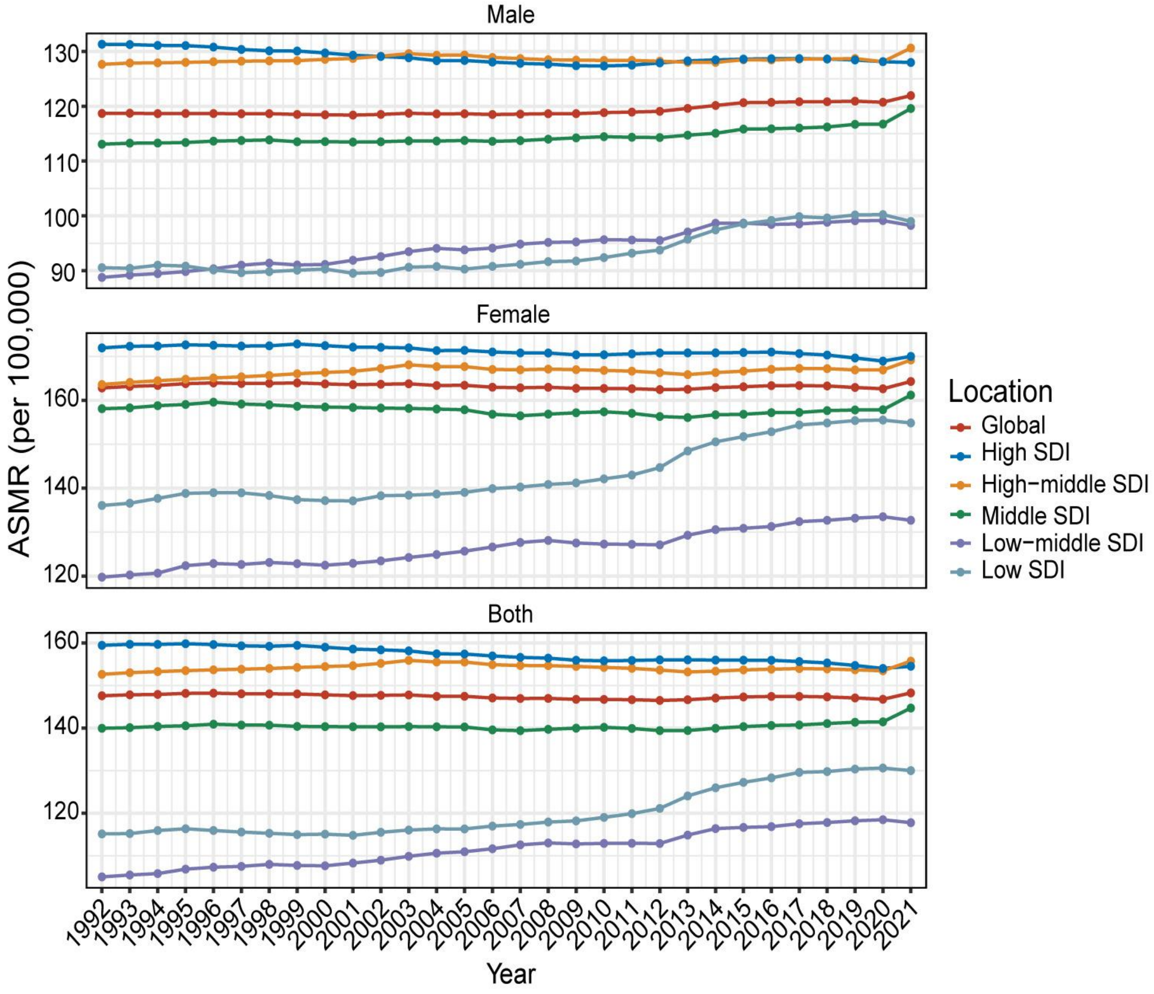

S6 Fig. ASMR of ADOD globally and across SDI regions for both sexes, males, and females aged 55 and older (1992–2021). ASMR, age-standardized mortality rate; ADOD, Alzheimer's disease and other dementias; SDI, socio-demographic index.

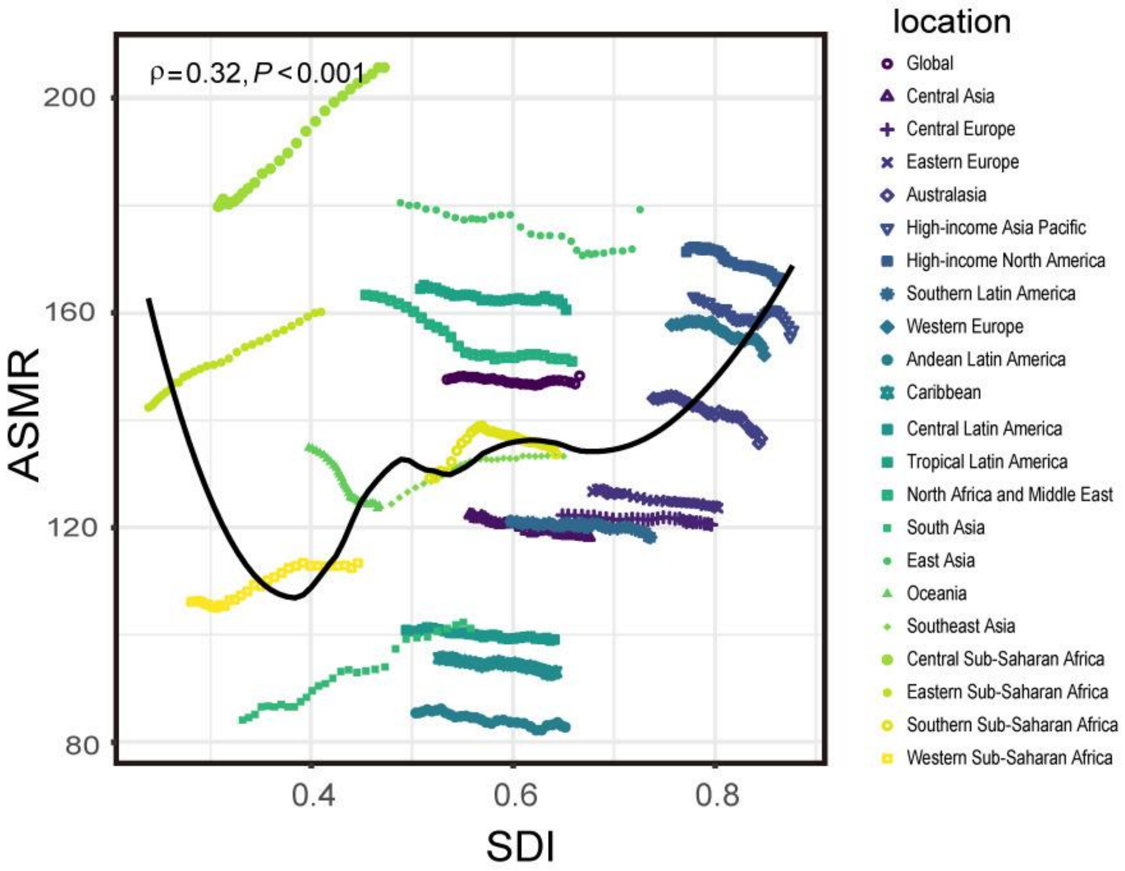

S7 Fig. Spearman correlation between ASMRs of Alzheimer's disease and other dementias and SDI levels across regions. ASMR, age-standardized mortality rate; SDI, socio-demographic index.

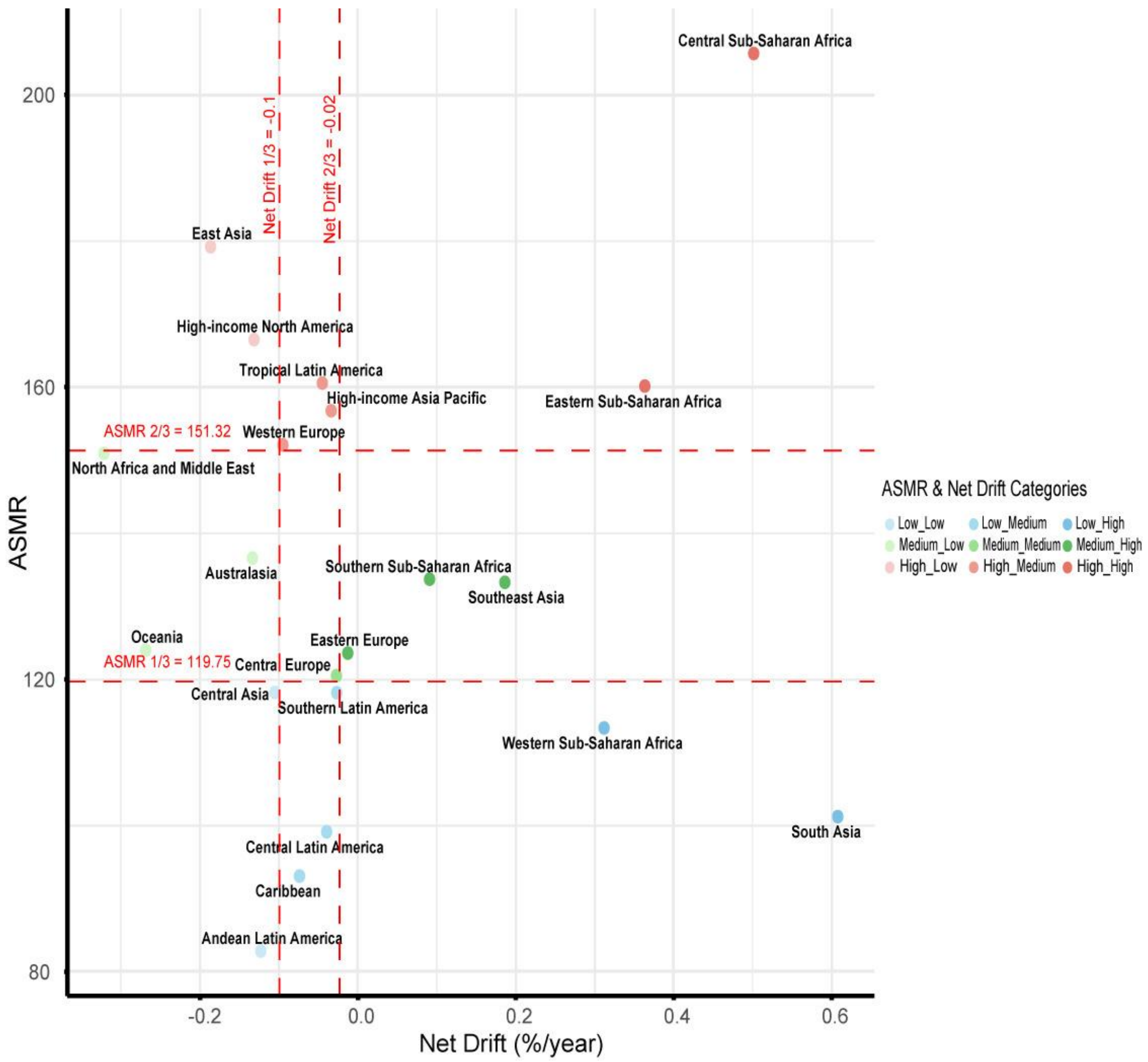

S8 Fig. Classification of 21 regions in 2021 based on ASMRs and net drift. ASMR, age-standardized mortality rate.

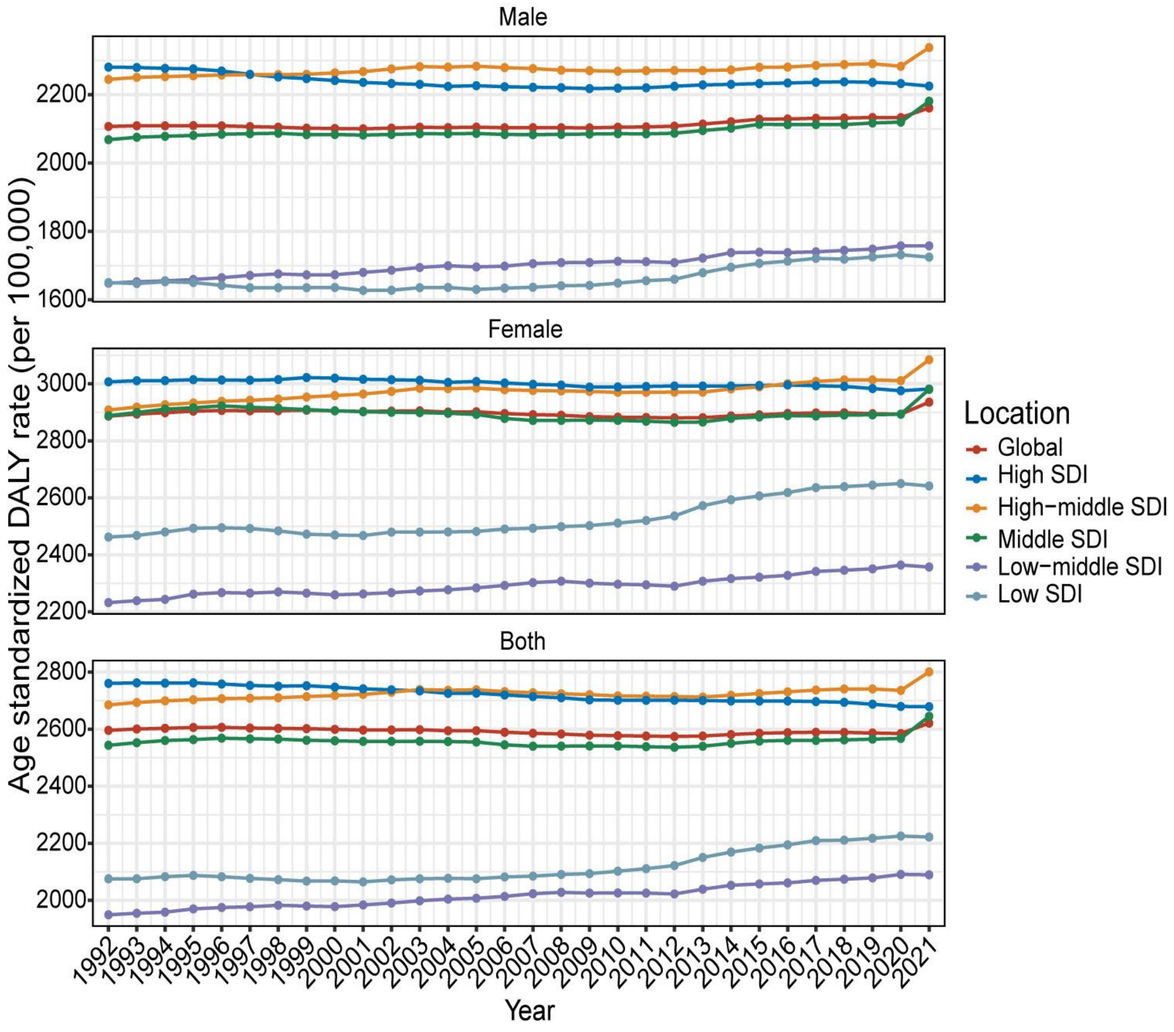

S9 Fig. Age-standardized DALY rate of ADOD globally and across SDI regions for both sexes, males, and females aged 55 and older (1992–2021). DALY, disability-adjusted life-year; ADOD, Alzheimer's disease and other dementias; SDI, socio-demographic index

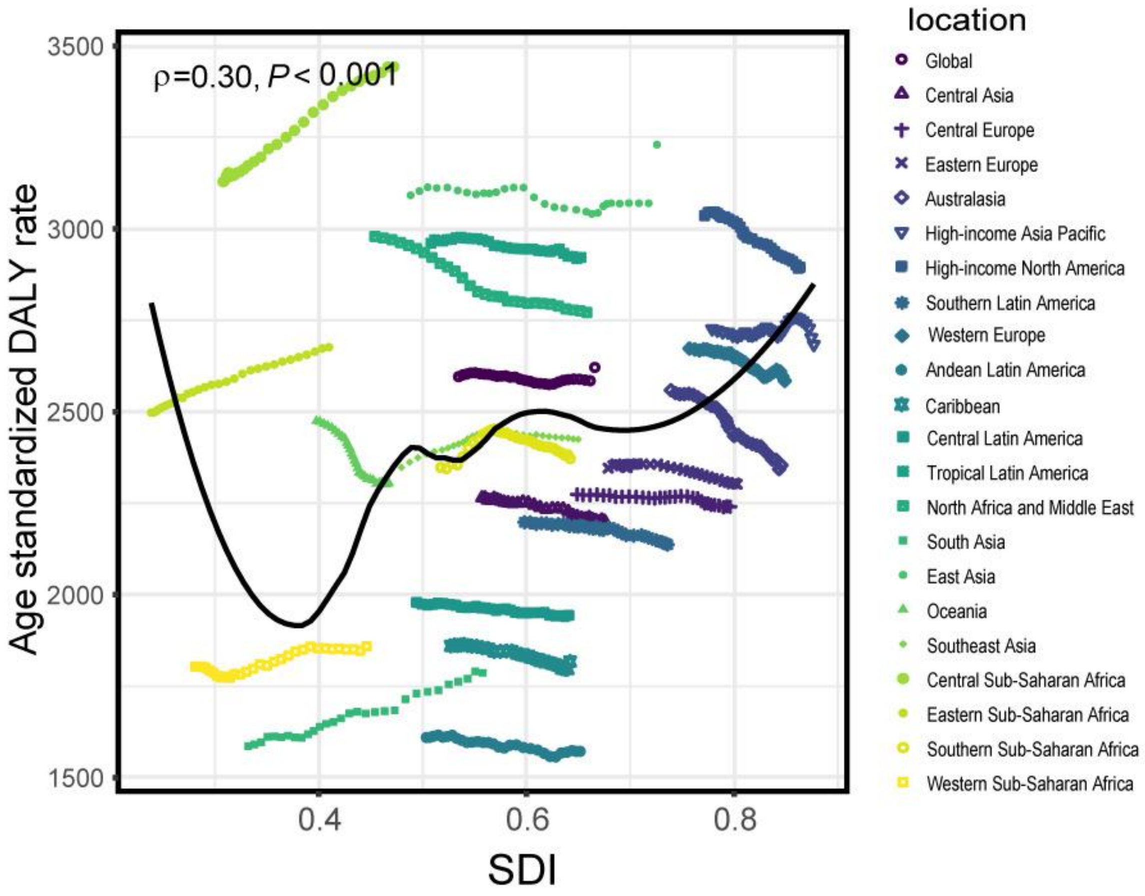

S10 Fig. Spearman correlation between the age-standardized DALY rate due to Alzheimer's disease and other dementias and SDI levels across regions. DALY, disability-adjusted life-year; SDI, socio-demographic index.

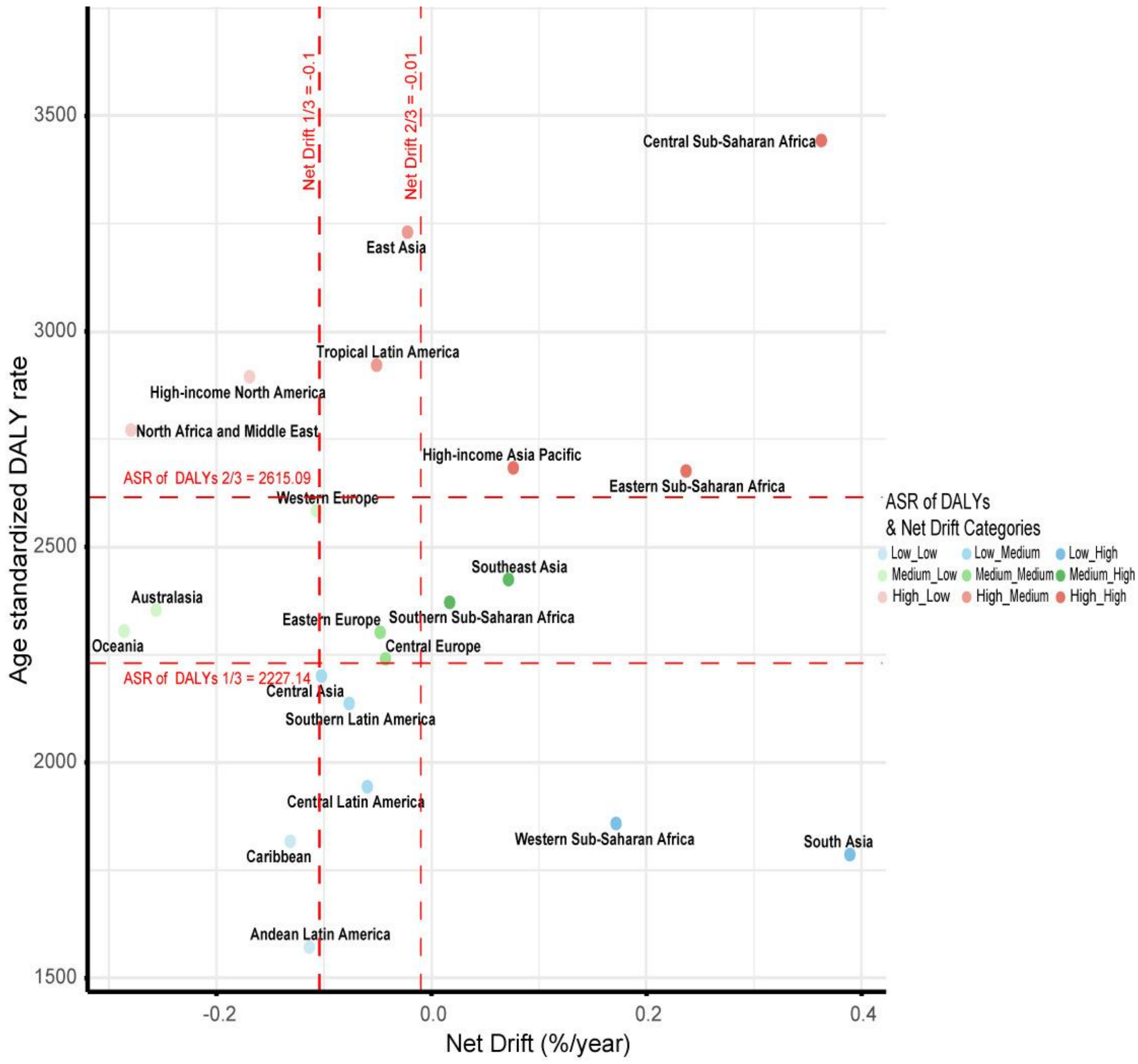

S11 Fig. Classification of 21 regions in 2021 based on ASR of DALYs and net drift. ASR, age-standardized rate; DALYs, disability-adjusted life-years.

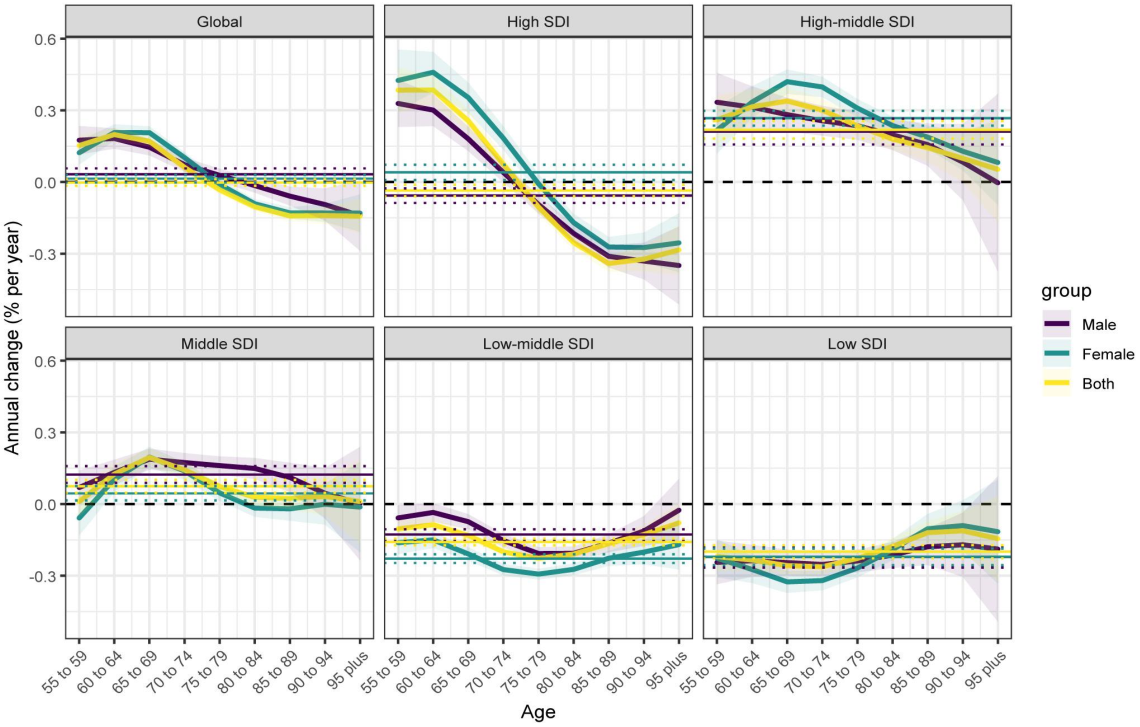

S12 Fig. Local drift of ADOD incidence across nine age groups for both sexes, males, and females (1992–2021). The local drift (i.e., annual percentage change of age-specific incidence, % per year) and its associated 95% CIs are indicated by the dots and shaded areas. ADOD, Alzheimer's disease and other dementias; SDI, socio-demographic index.

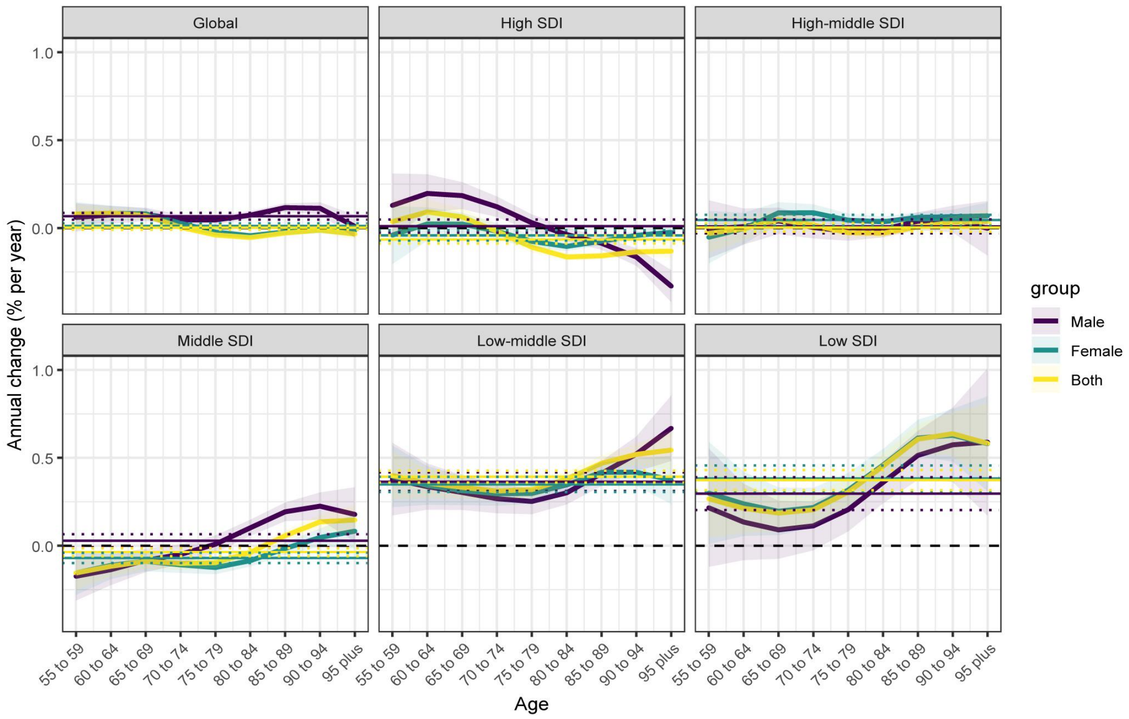

S13 Fig. Local drift of ADOD mortality across nine age groups for both sexes, males, and females (1992–2021). The local drift (i.e., annual percentage change of age-specific incidence, % per year) and its associated 95% CIs are indicated by the dots and shaded areas. ADOD, Alzheimer's disease and other dementias; SDI, socio-demographic index.

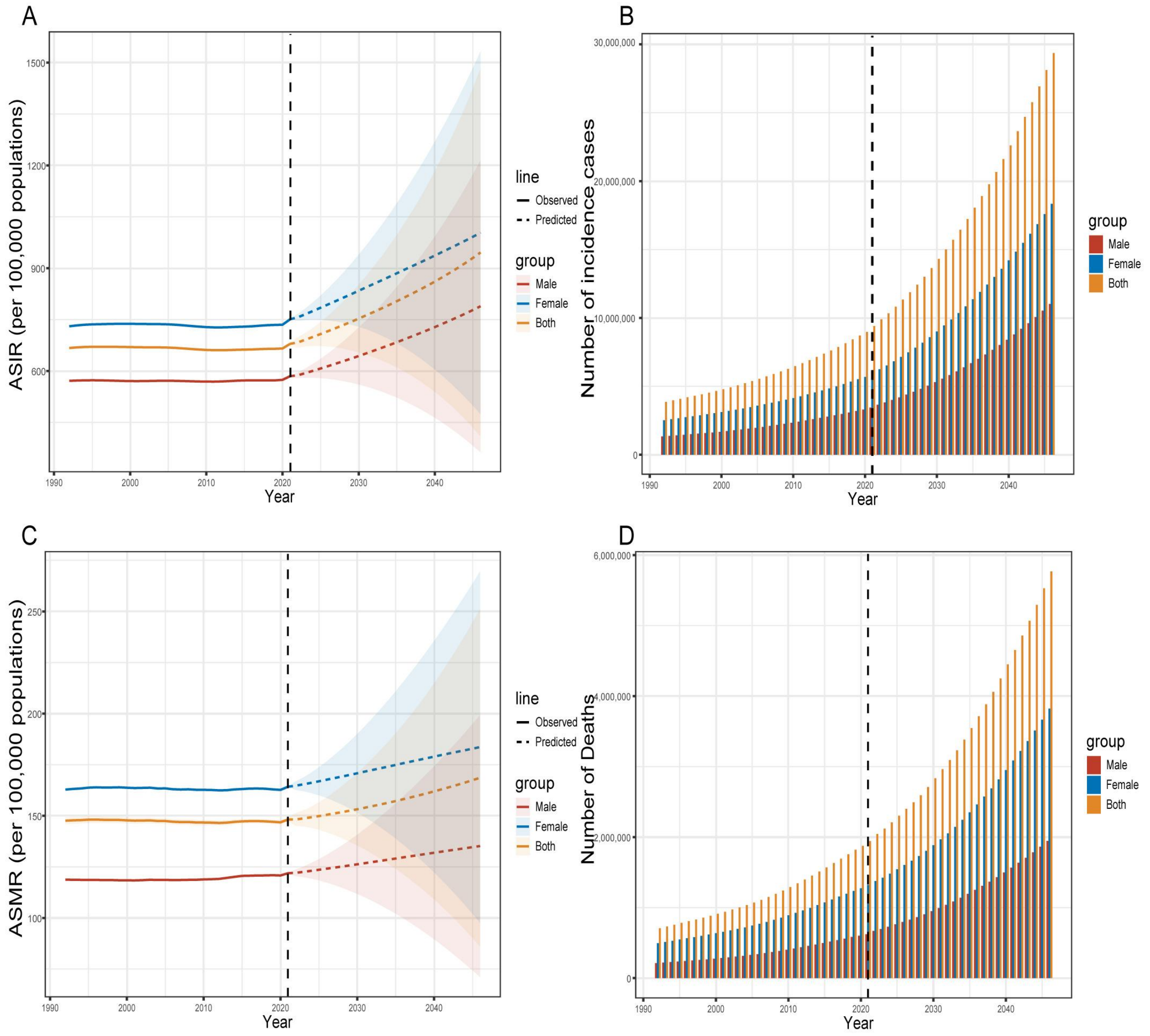

S14 Fig. Future projections of ASIR, ASMR, and the number of ADOD cases (1992–2046). A: Projected ADOD incidence rates (1992–2046) for both sexes combined, males, and females in the population aged 55 years and older; B: Projected incident cases (1992–2046) for both sexes combined, as well as separately for males and females, in the population aged 55 years and older; C: Projected ADOD mortality rates (1992-2046) for both sexes combined, males, and females in the population aged 55 years and older; D: Projected number of deaths (1992–2046) for both sexes combined, as well as separately for males and females, in the population aged 55 years and older. Solid lines represent observed ASIR and ASMR, while dashed lines indicate ASIR and ASMR

projections based on the BAPC model. ASIR, age-standardized incidence rate; ASMR, age-standardized mortality rate; ADOD, Alzheimer's disease and other dementias; BAPC, Bayesian age-period-cohort.

Tables

S1 Table Trends in incidents and ASIR of Alzheimer’ s disease and other dementias from 1992 to 2021. (S1 Table expands on Table 1 by adding data from 21 regions.)

Characteristics	1992		2021		1992 to 2021
	Incident cases, n (95% UI)	ASIR per 100 000, n (95% UI)	Incident cases, n (95% UI)	ASIR per 100 000, n (95% UI)	Net Drift (%/year)
Global	3856613.86 (2680994.62 to 5201269.7)	667.22 (465.83 to 898.17)	9405626.27 (6449244.73 to 12788018.87)	679.44 (466.33 to 922.85)	0 (-0.02 to 0.01)
Sex					
Male	1336053.52 (916524.03 to 1819707.32)	571.41 (394.48 to 775.69)	3447541 (2344534.62 to 4715262.01)	585.06 (398.91 to 798.96)	0.03 (0.01 to 0.06)
Female	2520560.33 (1763882.83 to 3384134.85)	730.59 (512.24 to 980.66)	5958085.27 (4106756.27 to 8087478.4)	751.18 (517.67 to 1019.3)	0.01 (0 to 0.03)
SDI					
High SDI	1448931.49 (1032724.18 to 1915647.28)	720.69 (513.17 to 954.92)	2880559.56 (2013630.61 to 3871095.21)	695.38 (485.15 to 935.17)	-0.04 (-0.06 to -0.01)
High-middle SDI	1002315.73 (689904.02 to 1361906.26)	677.95 (467.98 to 920.24)	2483945.08 (1697255.99 to 3389685.86)	751.7 (513.68 to 1025.46)	0.22 (0.18 to 0.26)

Middle SDI	857671.02 (582973.33 to 1174142.94)	654.3 (446.55 to 893.28)	2744827.26 (1870380.44 to 3766015.17)	701.84 (479.57 to 961.29)	0.07 (0.05 to 0.1)
Low-middle SDI	405028.45 (275250.34 to 554557.47)	538.67 (367.39 to 735.65)	989968.19 (671273.61 to 1356015.02)	523.25 (355.73 to 715.32)	-0.16 (-0.17 to -0.14)
Low SDI	138190.61 (93883.04 to 189775.48)	534.5 (364.81 to 731.06)	298154.99 (202194.73 to 407467.66)	512.69 (349 to 698.09)	-0.2 (-0.23 to -0.17)
Region					
Andean Latin America	14029.75 (9569.33 to 19330.56)	454.39 (310.4 to 625.41)	41957.45 (28640.35 to 57869.69)	447.84 (305.92 to 617.37)	-0.06 (-0.13 to 0.01)
Australasia	28855.89 (20538.96 to 38207.97)	690.34 (490.95 to 916.09)	61628.7 (45135.2 to 80647.09)	597.53 (436.4 to 783.57)	-0.5 (-0.56 to -0.45)
Caribbean	23394.75 (16247.01 to 31717.64)	554.71 (384.72 to 753.51)	50266.39 (34591.5 to 68497.36)	541.92 (373.39 to 737.53)	-0.18 (-0.24 to -0.13)
Central Asia	43219.79 (29378.89 to 59355.32)	632.82 (430.98 to 868.32)	65169.69 (44080.5 to 89416.46)	621.99 (422.1 to 850.3)	-0.05 (-0.09 to 0)
Central Europe	149081.15 (101246.72 to 204544)	650.5 (442.64 to 891.67)	262075.5 (178645.86 to 357459.07)	636.4 (433.25 to 868.77)	-0.08 (-0.1 to -0.05)
Central Latin America	75084.97 (51075.86 to 102888.21)	622.23 (424.02 to 852.29)	236108.11 (160451.02 to 325859.25)	600.91 (408.96 to 827.96)	-0.09 (-0.12 to -0.06)
Central Sub-Saharan Africa	17137.78 (11725.68 to 23631.83)	720.23 (496.15 to 983.77)	39495.29 (27007.3 to 53932.63)	715.38 (492.85 to 969.67)	-0.05 (-0.14 to 0.04)

East Asia	764578.83 (518531.35 to 1049401.56)	712.68 (486.08 to 974.4)	2856983.31 (1959230.01 to 3913193.55)	850.81 (584.72 to 1162.65)	0.37 (0.32 to 0.43)
Eastern Europe	285810.2 (193997.65 to 392319)	665.74 (452.96 to 914.03)	403010.11 (274664.79 to 551823.3)	654.79 (446 to 897.08)	-0.09 (-0.11 to -0.07)
Eastern Sub-Saharan Africa	50622.68 (34689.97 to 69323.41)	600.47 (412.95 to 818.57)	108838.3 (74619.8 to 148374.76)	577.2 (397.33 to 784.2)	-0.13 (-0.17 to -0.08)
High-income Asia Pacifc	220006.29 (152096.76 to 298264.57)	660.08 (456.67 to 895.15)	688720.45 (473439.19 to 934556.81)	673.48 (462.48 to 914.06)	0.27 (0.2 to 0.34)
High-income North America	526323.68 (367134.4 to 710716.28)	787.16 (548.39 to 1064.68)	899694.11 (623330.55 to 1217231.45)	738.59 (511.29 to 998.6)	-0.2 (-0.26 to -0.14)
North Africa and Middle East	167952.29 (115349.6 to 228509.4)	781.83 (538.77 to 1060)	436122.95 (298475.12 to 594965.1)	752.12 (515.99 to 1023.42)	-0.14 (-0.16 to -0.12)
Oceania	1983.4 (1337.17 to 2728.58)	663.37 (451.03 to 907.73)	4861.77 (3271.02 to 6713.67)	634.09 (429.77 to 870.93)	-0.21 (-0.45 to 0.03)
South Asia	305476.1 (206342.09 to 421970.34)	453.44 (307.54 to 623.76)	861226.59 (580108.04 to 1190102.54)	446.19 (301.29 to 614.91)	-0.18 (-0.19 to -0.16)
Southeast Asia	210569.67 (143241.75 to 289035.2)	644.05 (439.69 to 881.2)	533192.88 (361461.19 to 733725.37)	618.4 (420.76 to 848.55)	-0.15 (-0.17 to -0.13)
Southern Latin America	47532.69 (32475.53 to 64915.96)	632.9 (432.43 to 864.15)	94964.71 (64454.05 to 129699.21)	607.88 (412.29 to 830.71)	-0.13 (-0.17 to -0.09)

Southern Sub-Saharan Africa	23897.43 (16326.39 to 32765.31)	631.12 (431.11 to 864.5)	43872.64 (29844.84 to 60339.47)	604.48 (411.58 to 830.75)	-0.13 (-0.19 to -0.07)
Tropical Latin America	93366.68 (64461.94 to 127052.64)	725.88 (501.73 to 986.97)	295720.3 (202582.21 to 405998.65)	715.41 (490.48 to 981.26)	-0.11 (-0.17 to -0.05)
Western Europe	760257.59 (561050.47 to 984581.42)	694.56 (510.49 to 903.63)	1330679.19 (941990.42 to 1774364)	678.98 (478.98 to 908.4)	-0.12 (-0.18 to -0.06)
Western Sub-Saharan Africa	47432.23 (32041.96 to 65452.26)	439.33 (297.84 to 604.56)	91037.81 (61223.4 to 125752.26)	411.17 (277.89 to 565.78)	-0.21 (-0.25 to -0.17)

SDI, socio-demographic index; ASIR, age-standardized incidence rate; 95% UI, 95% Uncertainty Interval; 95% CI, 95% Confidence Interval.

S2 Table Trends in incident cases and ASIR of Alzheimer's disease and other dementias from 1992 to 2021 across countries and territories.

Location	Incident cases 1992, (95%UI)	ASIR 1992, (95%UI)	Incident cases 2021, (95%UI)	ASIR 2021, (95%UI)	Net Drift (%/year)
Afghanistan	6504.83 (4423.89 to 8934.96)	756.15 (516.48 to 1033.86)	7666.31 (5195.99 to 10468.99)	745.27 (505.89 to 1017.8)	-0.05 (-0.2 to 0.1)
Albania	1801.96 (1221.86 to 2502.22)	632.24 (430.02 to 873.87)	4667.12 (3173.03 to 6417.57)	636.79 (433.55 to 875.99)	0.05 (-0.14 to 0.25)
Algeria	12449.28 (8498.52 to 16997.38)	759.53 (520.19 to 1034.31)	34839.59 (23692.65 to 47706.9)	730.52 (497.06 to 1001.51)	-0.13 (-0.3 to 0.03)
American Samoa	16.16 (10.9 to 22.51)	630.79 (428.8 to 872.87)	37.81 (25.48 to 52.49)	617.23 (417.63 to 854.32)	-0.59 (-3.16 to 2.05)
Andorra	68.22 (46.15 to 93.66)	692.99 (469.07 to 951.58)	184.83 (126 to 251.42)	654.77 (446.25 to 888.64)	-0.37 (-1.44 to 0.72)
Angola	2762.41 (1883.21 to 3822)	720.88 (495.65 to 988.58)	8162.62 (5524.54 to 11288.04)	703.81 (480.01 to 966.14)	-0.07 (-0.28 to 0.14)
Antigua and Barbuda	55.7 (38.03 to 76.48)	550.6 (374.89 to 758.08)	83.23 (56.33 to 115.59)	538.51 (365.06 to 746.78)	-0.11 (-1.39 to 1.19)
Argentina	33324.4 (22680.72 to 45865.12)	635.08 (432.32 to 873.07)	60646.15 (41103.56 to 82952.66)	605.88 (410.33 to 829.44)	-0.17 (-0.22 to -0.12)
Armenia	2446.4 (1671.3 to 3363.58)	648.5 (444.36 to 888.39)	4772.22 (3269.76 to 6519.4)	645.61 (441.36 to 882.18)	-0.01 (-0.2 to 0.18)

Australia	23787.43<br>(16980.31 to 31309.87)	683.68<br>(487.48 to 901.93)	50842.99<br>(37507.65 to 65922.77)	581.82<br>(427.91 to 756.15)	-0.55<br>(-0.62 to -0.49)
Austria	15898.63<br>(10877.04 to 21708.38)	708.28<br>(483.48 to 971.04)	24830.32<br>(16865.96 to 33941.16)	665.05<br>(450.87 to 910.33)	-0.25<br>(-0.34 to -0.17)
Azerbaijan	4568.02<br>(3118.83 to 6224.74)	644.19<br>(440.75 to 878.21)	7719.02<br>(5205.76 to 10690.55)	624.06<br>(422.72 to 859.31)	-0.13<br>(-0.26 to 0.01)
Bahamas	127.86<br>(86.85 to 176.61)	550.26<br>(374.29 to 759.27)	305.94<br>(206.47 to 423.89)	539.93<br>(365.89 to 745.37)	-0.04<br>(-0.77 to 0.69)
Bahrain	126.54<br>(85.62 to 174.63)	757.15<br>(517.17 to 1036.74)	586.38<br>(396.2 to 812.37)	743.3<br>(504.64 to 1018.55)	0.36<br>(-1.56 to 2.32)
Bangladesh	28743.72<br>(19497.99 to 39508.7)	465.96<br>(316.12 to 639.84)	86821.03<br>(58609.03 to 119538.27)	449.85<br>(304.06 to 617.97)	-0.11<br>(-0.16 to -0.06)
Barbados	321.04<br>(220.13 to 437.82)	557.37<br>(380.84 to 764.7)	481.04<br>(326.85 to 662.86)	535.34<br>(363.51 to 737.56)	-0.16<br>(-0.72 to 0.39)
Belarus	13932.51<br>(9507.11 to 19160.68)	657.29<br>(449.23 to 903.59)	18541.29<br>(12626.25 to 25452.08)	660.09<br>(449.46 to 906.09)	0.03<br>(-0.05 to 0.11)
Belgium	22545.09<br>(15786.25 to 30402.11)	781.22<br>(546.07 to 1056.31)	33661.96<br>(22816.55 to 45968.65)	695.67<br>(470 to 953.13)	-0.42<br>(-0.49 to -0.35)
Belize	90.97<br>(62.01 to 125.47)	568.27<br>(387.91 to 783.02)	227.31<br>(154.3 to 313.32)	550.35<br>(374.86 to 756.59)	-0.06<br>(-0.92 to 0.8)

Benin	1275.99<br>(867.25 to 1747.77)	460.32<br>(313.83 to 628.41)	2610.9<br>(1790.14 to 3578.94)	425.74<br>(293.22 to 581.63)	-0.21<br>(-0.46 to 0.04)
Bermuda	55.75<br>(37.68 to 76.98)	564.95<br>(381.84 to 780.65)	146.18<br>(99.09 to 201.44)	556.43<br>(377.35 to 767.49)	-0.05<br>(-1.22 to 1.13)
Bhutan	125.37<br>(84.36 to 174.52)	470.53<br>(319.21 to 650.73)	391<br>(263.94 to 541.08)	438.06<br>(295.9 to 605.72)	-0.28<br>(-1.04 to 0.49)
Bolivia (Plurinational State of)	1963.4<br>(1328.74 to 2722.44)	465.47<br>(315.96 to 643.7)	5633.47<br>(3802.08 to 7815.38)	462.55<br>(313.11 to 640.22)	0.01<br>(-0.19 to 0.21)
Bosnia and Herzegovina	3506.94<br>(2372.36 to 4852.59)	632.56<br>(429.59 to 871.13)	6927.23<br>(4713.63 to 9524.11)	632.96<br>(430.54 to 871.25)	0.02<br>(-0.13 to 0.17)
Botswana	388.28<br>(263.75 to 533.66)	609.21<br>(415.71 to 834.12)	963.83<br>(651.16 to 1324.85)	587.78<br>(398.54 to 804.72)	-0.05<br>(-0.61 to 0.52)
Brazil	90889.79<br>(62774.03 to 123675.73)	726.95<br>(502.61 to 988.39)	289930.53<br>(198651.11 to 397971.85)	716.79<br>(491.5 to 982.98)	-0.11<br>(-0.17 to -0.05)
Brunei Darussalam	73.73<br>(49.88 to 101.75)	576.63<br>(391.65 to 794.96)	214.92<br>(145.2 to 297.46)	576.24<br>(391.94 to 792.25)	0.14<br>(-1.84 to 2.17)
Bulgaria	11872.16<br>(8011.24 to 16385.57)	652.24<br>(442.04 to 899.66)	16840.28<br>(11464.47 to 22994.35)	637.2<br>(432.59 to 873.18)	-0.09<br>(-0.27 to 0.09)
Burkina Faso	2495.66<br>(1680.08 to 3431.86)	478.74<br>(324.09 to 654.92)	4914.83<br>(3284.46 to 6842.76)	449.77<br>(301.9 to 623.49)	-0.24<br>(-0.44 to -0.05)

Burundi	1725.09<br>(1171.51 to 2366.96)	611.52<br>(417.93 to 838.45)	2903.35<br>(1954.65 to 4028.76)	567.12<br>(384.75 to 783.02)	-0.22<br>(-0.49 to 0.05)
Cabo Verde	202.97<br>(137.47 to 278.26)	461.71<br>(312.23 to 633.95)	300.09<br>(202.89 to 416.1)	440.53<br>(298.44 to 609.46)	-0.22<br>(-0.86 to 0.43)
Cambodia	3444.26<br>(2338.77 to 4769.57)	656.38<br>(447.51 to 903.86)	9350.62<br>(6308.26 to 12956.62)	631.69<br>(427.75 to 872.43)	-0.18<br>(-0.34 to -0.01)
Cameroon	2331.11<br>(1583.63 to 3232.85)	447.31<br>(305.28 to 617.51)	5752.73<br>(3853.14 to 7991.5)	425.33<br>(286.39 to 588.33)	-0.19<br>(-0.38 to -0.01)
Canada	51443.62<br>(38155.7 to 65934.62)	873.19<br>(646.65 to 1120.94)	102049.58<br>(74171.34 to 133131.97)	741.06<br>(538.16 to 967.24)	-0.6<br>(-0.68 to -0.53)
Central African Republic	832.87<br>(571.71 to 1138.13)	762.62<br>(528.95 to 1034.01)	1425.06<br>(961.1 to 1958.32)	743.87<br>(508.75 to 1008.94)	-0.15<br>(-0.6 to 0.3)
Chad	1799.67<br>(1220.29 to 2483.94)	470.98<br>(319.76 to 649.43)	2730.75<br>(1826.04 to 3794.34)	430.37<br>(289.4 to 596.48)	-0.33<br>(-0.56 to -0.11)
Chile	9718.05<br>(6784.91 to 13084.73)	621.34<br>(433.6 to 837.96)	27468.44<br>(18733.11 to 37583.95)	612.08<br>(417.17 to 837.53)	0.02<br>(-0.07 to 0.1)
China	741695.88<br>(502510.03 to 1018684.51)	718.75<br>(489.67 to 983.61)	2785360.25<br>(1909033.34 to 3818096.16)	861.46<br>(591.65 to 1178.24)	0.38<br>(0.32 to 0.44)
Colombia	16450.62<br>(11172.04 to 22650.64)	642.7<br>(437.89 to 883.17)	61148.25<br>(41801.42 to 84336.8)	635.77<br>(435.82 to 874.8)	-0.04<br>(-0.1 to 0.02)

Comoros	134.09 (90.98 to 185.57)	591.05 (403.36 to 812.6)	349.59 (236.02 to 479.85)	569.88 (385.8 to 780.23)	-0.1 (-0.96 to 0.77)
Congo	835.91 (567.23 to 1156.76)	703.61 (480.59 to 967.61)	1838.18 (1351.78 to 2375.64)	684.14 (506.06 to 882.28)	-0.02 (-0.63 to 0.6)
Cook Islands	10.45 (7.06 to 14.56)	631.97 (428.35 to 877.23)	26.93 (18.16 to 37.22)	619.17 (417.58 to 855.93)	-0.48 (-3.27 to 2.39)
Costa Rica	1870.17 (1273.68 to 2575.02)	640.7 (436.52 to 881.84)	5897.53 (3970.89 to 8173.68)	626.98 (422.95 to 866.9)	-0.08 (-0.26 to 0.1)
Coted'Ivoire	1791.73 (1200.51 to 2492.6)	448.56 (303.74 to 620.23)	4937.48 (3290.37 to 6870.59)	432.09 (289.71 to 597.97)	-0.13 (-0.35 to 0.08)
Croatia	5867.22 (3975.64 to 8061.45)	655.87 (445.23 to 899.59)	11029.6 (7501.54 to 15172.21)	637.73 (432.37 to 879.92)	-0.06 (-0.18 to 0.07)
Cuba	9292.97 (6536.72 to 12529.98)	527.08 (369.62 to 713.17)	18609.74 (12883.53 to 25270.71)	518.05 (359.04 to 703.1)	-0.06 (-0.18 to 0.07)
Cyprus	845.28 (573.36 to 1160.31)	696.06 (474.25 to 951)	2395.37 (1607.79 to 3290.41)	672.18 (450.76 to 926.25)	-0.2 (-0.83 to 0.44)
Czechia	14608.6 (9944.74 to 20091.57)	636.34 (433.42 to 876.15)	25607.43 (17353.24 to 35154.81)	629.77 (426.24 to 865.61)	-0.06 (-0.14 to 0.02)
Democratic People's Republic of Korea	12102.9 (8196.43 to 16703.55)	630.96 (430.28 to 867.01)	29765.02 (20070.4 to 41094.56)	616.99 (417.36 to 851.38)	-0.11 (-0.2 to -0.03)

Democratic Republic of the Congo	12000.21<br>(8179.94 to 16538.05)	718.22<br>(493.53 to 979.55)	26848.46<br>(18231.39 to 36880.66)	720.35<br>(492.97 to 982.99)	-0.02<br>(-0.13 to 0.08)
Denmark	9188.7<br>(6498.09 to 12257.07)	583.01<br>(410.48 to 780.89)	11222.04<br>(7724.93 to 15292.98)	479.58<br>(329.11 to 655.34)	-0.77<br>(-0.88 to -0.66)
Djibouti	81.15<br>(55.02 to 112.04)	614.63<br>(421.44 to 842.71)	348.07<br>(235.65 to 482.79)	587.14<br>(401.19 to 808.56)	-0.21<br>(-1.21 to 0.79)
Dominica	56.85<br>(38.63 to 77.61)	562.93<br>(381.86 to 771.01)	70.45<br>(47.4 to 96.97)	547.32<br>(368.72 to 752.68)	-0.14<br>(-1.46 to 1.19)
Dominican Republic	3158.59<br>(2179.39 to 4289.41)	566.8<br>(390.96 to 771.3)	9036.64<br>(6262.16 to 12282.67)	565.25<br>(391.67 to 767.99)	-0.14<br>(-0.29 to 0)
Ecuador	3735.02<br>(2538.13 to 5151.01)	464.39<br>(315.67 to 639.94)	11846.32<br>(7974.57 to 16406.57)	457.3<br>(307.91 to 633.65)	-0.02<br>(-0.15 to 0.12)
Egypt	21383.51<br>(15078.31 to 28691.93)	731.1<br>(515.41 to 982.28)	46780.07<br>(32158.91 to 63553.24)	720.9<br>(496.67 to 975.34)	-0.04<br>(-0.11 to 0.04)
El Salvador	3135.38<br>(2129.64 to 4319.5)	628.04<br>(427.35 to 863.47)	7336.76<br>(4953.02 to 10124.42)	636.54<br>(430.13 to 877.54)	0.08<br>(-0.07 to 0.22)
Equatorial Guinea	152.72<br>(104.55 to 209.58)	732.29<br>(505.05 to 997.7)	386.69<br>(262.32 to 531.4)	705.02<br>(481.01 to 964.47)	-0.13<br>(-1.02 to 0.78)
Eritrea	523.24<br>(352.23 to 723.99)	622.37<br>(423.67 to 849.21)	1516.93<br>(1014.96 to 2111.04)	589.18<br>(398.78 to 815.62)	-0.17<br>(-0.74 to 0.41)

Estonia	2178.43<br>(1476.43 to 2996.09)	645.05<br>(437.66 to 888.16)	3584.93<br>(2439.14 to 4904.94)	639.32<br>(433.43 to 877.84)	0<br>(-0.22 to 0.22)
Eswatini	191.46<br>(129.05 to 264.16)	590.24<br>(399.99 to 811.1)	323.39<br>(217.41 to 448.7)	571.71<br>(387.06 to 790.03)	-0.11<br>(-0.9 to 0.68)
Ethiopia	12165.68<br>(8258.55 to 16747.75)	614.71<br>(419.2 to 841.78)	30925.74<br>(21038.89 to 42537.26)	577.7<br>(393.35 to 792.98)	-0.17<br>(-0.3 to -0.05)
Fiji	236.92<br>(158.83 to 329.58)	638.5<br>(431.66 to 883.74)	536.61<br>(358.55 to 747.66)	626.45<br>(422.79 to 866.88)	-0.07<br>(-0.76 to 0.62)
Finland	9138.96<br>(6346.59 to 12334.34)	692.41<br>(479.84 to 937.33)	16434.86<br>(11182.49 to 22627.46)	620.39<br>(420.91 to 856.11)	-0.35<br>(-0.45 to -0.24)
France	92242.58<br>(71545.72 to 114637.46)	565.41<br>(435.85 to 706.59)	160511.54<br>(117955.65 to 208688.27)	535.03<br>(391 to 697.24)	-0.2<br>(-0.23 to -0.16)
Gabon	553.66<br>(377.95 to 758.23)	712.22<br>(487.86 to 972.19)	834.28<br>(568.45 to 1145.31)	696.87<br>(476.38 to 951.49)	-0.13<br>(-0.57 to 0.32)
Gambia	196.25<br>(132.72 to 271.69)	472.45<br>(321.07 to 651.94)	522.78<br>(349.66 to 726.02)	439.41<br>(294.77 to 609.64)	-0.28<br>(-0.94 to 0.38)
Georgia	6320.06<br>(4294.03 to 8664.78)	645.04<br>(438.68 to 883.57)	7371.94<br>(5058.41 to 10116.58)	646.21<br>(444.13 to 885.79)	0.02<br>(-0.1 to 0.15)
Germany	199435.13<br>(151073.9 to 252261.27)	833.16<br>(627.6 to 1060.49)	335019.57<br>(242829.59 to 440106.73)	814.9<br>(586.69 to 1076.26)	0.06<br>(-0.13 to 0.24)

Ghana	3065.12<br>(2062.65 to 4250.54)	432.16<br>(292.42 to 597.03)	7743.88<br>(5178.55 to 10733.76)	423.56<br>(284.52 to 585.41)	-0.05<br>(-0.22 to 0.11)
Greece	19132.39<br>(12980.82 to 26084.34)	710.7<br>(482.27 to 971.25)	36538.26<br>(25107.87 to 49960.74)	682.97<br>(466.85 to 939.21)	-0.13<br>(-0.2 to -0.05)
Greenland	27.93<br>(18.93 to 38.56)	788.92<br>(538.95 to 1082.88)	64.98<br>(44.13 to 90.12)	765.61<br>(522.37 to 1055.07)	0.19<br>(-2.04 to 2.47)
Grenada	80.43<br>(54.75 to 110.61)	564.47<br>(383.57 to 777.73)	86.2<br>(58.34 to 119.74)	555.97<br>(377.49 to 768.67)	-0.09<br>(-1.2 to 1.03)
Guam	54.42<br>(36.53 to 75.78)	619.89<br>(419.12 to 857.44)	232.73<br>(157.17 to 323.15)	616.32<br>(417.83 to 851.24)	0.26<br>(-1.57 to 2.12)
Guatemala	2692.28<br>(1826.98 to 3703.33)	643.23<br>(438.3 to 880.08)	10346.07<br>(7052.19 to 14198.28)	638.13<br>(434.93 to 876.27)	-0.02<br>(-0.23 to 0.19)
Guinea	2157.58<br>(1452.62 to 2973.43)	468.2<br>(315.59 to 643.72)	3093.41<br>(2094.46 to 4273.94)	441.65<br>(300.03 to 609.22)	-0.23<br>(-0.43 to -0.03)
Guinea-Bissau	182.91<br>(122.8 to 254.16)	444.08<br>(299.88 to 613.31)	288.74<br>(192.56 to 402.23)	434.88<br>(293.15 to 602.11)	-0.04<br>(-0.9 to 0.84)
Guyana	271.77<br>(185.39 to 374.6)	551.37<br>(376.65 to 758.08)	449.81<br>(304.24 to 620.98)	545.42<br>(370.56 to 749.75)	-0.05<br>(-0.62 to 0.52)
Haiti	2141.15<br>(1450.65 to 2954.37)	576.16<br>(392.9 to 789.99)	4252.73<br>(2875.44 to 5901.83)	537.15<br>(365.36 to 741.8)	-0.26<br>(-0.49 to -0.03)

Honduras	1896.36<br>(1292.85 to 2599.75)	650.94<br>(444.22 to 891.59)	5300.45<br>(3597.55 to 7282.91)	641.86<br>(436.48 to 881.72)	-0.05<br>(-0.26 to 0.15)
Hungary	15265.04<br>(10421.62 to 20954.83)	636.25<br>(434.71 to 874.23)	23665.51<br>(16157.03 to 32575.33)	631.48<br>(430.72 to 869.81)	-0.02<br>(-0.11 to 0.06)
Iceland	419.77<br>(302.1 to 550.62)	770.58<br>(553.75 to 1012.04)	783.56<br>(566.25 to 1036.89)	701.79<br>(507.66 to 925.41)	-0.35<br>(-0.82 to 0.12)
India	234736.36<br>(158287.98 to 324855.86)	447.01<br>(302.87 to 615.56)	698848.94<br>(470921.52 to 968036.67)	445.64<br>(300.93 to 615.47)	-0.15<br>(-0.18 to -0.13)
Indonesia	75873.23<br>(51541.99 to 104801.33)	651.35<br>(443.62 to 897.38)	170381.09<br>(115027.74 to 236531.24)	636.37<br>(431.27 to 878.83)	-0.09<br>(-0.12 to -0.05)
Iran (Islamic Republic of)	23294.01<br>(15936.59 to 32121.6)	777.64<br>(533.89 to 1062.16)	82339.18<br>(56304.92 to 112711.87)	758.4<br>(518.88 to 1036.72)	-0.06<br>(-0.12 to 0)
Iraq	9113.67<br>(6235.48 to 12468.12)	770.18<br>(527.75 to 1051.49)	20338.67<br>(13817.56 to 27946.96)	737.62<br>(503.16 to 1010.44)	-0.18<br>(-0.27 to -0.1)
Ireland	5060.86<br>(3422.68 to 6902.21)	701.34<br>(474.56 to 959.51)	9357.5<br>(6349.98 to 12904.36)	642.4<br>(435.3 to 886.78)	-0.31<br>(-0.45 to -0.16)
Israel	6014.96<br>(4056.97 to 8211.48)	684.53<br>(462.09 to 936.9)	15043.65<br>(10243.67 to 20607.89)	647.53<br>(440.12 to 887.3)	-0.21<br>(-0.34 to -0.08)
Italy	108185.05<br>(75720.44 to 145202.58)	656.26<br>(457.32 to 885.16)	244842.32<br>(166197.66 to 334654.38)	771.16<br>(521.21 to 1058.6)	0.18<br>(-0.02 to 0.37)

Jamaica	2107.5 (1427.06 to 2896.25)	624.31 (422.39 to 859.17)	3351.2 (2260.26 to 4619.48)	593.11 (401.53 to 815.74)	-0.22 (-0.42 to -0.02)
Japan	191488.74 (131466.02 to 260876.34)	653.41 (448.96 to 890.56)	567743.19 (388954.41 to 775468.97)	665.55 (455.09 to 910.32)	0.29 (0.22 to 0.36)
Jordan	1182.12 (801.76 to 1628.18)	751.48 (512.02 to 1030.46)	6607.26 (4519.1 to 9073.25)	765.09 (525.52 to 1045.29)	0.11 (-0.12 to 0.35)
Kazakhstan	11260.23 (7708.39 to 15519.91)	644.28 (442.03 to 886.56)	14482.65 (9811.2 to 19891.92)	629.47 (427.95 to 861.25)	-0.07 (-0.16 to 0.03)
Kenya	6409.28 (4369.94 to 8790.76)	602.67 (411.11 to 825.38)	14783.7 (10050.44 to 20354.2)	592.31 (403.4 to 811.68)	-0.09 (-0.2 to 0.02)
Kiribati	26.64 (18.01 to 36.87)	690.05 (469.85 to 947.82)	49.25 (33.12 to 68.27)	691.93 (470.52 to 951.42)	-0.34 (-2.79 to 2.18)
Kuwait	541.42 (369.65 to 743.89)	777.18 (533.07 to 1061.65)	2469.06 (1685.44 to 3391.54)	747.5 (513.55 to 1022.92)	-0.14 (-0.46 to 0.18)
Kyrgyzstan	2820.78 (1928.04 to 3856.05)	638.72 (437.13 to 872.71)	3837.09 (2611.99 to 5252.99)	645.35 (441.4 to 880.02)	0.08 (-0.11 to 0.27)
Lao People's Democratic Republic	1513.85 (1014.64 to 2095.71)	640.61 (432.2 to 880.51)	3416.55 (2306.96 to 4707.45)	629.54 (427.35 to 864.89)	-0.04 (-0.33 to 0.24)
Latvia	3908.83 (2644.55 to 5350.1)	647.41 (437.88 to 887.08)	5351.89 (3677.74 to 7333.03)	652.05 (446.46 to 896.4)	0.06 (-0.1 to 0.23)

Lebanon	2427.7<br>(1664.45 to 3329.54)	799.09<br>(549.61 to 1090.97)	8860.68<br>(6077.17 to 12049.6)	799.06<br>(547.55 to 1088.28)	0<br>(-0.17 to 0.17)
Lesotho	718.92<br>(486.85 to 987.83)	622.16<br>(422.58 to 854.55)	731.31<br>(495.77 to 1009.8)	617.25<br>(420.93 to 850.78)	-0.02<br>(-0.41 to 0.37)
Liberia	647.56<br>(435.36 to 899.21)	437.13<br>(294.54 to 605.33)	966.14<br>(648.58 to 1335.51)	423.29<br>(285.86 to 582.18)	-0.14<br>(-0.53 to 0.26)
Libya	2133.69<br>(1465.32 to 2921.48)	774.36<br>(533.67 to 1057.49)	4837.81<br>(3281.31 to 6634.12)	742.45<br>(505.03 to 1016.2)	-0.14<br>(-0.32 to 0.03)
Lithuania	4886.58<br>(3331.83 to 6686.83)	632.48<br>(431.24 to 866.74)	7600.04<br>(5170.3 to 10393.68)	639.02<br>(433.08 to 877.69)	0.05<br>(-0.09 to 0.19)
Luxembourg	563.76<br>(383.69 to 769.23)	578.73<br>(393.22 to 792.6)	1006.7<br>(693.93 to 1378.12)	499.52<br>(343.65 to 684.35)	-0.49<br>(-0.91 to -0.07)
Madagascar	3504.6<br>(2399.75 to 4796.96)	590.34<br>(405.86 to 805.77)	5891.61<br>(3972.41 to 8186.27)	563.42<br>(383.75 to 776.7)	-0.16<br>(-0.32 to 0.01)
Malawi	2583.6<br>(1749.23 to 3575.33)	595.33<br>(404.78 to 819.09)	4768.74<br>(3218.2 to 6593.72)	591.78<br>(401.44 to 813.74)	-0.01<br>(-0.22 to 0.2)
Malaysia	8756.85<br>(5936.44 to 12095.15)	653.75<br>(444.4 to 900.97)	24270.49<br>(16218.57 to 33761.31)	625.44<br>(419.3 to 867.66)	-0.13<br>(-0.21 to -0.04)
Maldives	60.49<br>(40.68 to 83.77)	616.55<br>(417.15 to 848.33)	278.44<br>(189.14 to 384.58)	636.84<br>(433.15 to 878.82)	0.39<br>(-1.44 to 2.24)

Mali	1964.8<br>(1324.17 to 2713.57)	459.42<br>(311.25 to 631.03)	4239.9<br>(2863.6 to 5872.5)	442.43<br>(300.21 to 609.74)	-0.13<br>(-0.35 to 0.09)
Malta	498.69<br>(339.04 to 684.74)	700.19<br>(476.16 to 961.63)	1277.42<br>(867.5 to 1750.12)	653.85<br>(443.27 to 897.3)	-0.2<br>(-0.61 to 0.21)
Marshall Islands	10.83<br>(7.25 to 15.08)	607.02<br>(409.84 to 841.14)	18.08<br>(12.07 to 25.32)	587.81<br>(396.37 to 814.38)	-0.52<br>(-4.3 to 3.41)
Mauritania	607.37<br>(412.06 to 839.53)	468.71<br>(318.56 to 646.21)	1214.34<br>(814.47 to 1685.77)	436.96<br>(294.05 to 605.77)	-0.25<br>(-0.62 to 0.13)
Mauritius	611.46<br>(411.48 to 846.45)	631.05<br>(426.19 to 870.42)	1792.44<br>(1204.54 to 2481.07)	629.1<br>(423.72 to 867.74)	0.04<br>(-0.3 to 0.38)
Mexico	35372.03<br>(24029.26 to 48587.56)	583.45<br>(396.31 to 802.44)	104119.93<br>(70624.31 to 144348.89)	549.37<br>(372.9 to 760.93)	-0.14<br>(-0.19 to -0.1)
Micronesia (Federated States of)	40.62<br>(27.57 to 55.92)	677.92<br>(462.32 to 929.74)	51.34<br>(34.53 to 71.3)	684.72<br>(464.61 to 943.04)	0.44<br>(-1.78 to 2.71)
Monaco	106.75<br>(72.68 to 145.67)	702.45<br>(476.63 to 962.99)	133.8<br>(90.58 to 184.52)	651.23<br>(440.3 to 899.34)	-0.25<br>(-1.31 to 0.82)
Mongolia	889.9<br>(609.15 to 1217.49)	648.64<br>(445.69 to 885.87)	1651.01<br>(1120.89 to 2264.75)	657.2<br>(448.63 to 899.2)	0.09<br>(-0.25 to 0.43)
Montenegro	650.12<br>(440.83 to 892.87)	651.51<br>(442.31 to 894.01)	968.94<br>(658.41 to 1332.95)	632.38<br>(429.96 to 869.48)	-0.15<br>(-0.5 to 0.2)

Morocco	16146.38<br>(11098.8 to 22154.67)	773.94<br>(532.88 to 1060.21)	34518.14<br>(23553.25 to 47475.53)	726.93<br>(497.12 to 997.75)	-0.23<br>(-0.31 to -0.16)
Mozambique	4099.19<br>(2801.06 to 5647.11)	606.65<br>(416.34 to 832.07)	6726.63<br>(4574.36 to 9216.3)	589.78<br>(403.49 to 803.77)	-0.09<br>(-0.25 to 0.07)
Myanmar	19094.2<br>(12928.18 to 26242.64)	684<br>(465.72 to 934.44)	40746.4<br>(27371.03 to 56287.33)	636.65<br>(429.05 to 876.65)	-0.27<br>(-0.34 to -0.19)
Namibia	449.68<br>(306.2 to 624.61)	604.02<br>(413.18 to 833.73)	924<br>(626.26 to 1279.47)	585.08<br>(398 to 807.58)	-0.09<br>(-0.58 to 0.41)
Nauru	2.76<br>(1.85 to 3.84)	633.35<br>(427.97 to 874.08)	3.74<br>(2.52 to 5.21)	645.8<br>(439.33 to 893.12)	NA
Nepal	5506.66<br>(3729.32 to 7610.77)	515.49<br>(350.66 to 707.78)	13906.39<br>(9390.04 to 19236.65)	460.18<br>(311.74 to 635.26)	-0.48<br>(-0.6 to -0.36)
Netherlands	27153.84<br>(20015.74 to 34818.21)	728.09<br>(534.95 to 936.85)	47435.77<br>(33556.47 to 63124.6)	698.64<br>(493.01 to 931.62)	0.02<br>(-0.12 to 0.15)
New Zealand	5068.46<br>(3433.97 to 6963.98)	723.4<br>(490.29 to 994.86)	10785.71<br>(7270.69 to 14791.11)	684.99<br>(461.38 to 940.13)	-0.23<br>(-0.36 to -0.1)
Nicaragua	1501.98<br>(1019.31 to 2066.99)	651.12<br>(442.78 to 894.66)	4602.2<br>(3124.25 to 6311.69)	650.31<br>(441.54 to 891.74)	0.02<br>(-0.18 to 0.22)
Niger	1354.1<br>(908.75 to 1889.53)	466.72<br>(315.87 to 647.17)	3871.96<br>(2572.36 to 5409.78)	441.16<br>(295.64 to 612.05)	-0.19<br>(-0.45 to 0.07)

Nigeria	23491.1 (15811.96 to 32517.69)	422.02 (284.69 to 583.09)	39973.99 (26753.39 to 55526.56)	385.3 (259.23 to 532.93)	-0.27 (-0.33 to -0.21)
Niue	2.52 (1.7 to 3.47)	623.22 (420.72 to 861.47)	2.07 (1.39 to 2.88)	606.89 (406.02 to 842.54)	0.02 (-8.88 to 9.79)
North Macedonia	1649.62 (1119.1 to 2287.56)	632.67 (431.16 to 873.12)	2887.15 (1956.08 to 3969.77)	628.96 (427.37 to 863.04)	-0.01 (-0.28 to 0.26)
Northern Mariana Islands	8.99 (6.01 to 12.57)	619.08 (418.38 to 859.32)	34.5 (22.98 to 47.99)	610.3 (408.89 to 842.81)	-0.54 (-4.04 to 3.09)
Norway	10566.06 (7282.86 to 14322.33)	770.17 (529.58 to 1047.05)	12869.48 (8718.74 to 17753.87)	652.83 (441.63 to 900.55)	-0.64 (-0.75 to -0.54)
Oman	601.55 (411.34 to 825.8)	750.02 (513.99 to 1026.38)	1405.42 (951.15 to 1941.44)	707.23 (480.43 to 973.6)	-0.14 (-0.51 to 0.22)
Pakistan	36364 (24556.44 to 50265.07)	475.6 (321.4 to 656.45)	61259.23 (41136.93 to 84599.4)	444.76 (299.53 to 611.75)	-0.26 (-0.31 to -0.21)
Palau	7.58 (5.06 to 10.59)	604.11 (405.9 to 840.43)	14.49 (9.62 to 20.3)	587.54 (392.97 to 816.87)	-0.58 (-4.4 to 3.39)
Palestine	928.46 (637.03 to 1265.82)	778.25 (534.45 to 1058.08)	2206.54 (1505.59 to 3033.66)	748.23 (512.91 to 1025.34)	-0.16 (-0.47 to 0.16)
Panama	1521.31 (1036.7 to 2099.62)	624.3 (425.7 to 861.42)	4731.93 (3194.86 to 6552.02)	615.43 (416.08 to 851.49)	-0.06 (-0.26 to 0.14)

Papua New Guinea	1191.01<br>(802.78 to 1644.47)	679.28<br>(462.8 to 931.03)	3093.13<br>(2076.9 to 4283.09)	638.91<br>(432.54 to 878.54)	-0.28<br>(-0.61 to 0.05)
Paraguay	2476.89<br>(1681.52 to 3392.34)	694.88<br>(472.58 to 950.62)	5789.76<br>(3920.5 to 7971.06)	653.47<br>(443.79 to 897.42)	-0.23<br>(-0.4 to -0.07)
Peru	8331.33<br>(5688.9 to 11446.43)	447.87<br>(306.35 to 614.3)	24477.66<br>(16666.88 to 33669.27)	440.4<br>(300.36 to 605.05)	-0.1<br>(-0.19 to -0.02)
Philippines	25244.95<br>(17190.84 to 34653.63)	668.88<br>(456.18 to 918.36)	65982.14<br>(44759.73 to 90930.91)	640.33<br>(435.44 to 879.77)	-0.21<br>(-0.29 to -0.13)
Poland	47394.38<br>(32154.94 to 65051.93)	680.12<br>(461.77 to 933.98)	85985.65<br>(58292.96 to 117882.28)	646.47<br>(437.33 to 886.62)	-0.17<br>(-0.22 to -0.13)
Portugal	16150.67<br>(10948.72 to 22238.79)	679.55<br>(461.58 to 937.13)	35089.79<br>(23896.46 to 47960.35)	669.54<br>(454.37 to 918.11)	-0.02<br>(-0.11 to 0.06)
Puerto Rico	3622.84<br>(2475.93 to 4958.83)	565.24<br>(385.62 to 775.77)	8604.57<br>(5833.02 to 11887.81)	554.14<br>(376.26 to 765.14)	-0.08<br>(-0.24 to 0.07)
Qatar	62.35<br>(42.32 to 86.14)	729.32<br>(498.28 to 998)	471.29<br>(314.91 to 650.45)	720.16<br>(487.41 to 983.53)	0.24<br>(-1.62 to 2.14)
Republic of Korea	26855.03<br>(19044.87 to 35762.06)	718.32<br>(510.97 to 956.07)	113295.64<br>(79159.7 to 152869.7)	707.49<br>(494.15 to 955.09)	0.05<br>(-0.07 to 0.18)
Republic of Moldova	3757.16<br>(2536.31 to 5201.29)	628.65<br>(425.92 to 868.31)	6583.04<br>(4482.11 to 8991.73)	634.76<br>(432.58 to 866.17)	0.03<br>(-0.14 to 0.21)

Romania	26064.02 (17610.74 to 35864.21)	632.06 (428.77 to 868.87)	44776.45 (30617.81 to 61450.87)	630.46 (430.15 to 866.75)	-0.04 (-0.11 to 0.03)
Russian Federation	181784.33 (123455.3 to 249319.77)	665.7 (453.25 to 913.5)	270748.24 (184767 to 371039.45)	655.92 (447.31 to 898.91)	-0.09 (-0.11 to -0.06)
Rwanda	1800.76 (1221.34 to 2479.71)	601.6 (410.51 to 824.53)	4189.93 (2840.53 to 5755.55)	602.26 (411.27 to 824.64)	0.04 (-0.22 to 0.3)
Saint Kitts and Nevis	34.17 (23.22 to 47.3)	534.94 (363.28 to 742.66)	45.16 (30.39 to 62.58)	529.04 (358.64 to 728.65)	0.31 (-1.99 to 2.67)
Saint Lucia	77.13 (52.21 to 106.54)	561.23 (380.26 to 776.42)	212.38 (144.77 to 293.79)	543.76 (370.67 to 752.07)	-0.28 (-1.35 to 0.81)
Saint Vincent and the Grenadines	66.56 (45.44 to 91.22)	572.44 (390.41 to 786.55)	122.02 (82.67 to 168.55)	547.01 (370.91 to 755.95)	-0.24 (-1.41 to 0.94)
Samoa	67.87 (45.55 to 94.19)	631.45 (426.27 to 872.41)	116.41 (77.26 to 161.3)	613.12 (408.74 to 847.39)	-0.09 (-1.23 to 1.07)
San Marino	50.43 (34.7 to 68.71)	707.71 (486.7 to 965.87)	104.14 (70.36 to 143.69)	635.49 (428.68 to 876.52)	-0.4 (-1.71 to 0.93)
Sao Tome and Principe	39.86 (26.85 to 55.12)	440.55 (297.96 to 608.07)	54.39 (36.24 to 75.47)	413.93 (276.58 to 573.28)	-0.33 (-1.83 to 1.19)
Saudi Arabia	5171.27 (3512.34 to 7147.25)	725.39 (494.18 to 1000.46)	10909.07 (7333.68 to 15216.83)	684.78 (463.98 to 944.97)	-0.2 (-0.33 to -0.07)

Senegal	1891.52 (1271.78 to 2614.45)	462.48 (312.63 to 637.84)	4140.36 (2764.72 to 5782.2)	431.77 (289.21 to 600.87)	-0.27 (-0.48 to -0.07)
Serbia	9090.96 (6133.92 to 12592.17)	631.75 (427.9 to 868.39)	19009.06 (12891.6 to 26172.12)	632.09 (428.83 to 870.16)	0.01 (-0.09 to 0.12)
Seychelles	61.54 (41.62 to 85.36)	637.6 (431.36 to 883.95)	100.6 (67.63 to 139.58)	614.88 (414.39 to 851.06)	-0.23 (-1.37 to 0.93)
Sierra Leone	1299.09 (880.82 to 1782.22)	471.06 (319.69 to 646.21)	2002.65 (1350.99 to 2774.87)	446.55 (301.92 to 618.03)	-0.2 (-0.47 to 0.06)
Singapore	1588.79 (1156.64 to 2067.3)	518.02 (379.01 to 672.63)	7466.7 (5494.55 to 9654.47)	540.6 (398.95 to 696.88)	0.16 (-0.03 to 0.36)
Slovakia	6189.76 (4203.66 to 8524.39)	634.15 (431.08 to 873.46)	10299.71 (7028.94 to 14152.83)	627.78 (428.6 to 862.13)	-0.05 (-0.17 to 0.07)
Slovenia	2642.98 (1791.12 to 3636.85)	622.45 (422.08 to 856.83)	5596.77 (3818.22 to 7681.74)	625.43 (425.92 to 860.31)	0.06 (-0.11 to 0.24)
Solomon Islands	86.99 (58.08 to 120.75)	638.77 (430.52 to 878.45)	229.52 (153.92 to 318.01)	644.31 (434.86 to 888.85)	0.36 (-1.49 to 2.25)
Somalia	1289.68 (872.8 to 1780.74)	607.43 (415.07 to 832.08)	3105.24 (2098.17 to 4291.84)	603.62 (411.9 to 828.74)	-0.02 (-0.34 to 0.29)
South Africa	19193.2 (13129.48 to 26299)	638.75 (436.65 to 875.02)	36897.72 (25110.56 to 50819.64)	607.62 (413.66 to 836.56)	-0.15 (-0.22 to -0.09)

South Sudan	1875.06<br>(1270.33 to 2588.6)	589.77<br>(401.58 to 812.55)	2246.99<br>(1519.39 to 3102.25)	558.47<br>(380.21 to 767.52)	-0.15<br>(-0.4 to 0.09)
Spain	64598.23<br>(49120.66 to 81464.91)	640.69<br>(485.92 to 809.94)	121203.8<br>(83916.44 to 164058.9)	584.76<br>(404.39 to 793.71)	-0.31<br>(-0.37 to -0.25)
Sri Lanka	8854.6<br>(6000.51 to 12247.17)	621.99<br>(422.98 to 858.84)	24346.88<br>(16451.97 to 33843.37)	603.26<br>(408.73 to 836.25)	-0.12<br>(-0.22 to -0.01)
Sudan	9662.56<br>(6618.96 to 13182.16)	762<br>(523.63 to 1037.62)	17064.38<br>(11576 to 23448.48)	715.14<br>(487.35 to 980.2)	-0.23<br>(-0.34 to -0.13)
Suriname	226.18<br>(153.83 to 311.7)	594.21<br>(405.24 to 816.05)	550.54<br>(375.72 to 753.99)	568.19<br>(388.69 to 776.94)	-0.19<br>(-0.72 to 0.35)
Sweden	23990.79<br>(17205.51 to 31776.92)	770.03<br>(549.43 to 1025.95)	33010.6<br>(22651.01 to 44967.93)	723.54<br>(495.69 to 987.3)	-0.06<br>(-0.2 to 0.08)
Switzerland	14747.87<br>(10164.4 to 19930.05)	712.82<br>(489.7 to 967.42)	24339.82<br>(16543.38 to 33318.49)	648.7<br>(440.45 to 888.81)	-0.37<br>(-0.45 to -0.28)
Syrian Arab Republic	5282.05<br>(3615.62 to 7250.38)	788.89<br>(542.07 to 1078.6)	12145.69<br>(8251.88 to 16650.79)	737.98<br>(502.94 to 1009.03)	-0.24<br>(-0.37 to -0.11)
Taiwan (Province of China)	10780.05<br>(7468.66 to 14517.63)	514.71<br>(358.28 to 690.07)	41858.04<br>(29545.61 to 55595.71)	557.44<br>(395.01 to 737.48)	0.45<br>(0.35 to 0.54)
Tajikistan	2489.28<br>(1686.94 to 3419.73)	631.15<br>(428.52 to 865.22)	3767.75<br>(2551.88 to 5224.19)	593.19<br>(403.26 to 815.52)	-0.21<br>(-0.4 to -0.02)

Thailand	27177.72<br>(18657.17 to 37119.46)	571.08<br>(392.62 to 777.72)	107287.86<br>(72686.11 to 148620.3)	581.15<br>(394.02 to 804.3)	0.13<br>(0.09 to 0.18)
Timor-Leste	194.12<br>(131.67 to 268.49)	671.55<br>(457.76 to 922.63)	714.9<br>(486.74 to 988.77)	637.2<br>(434.77 to 878.71)	-0.16<br>(-0.76 to 0.44)
Togo	636.38<br>(430.19 to 879.89)	455.22<br>(308.68 to 627.38)	1677.4<br>(1124.07 to 2329.32)	441.71<br>(298.31 to 610.73)	-0.11<br>(-0.47 to 0.25)
Tokelau	1.29<br>(0.87 to 1.78)	630.01<br>(426.65 to 868.37)	1.56<br>(1.05 to 2.17)	618.57<br>(417.58 to 860.29)	NA
Tonga	48.81<br>(33.08 to 67.39)	651.21<br>(443.09 to 895.61)	79.31<br>(53.61 to 109.42)	632.57<br>(427.93 to 872.86)	-0.06<br>(-1.36 to 1.26)
Trinidad and Tobago	745.35<br>(510.02 to 1020.63)	569.95<br>(389.83 to 782.82)	1753.43<br>(1184.61 to 2415.64)	559.23<br>(378.38 to 769.22)	-0.07<br>(-0.44 to 0.3)
Tunisia	5682.38<br>(3885.62 to 7736.36)	805.31<br>(552.38 to 1093.35)	15098.08<br>(10338.94 to 20544.24)	763.62<br>(523.9 to 1037.53)	-0.19<br>(-0.33 to -0.05)
Turkey	40233.19<br>(27525.58 to 54760.89)	837.76<br>(575.43 to 1136.5)	112752.31<br>(76701.48 to 154065.05)	791.47<br>(539.56 to 1079.55)	-0.23<br>(-0.27 to -0.19)
Turkmenistan	1589.4<br>(1081.48 to 2181.81)	637.3<br>(435.44 to 870.74)	3063.45<br>(2067.2 to 4245.41)	606.33<br>(411.32 to 836.39)	-0.19<br>(-0.41 to 0.04)
Tuvalu	4.91<br>(3.31 to 6.81)	655.66<br>(446.09 to 901.02)	8.61<br>(5.78 to 11.92)	642.23<br>(433.26 to 886.26)	NA

Uganda	4613.95<br>(3150.62 to 6339.83)	587.53<br>(402.22 to 805.25)	9690.74<br>(6550.53 to 13401.01)	579.84<br>(393.89 to 798.97)	-0.05<br>(-0.2 to 0.09)
Ukraine	75362.35<br>(50866.69 to 103515.69)	673.95<br>(455.38 to 925.45)	90600.67<br>(61533.77 to 124394.22)	654.4<br>(443.89 to 899.79)	-0.13<br>(-0.17 to -0.09)
United Arab Emirates	271.71<br>(183.26 to 374.98)	701.08<br>(476.62 to 964.11)	1472.53<br>(975.46 to 2086.34)	644.47<br>(434.11 to 895.09)	-0.25<br>(-0.91 to 0.42)
United Kingdom	113031.83<br>(78236.5 to 152815.46)	652.15<br>(450.57 to 885.2)	162210.49<br>(111810.35 to 219665.67)	619.67<br>(425.5 to 841.57)	-0.14<br>(-0.22 to -0.06)
United Republic of Tanzania	7911.04<br>(5456.45 to 10718.64)	598.17<br>(414.48 to 807.88)	17113.31<br>(11971.14 to 22959.37)	555.07<br>(389.54 to 743.84)	-0.25<br>(-0.37 to -0.14)
United States of America	474840.3<br>(328313.11 to 645443.01)	778.62<br>(537.67 to 1060.23)	797565.45<br>(547039.18 to 1088140.23)	738.41<br>(506.13 to 1006.58)	-0.16<br>(-0.22 to -0.09)
United States Virgin Islands	63.41<br>(42.73 to 87.91)	550.1<br>(371.17 to 761.26)	176.77<br>(119.36 to 245.3)	535.51<br>(360.79 to 744.58)	0.23<br>(-1.61 to 2.1)
Uruguay	4487.93<br>(3077.09 to 6135.24)	641.61<br>(439.48 to 878.24)	6844.87<br>(4672.89 to 9376.76)	607.98<br>(414.01 to 834.4)	-0.23<br>(-0.38 to -0.08)
Uzbekistan	10835.7<br>(7356.03 to 14862.78)	604.94<br>(410.55 to 830.14)	18504.55<br>(12416.1 to 25530.76)	600.92<br>(404.94 to 825.07)	-0.01<br>(-0.1 to 0.08)
Vanuatu	39.35<br>(26.16 to 54.95)	609.56<br>(410.33 to 844.85)	107.4<br>(71.6 to 150.12)	595.63<br>(400.74 to 824.62)	0.21<br>(-1.91 to 2.39)

Venezuela (Bolivarian Republic of)	10644.85<br>(7330.85 to 14443.76)	726.99<br>(500.68 to 986.38)	32624.99<br>(22297.09 to 44842.85)	704.99<br>(483.56 to 965.64)	-0.11<br>(-0.19 to -0.04)
Viet Nam	39376.92<br>(26660.29 to 54516.6)	656.31<br>(445.53 to 906.24)	83780.78<br>(56856.22 to 115973.67)	619.7<br>(421.87 to 856.26)	-0.23<br>(-0.28 to -0.19)
Yemen	4660.69<br>(3182.45 to 6391.79)	814.78<br>(558.44 to 1111.72)	12347.73<br>(8413.38 to 16913.9)	757.38<br>(517.19 to 1033.97)	-0.24<br>(-0.39 to -0.1)
Zambia	1868.03<br>(1260.95 to 2569.95)	575.07<br>(390.88 to 788.26)	4182.97<br>(2860.99 to 5773.63)	579.6<br>(398.42 to 796.2)	0.06<br>(-0.18 to 0.3)
Zimbabwe	2955.88<br>(2014.67 to 4098.94)	595.93<br>(407.17 to 822.9)	4032.39<br>(2715.95 to 5587.44)	578.97<br>(392.03 to 798.66)	-0.08<br>(-0.27 to 0.11)

ASIR, age-standardized incidence rate; 95% UI, 95% Uncertainty Intervals; 95% CI, 95% Confidence Interval.

S3 Table Trends in prevalence cases and ASPR of Alzheimer' s disease and other dementias from 1992 to 2021. (S3 Table expands on Table 2 by adding data from 21 regions.)

Characteristics	1992		2021		1992 to 2021
	Prevalence cases, n (95% UI)	ASPR per 100 000, n (95% UI)	Prevalence cases, n (95% UI)	ASPR per 100 000, n (95% UI)	Net Drift (%/year)
Global	22219463.89 (17659952.46 to 27594696.06)	3870.6 (3080.04 to 4799.91)	54905286.05 (43228738.62 to 68594042.73)	3975.78 (3131.76 to 4965.09)	0.01 (0 to 0.02)
Sex					
Male	7688456.83 (6050374.27 to 9613280.24)	3273.84 (2581.81 to 4087.21)	19855926.85 (15474985.23 to 24933535.22)	3367.78 (2626.54 to 4227.63)	0.04 (0.02 to 0.05)
Female	14531007.06 (11592656.12 to 17989874.75)	4247.11 (3389.18 to 5254.13)	35049359.2 (27727089.28 to 43712875.64)	4414.52 (3492.78 to 5504.77)	0.03 (0.02 to 0.04)
SDI					
High SDI	8240297.5 (6643299.65 to 10110877.68)	4135.57 (3331.34 to 5076.53)	16871817.32 (13408805.74 to 20903119.14)	4056.36 (3222.64 to 5026.88)	0 (-0.03 to 0.03)
High-middle SDI	5815572.9 (4590172.26 to 7252114.57)	3961.89 (3130.01 to 4936.95)	14480533.5 (11331175.76 to 18179790.92)	4393.63 (3439 to 5514.2)	0.21 (0.17 to 0.24)
Middle SDI	4998893.34 (3924576.62 to 6271364.49)	3811.7 (2992.43 to 4778.82)	16101011.37 (12596761.11 to 20247236.97)	4145.89 (3245.57 to 5208.98)	0.09 (0.06 to 0.12)

Low-middle SDI	2337611.61 (1834183.61 to 2936370.88)	3089.31 (2424.44 to 3877.49)	5683826.73 (4457243.19 to 7148543.95)	2992.19 (2345.49 to 3763.73)	-0.17 (-0.17 to -0.16)
Low SDI	801287.88 (627826.64 to 1004440.26)	3065.66 (2400.08 to 3840.6)	1720808.15 (1351030.99 to 2157652.95)	2930.09 (2300.71 to 3672.62)	-0.21 (-0.23 to -0.18)
Region					
Andean Latin America	79596.08 (62323.35 to 99602.14)	2563.52 (2006.87 to 3205.63)	236659.56 (185035.54 to 296825.33)	2522.78 (1972.38 to 3163.43)	-0.06 (-0.09 to -0.04)
Australasia	164242.81 (131176.52 to 201515.68)	3972.84 (3171.67 to 4873.37)	355281.12 (287134.83 to 433153.51)	3448.66 (2780.7 to 4210.53)	-0.49 (-0.53 to -0.44)
Caribbean	135225.04 (107329.49 to 167881.19)	3204.69 (2540.25 to 3979.88)	291362.83 (229160.21 to 364207.9)	3149.17 (2478.55 to 3934.3)	-0.18 (-0.2 to -0.16)
Central Asia	250395.41 (196382.07 to 312841.78)	3650.3 (2861.88 to 4559.84)	378314.23 (295805.21 to 474978.94)	3584.41 (2804.31 to 4491.82)	-0.05 (-0.07 to -0.03)
Central Europe	858407.43 (670267.64 to 1079441.12)	3755.46 (2931.76 to 4718.54)	1506794.56 (1178910.6 to 1892419.64)	3665.92 (2868.06 to 4603.31)	-0.08 (-0.09 to -0.07)
Central Latin America	427508.46 (336027.87 to 535961.26)	3533.62 (2776.93 to 4429.32)	1340610.89 (1051581.85 to 1684012.97)	3407.96 (2674.45 to 4278.76)	-0.09 (-0.1 to -0.08)
Central Sub-Saharan Africa	100376.97 (78612.43 to 125741.73)	4318.34 (3388.48 to 5391.59)	233338.6 (184599.98 to 291083.95)	4299.64 (3403.52 to 5344.19)	-0.03 (-0.07 to 0)

East Asia	4440943.48 (3462588.45 to 5600430.41)	4202.99 (3281.61 to 5293.38)	16827718.17 (13084117.69 to 21160623.92)	5106.19 (3979.84 to 6412.27)	0.37 (0.34 to 0.41)
Eastern Europe	1642127.82 (1278958.96 to 2066681.69)	3835.53 (2986.52 to 4830.99)	2320690.61 (1808624.46 to 2925564.2)	3762.55 (2932.28 to 4741.67)	-0.09 (-0.1 to -0.08)
Eastern Sub-Saharan Africa	298065.01 (234635.91 to 372513.3)	3506.55 (2756.06 to 4374.3)	638473.77 (504512.14 to 797780)	3353.51 (2648.68 to 4184.51)	-0.14 (-0.16 to -0.12)
High-income Asia Pacifc	1242853.95 (981812.57 to 1553691.81)	3760.2 (2966.05 to 4704.69)	4047652.71 (3183446.73 to 5065429.51)	3920.66 (3086.65 to 4900.63)	0.32 (0.25 to 0.39)
High-income North America	3085793.04 (2446060.66 to 3846800.85)	4651.59 (3685.04 to 5800.62)	5367579.1 (4228873.02 to 6684130.09)	4400 (3468.05 to 5477.08)	-0.19 (-0.22 to -0.15)
North Africa and Middle East	980223.64 (772491.5 to 1226896.64)	4648.17 (3664.36 to 5804.84)	2545939.75 (1996439.62 to 3183096.6)	4438.63 (3481.46 to 5540.43)	-0.16 (-0.17 to -0.15)
Oceania	11556.14 (9002.22 to 14557.53)	3875.69 (3023.19 to 4856.68)	28331.35 (22117.87 to 35743.7)	3691.19 (2882.65 to 4643.46)	-0.22 (-0.32 to -0.12)
South Asia	1739240.63 (1357317.41 to 2199437.73)	2535.04 (1980.82 to 3202.11)	4859475.8 (3782922.68 to 6167358.97)	2488.75 (1937.51 to 3158.96)	-0.18 (-0.19 to -0.16)
Southeast Asia	1266218.7 (992757.63 to 1588492.28)	3837.45 (3008.57 to 4805.06)	3192320.86 (2490927.69 to 4017804.84)	3670.31 (2863.82 to 4616.83)	-0.15 (-0.16 to -0.14)
Southern Latin America	265078.06 (207716.95 to 331062.64)	3550.75 (2780.92 to 4436.37)	530317.74 (414673.26 to 666221.7)	3400.01 (2658.3 to 4272.46)	-0.15 (-0.16 to -0.13)

Southern Sub-Saharan Africa	137542.86 (107674.01 to 173397.37)	3617.19 (2828.88 to 4557.85)	252537.7 (197318.33 to 318856.94)	3452.75 (2692.7 to 4359.42)	-0.13 (-0.16 to -0.11)
Tropical Latin America	555960.2 (438915.44 to 693404.11)	4323.23 (3409.73 to 5396.41)	1792058.58 (1407735.29 to 2247161.66)	4340.51 (3412.19 to 5440.52)	-0.08 (-0.14 to -0.03)
Western Europe	4264526.45 (3475441.17 to 5147140.53)	3934.22 (3202.53 to 4753.92)	7635096.82 (6071234.35 to 9411334.36)	3856.27 (3056.38 to 4766.26)	-0.11 (-0.15 to -0.07)
Western Sub-Saharan Africa	273581.72 (213715.7 to 344130.1)	2470.11 (1928.5 to 3110.23)	524731.29 (408447.09 to 660494)	2301.74 (1791.73 to 2897.72)	-0.22 (-0.24 to -0.21)

SDI, socio-demographic index; ASPR, age-standardized prevalence rate; 95% UI, 95% Uncertainty Interval; 95% CI, 95% Confidence Interval.

S4 Table Trends in prevalence cases and ASPR of Alzheimer's disease and other dementias from 1992 to 2021 across countries and territories.

Location	Prevalence cases 1992, (95%UI)	ASPR 1992, (95%UI)	Prevalence cases 2021, (95%UI)	ASPR 2021, (95%UI)	Net Drift (%/year)
Afghanistan	37562.86 (29401.14 to 47040.08)	4473.53 (3498.95 to 5589.16)	44239.42 (34658.41 to 55598.33)	4379.2 (3427.55 to 5495.99)	-0.08 (-0.14 to -0.02)
Albania	10511.1 (8192.72 to 13223.9)	3688.26 (2873.45 to 4635.99)	27086.4 (21222.35 to 33918.67)	3708.06 (2904.6 to 4643.82)	0.05 (-0.03 to 0.12)
Algeria	71868.43 (56396.29 to 89937.96)	4507.29 (3532.7 to 5636.36)	203737.43 (159677.99 to 256049.38)	4319.51 (3380.53 to 5422.88)	-0.16 (-0.22 to -0.09)
American Samoa	93.94 (72.28 to 119.22)	3675.3 (2837.65 to 4639.6)	219.56 (168.34 to 277.82)	3584.07 (2750.92 to 4530.74)	-0.13 (-1.1 to 0.85)
Andorra	381.8 (296.96 to 480.81)	3943.43 (3067.33 to 4963.64)	1058.98 (820.79 to 1332.17)	3713.15 (2880.49 to 4671.7)	-0.19 (-0.68 to 0.31)
Angola	16280.96 (12757.74 to 20433.46)	4347.37 (3409.06 to 5429.89)	48127.56 (37844.42 to 60348.7)	4229.96 (3319.92 to 5288.79)	-0.08 (-0.17 to 0)
Antigua and Barbuda	323.07 (251.98 to 405.12)	3192.63 (2488.24 to 4007.63)	481.91 (374.81 to 609.66)	3120.2 (2425.67 to 3941.52)	-0.09 (-0.62 to 0.44)
Argentina	185650.7 (144609.99 to 232786.8)	3562.3 (2773.96 to 4468.23)	337722.08 (262787.74 to 425277.93)	3384.63 (2633.26 to 4263.27)	-0.19 (-0.21 to -0.17)
Armenia	14271.82 (11145.44 to 17841.95)	3751.24 (2930.54 to 4683.49)	27712.96 (21687.34 to 34853.15)	3727.12 (2916.14 to 4685.53)	-0.01 (-0.09 to 0.07)

Australia	135484.12 (108396.87 to 165932.93)	3938.98 (3151.33 to 4823.01)	293946.33 (238234.29 to 358250.38)	3366.94 (2720.89 to 4109.28)	-0.53 (-0.57 to -0.48)
Austria	89896.34 (70358.76 to 113104.7)	4030.21 (3150.2 to 5073.78)	141580.94 (109393.03 to 178565.56)	3766.1 (2907.37 to 4756.26)	-0.27 (-0.3 to -0.23)
Azerbaijan	26566.23 (20841.56 to 33306.8)	3721.37 (2918.93 to 4664.3)	45063.72 (35191.5 to 56982.77)	3597.72 (2810.46 to 4536.74)	-0.13 (-0.19 to -0.08)
Bahamas	738.58 (577.89 to 929.9)	3185.39 (2492.12 to 4010.41)	1769.95 (1374.95 to 2235.98)	3122.11 (2427.37 to 3939.34)	-0.07 (-0.37 to 0.24)
Bahrain	727.47 (569.08 to 910.77)	4510.94 (3524.77 to 5631.04)	3397.29 (2644.29 to 4304.88)	4399.05 (3438.5 to 5525.82)	0.03 (-0.6 to 0.68)
Bangladesh	163561.07 (127940.76 to 204898.61)	2631.61 (2056.58 to 3296.16)	493238.57 (384495.41 to 620958.53)	2538.24 (1976.69 to 3195.55)	-0.12 (-0.14 to -0.1)
Barbados	1863.77 (1458.37 to 2338.66)	3229.82 (2522.33 to 4059.71)	2782.38 (2168.97 to 3507.01)	3100.15 (2416.29 to 3907.56)	-0.15 (-0.38 to 0.08)
Belarus	81082.9 (63400.36 to 101889.71)	3817.3 (2983.53 to 4797.37)	108054.42 (84309.28 to 136110.72)	3832.77 (2990.98 to 4820.23)	0.03 (-0.01 to 0.06)
Belgium	129353.16 (103044.12 to 160092.36)	4517.99 (3594.93 to 5597.82)	195870.5 (151645.44 to 246210.3)	3990.59 (3087.11 to 5018.01)	-0.44 (-0.47 to -0.41)
Belize	527.35 (413.74 to 660.48)	3299.75 (2590.53 to 4130.76)	1318.64 (1034.2 to 1656.32)	3190.8 (2506.53 to 4002.01)	-0.1 (-0.46 to 0.26)

Benin	7415.49<br>(5858.39 to 9272.43)	2631.98<br>(2076.42 to 3289.62)	15276.76<br>(12090.48 to 19107.57)	2432.05<br>(1922.64 to 3043.93)	-0.21<br>(-0.32 to -0.11)
Bermuda	325.28<br>(254.04 to 410.02)	3295.83<br>(2571.83 to 4154.46)	852.07<br>(665.3 to 1075.25)	3244.42<br>(2533.4 to 4094.97)	-0.04<br>(-0.52 to 0.45)
Bhutan	725.7<br>(565.57 to 919.55)	2673.83<br>(2083.75 to 3374.67)	2221<br>(1727.65 to 2809.03)	2477.06<br>(1925.77 to 3131.79)	-0.24<br>(-0.57 to 0.09)
Bolivia (Plurinational State of)	11111.56<br>(8621.49 to 13972.51)	2618.08<br>(2029.9 to 3290.08)	31776.87<br>(24713.03 to 40108.64)	2595.53<br>(2019.9 to 3272.92)	0<br>(-0.08 to 0.09)
Bosnia and Herzegovina	20447.41<br>(15908.11 to 25776.56)	3664.32<br>(2854.84 to 4611.87)	40153.79<br>(31264.81 to 50348.98)	3667.4<br>(2854.05 to 4596.84)	0.02<br>(-0.04 to 0.08)
Botswana	2263.96<br>(1776.83 to 2840.17)	3541.67<br>(2775.51 to 4439.67)	5627.05<br>(4384 to 7064.84)	3413.56<br>(2658.83 to 4281.76)	-0.09<br>(-0.32 to 0.13)
Brazil	541118.46<br>(427102.36 to 675002)	4328.85<br>(3413.04 to 5404.13)	1757235.62<br>(1379987.83 to 2203367.24)	4349.63<br>(3418.34 to 5451.67)	-0.08<br>(-0.14 to -0.03)
Brunei Darussalam	420.82<br>(324.82 to 531.5)	3318.75<br>(2559.73 to 4189.52)	1231.46<br>(954.54 to 1551.64)	3313.79<br>(2568.94 to 4172.35)	0.03<br>(-0.53 to 0.59)
Bulgaria	68023.39<br>(52895.93 to 85674.13)	3772.6<br>(2927.65 to 4745.01)	96808.54<br>(75410.88 to 121553.6)	3684.75<br>(2868.45 to 4631.65)	-0.09<br>(-0.17 to -0.02)
Burkina Faso	14520.29<br>(11375.29 to 18166.57)	2722.95<br>(2127.94 to 3406.68)	28438.18<br>(22083.14 to 35968.93)	2549.65<br>(1975.16 to 3220.56)	-0.25<br>(-0.33 to -0.16)

Burundi	10119<br>(7937.36 to 12697.33)	3586.11<br>(2809.46 to 4494.69)	17152.83<br>(13399.63 to 21589.99)	3315.83<br>(2586.33 to 4166.32)	-0.23<br>(-0.34 to -0.12)
Cabo Verde	1154.22<br>(904.83 to 1449.02)	2636.09<br>(2065.78 to 3310.29)	1713.92<br>(1332.87 to 2164.46)	2510.73<br>(1954.67 to 3166.18)	-0.18<br>(-0.45 to 0.09)
Cambodia	20673.34<br>(16155.11 to 25966.56)	3917.82<br>(3054.82 to 4912.86)	56031.92<br>(43590.4 to 70654.52)	3762.02<br>(2921.36 to 4734.94)	-0.19<br>(-0.25 to -0.12)
Cameroon	13493.89<br>(10551.99 to 16990.67)	2525.26<br>(1973.3 to 3178.29)	33308.59<br>(25861.68 to 42119.81)	2399.08<br>(1859.56 to 3031.71)	-0.18<br>(-0.26 to -0.1)
Canada	310760.08<br>(256084.82 to 368104.9)	5325.19<br>(4389.5 to 6309.61)	614963.95<br>(500541.77 to 737248.94)	4478.65<br>(3643.58 to 5369.27)	-0.64<br>(-0.68 to -0.6)
Central African Republic	4900.33<br>(3844.74 to 6112.11)	4676.79<br>(3685.28 to 5782.59)	8403.94<br>(6595.91 to 10540.59)	4567.16<br>(3610.88 to 5674.98)	-0.15<br>(-0.33 to 0.02)
Chad	10366.41<br>(8098.43 to 13003.69)	2674.76<br>(2086.27 to 3355.69)	15848.32<br>(12341.78 to 19978.26)	2434.9<br>(1892.86 to 3065.35)	-0.34<br>(-0.44 to -0.25)
Chile	54350.07<br>(42943.77 to 67262.23)	3480.71<br>(2746.75 to 4310.11)	154238.82<br>(120486.65 to 193599.71)	3432.65<br>(2681.16 to 4309.7)	0.01<br>(-0.03 to 0.05)
China	4311149.61<br>(3358407.99 to 5439974.58)	4247.3<br>(3313.58 to 5353.14)	16417647.94<br>(12751702.83 to 20668160.17)	5180.9<br>(4033.4 to 6513.45)	0.38<br>(0.34 to 0.41)
Colombia	94603.14<br>(74219.48 to 118296.05)	3694.74<br>(2898.53 to 4617.5)	351755.34<br>(274897.25 to 443002.09)	3658.34<br>(2863.45 to 4603.13)	-0.04<br>(-0.06 to -0.01)

Comoros	792.16 (621.48 to 993.44)	3473.35 (2718.97 to 4348.66)	2049.87 (1606.99 to 2562.62)	3322.52 (2599.58 to 4150.54)	-0.12 (-0.48 to 0.24)
Congo	4894.52 (3825.46 to 6132.46)	4218.46 (3296.98 to 5271.97)	10749.91 (8943.29 to 12813.86)	4038.55 (3375.66 to 4788.97)	-0.12 (-0.34 to 0.1)
Cook Islands	60.99 (46.93 to 76.9)	3693.12 (2845.49 to 4650.32)	156.8 (121.04 to 197.99)	3611.64 (2786.56 to 4561.47)	-0.2 (-1.33 to 0.95)
Costa Rica	10764.69 (8417.9 to 13485.57)	3688.14 (2883.53 to 4619.91)	33986.74 (26456.06 to 42869.18)	3612.94 (2815.9 to 4553.58)	-0.07 (-0.15 to 0)
Coted'Ivoire	10507.51 (8203.81 to 13283.54)	2541.5 (1979.78 to 3208.88)	28686.62 (22244.93 to 36289.61)	2441.55 (1890.81 to 3086.31)	-0.14 (-0.23 to -0.05)
Croatia	34286.48 (26717.7 to 43159.01)	3816.77 (2976.87 to 4799.07)	64002.59 (49626.9 to 80761.03)	3703.23 (2869.89 to 4677.33)	-0.06 (-0.12 to -0.01)
Cuba	53371.08 (42577.04 to 65950.21)	3019.87 (2404.43 to 3736)	107870.41 (84749.9 to 134213.98)	3009.35 (2364.93 to 3743.45)	-0.23 (-0.27 to -0.19)
Cyprus	4590.8 (3546.71 to 5798.33)	3952.68 (3061.02 to 4978.53)	13386.72 (10379.36 to 16862.99)	3806.87 (2948.52 to 4801.97)	-0.1 (-0.39 to 0.19)
Czechia	84625.81 (65910.86 to 106647.74)	3697.54 (2877.22 to 4656.41)	147496.82 (114950.26 to 185482.88)	3651.05 (2844.38 to 4594.1)	-0.06 (-0.09 to -0.03)
Democratic People's Republic of Korea	69301.28 (53537.96 to 87358.67)	3631.58 (2810.08 to 4564.24)	169741.86 (131995 to 214024.56)	3554.54 (2759.31 to 4479.77)	-0.11 (-0.15 to -0.07)

Democratic Republic of the Congo	70120.84<br>(54918.88 to 87793.26)	4289.49<br>(3366.86 to 5352.23)	158820.04<br>(124922.84 to 198844.49)	4331.11<br>(3411.87 to 5406.11)	0.01<br>(-0.03 to 0.05)
Denmark	52517.92<br>(41885.92 to 64792.26)	3346.32<br>(2662.29 to 4138.21)	63232.47<br>(49592.88 to 78871.22)	2712.14<br>(2123.22 to 3387.58)	-0.78<br>(-0.85 to -0.71)
Djibouti	481.59<br>(378.04 to 603.55)	3622.11<br>(2841.82 to 4528.78)	2067.1<br>(1621.34 to 2607.8)	3443.69<br>(2696.46 to 4334.91)	-0.1<br>(-0.55 to 0.34)
Dominica	327.13<br>(256.68 to 411.33)	3251.91<br>(2547.1 to 4092.96)	406.95<br>(316.57 to 512.18)	3160.05<br>(2457.61 to 3975.24)	-0.09<br>(-0.65 to 0.47)
Dominican Republic	18317.32<br>(14508.93 to 22751.17)	3278.76<br>(2592.56 to 4073.72)	52487.46<br>(41244.69 to 65305.01)	3282.86<br>(2580.24 to 4083.35)	-0.15<br>(-0.21 to -0.08)
Ecuador	21274.9<br>(16638.05 to 26716.6)	2631.57<br>(2056.86 to 3303.59)	67118.68<br>(52065.38 to 84698.82)	2587.7<br>(2004.81 to 3266.2)	-0.02<br>(-0.08 to 0.04)
Egypt	121768.24<br>(98083.8 to 149292.76)	4259.33<br>(3427.72 to 5214.62)	265068.38<br>(209078.41 to 330413.93)	4179.96<br>(3287.6 to 5197.44)	-0.05<br>(-0.08 to -0.02)
El Salvador	17986.18<br>(14078.33 to 22540.28)	3603.39<br>(2823.6 to 4513.76)	42110.67<br>(32850.64 to 52962.04)	3654.1<br>(2854.08 to 4596.48)	0.08<br>(0.01 to 0.14)
Equatorial Guinea	898.02<br>(705.36 to 1123.97)	4408.84<br>(3465.83 to 5497.91)	2277.7<br>(1785.3 to 2852.56)	4218.45<br>(3307.29 to 5273.49)	-0.17<br>(-0.52 to 0.19)
Eritrea	3091.18<br>(2421.56 to 3884.79)	3654.85<br>(2861.78 to 4565.23)	8930.26<br>(6962.79 to 11224.49)	3443.75<br>(2682.58 to 4319.22)	-0.17<br>(-0.4 to 0.06)

Estonia	12673.67<br>(9904.44 to 15903.66)	3758.11<br>(2932.23 to 4718.17)	20848.9<br>(16234.33 to 26117.01)	3716.28<br>(2891.1 to 4661.3)	0<br>(-0.09 to 0.09)
Eswatini	1114.74<br>(870.89 to 1401.05)	3428.25<br>(2670.73 to 4301.89)	1883.55<br>(1474.51 to 2370.81)	3309.51<br>(2584.53 to 4168.28)	-0.14<br>(-0.46 to 0.18)
Ethiopia	72023.97<br>(56298.57 to 90812.25)	3579.55<br>(2794.3 to 4510.8)	179526.66<br>(140266.59 to 227375.34)	3324.81<br>(2593.13 to 4210.23)	-0.21<br>(-0.26 to -0.16)
Fiji	1376.63<br>(1062.35 to 1749.64)	3730.24<br>(2881.74 to 4717.82)	3115.48<br>(2406.15 to 3943.01)	3652.21<br>(2828.58 to 4611.51)	-0.11<br>(-0.39 to 0.17)
Finland	51637.43<br>(40526.77 to 64063.93)	3958.5<br>(3103.79 to 4918.62)	94301.31<br>(73001.57 to 119406.8)	3546.68<br>(2740.95 to 4495.06)	-0.35<br>(-0.4 to -0.31)
France	515132.75<br>(432378.5 to 605072.98)	3160.46<br>(2643.96 to 3727.02)	911446.25<br>(739609.75 to 1105586.18)	3015.61<br>(2434.79 to 3669.2)	-0.17<br>(-0.2 to -0.15)
Gabon	3282.3<br>(2567.95 to 4106.52)	4299.74<br>(3362.66 to 5366.35)	4959.45<br>(3882.43 to 6226.72)	4204.57<br>(3289.36 to 5264.57)	-0.08<br>(-0.26 to 0.09)
Gambia	1140.48<br>(892.99 to 1430.04)	2682.72<br>(2095.7 to 3362.99)	3007.08<br>(2335.52 to 3792.06)	2487.91<br>(1929.37 to 3138.07)	-0.26<br>(-0.54 to 0.02)
Georgia	36581.75<br>(28599.71 to 45698.73)	3727.59<br>(2913.14 to 4655.96)	42691.21<br>(33362.51 to 53488.76)	3728.95<br>(2917.47 to 4670.6)	0.02<br>(-0.03 to 0.08)
Germany	1095920.96<br>(906599.25 to 1311020.89)	4658.5<br>(3853.38 to 5578.83)	1971203.22<br>(1581888.67 to 2396957.89)	4730.91<br>(3781.7 to 5782.38)	0.14<br>(0.01 to 0.28)

Ghana	17814.22<br>(13901.85 to 22495.58)	2440.29<br>(1899.21 to 3077.24)	44875.53<br>(34879.69 to 56561.01)	2390.77<br>(1854.91 to 3010.65)	-0.05<br>(-0.12 to 0.02)
Greece	107772.14<br>(83819.21 to 135944.24)	4042.45<br>(3141.75 to 5099.7)	209689.24<br>(163247.41 to 262545)	3866.11<br>(3004.17 to 4853.19)	-0.14<br>(-0.18 to -0.11)
Greenland	162.08<br>(125.61 to 204.16)	4645.18<br>(3597.92 to 5831.75)	379.02<br>(294.1 to 478)	4487.83<br>(3482.01 to 5643.21)	-0.09<br>(-0.91 to 0.73)
Grenada	466.74<br>(365.36 to 584.02)	3265.84<br>(2554.91 to 4089.32)	495.57<br>(386.32 to 624.5)	3221.05<br>(2509.21 to 4055.68)	-0.04<br>(-0.51 to 0.42)
Guam	318.85<br>(245.17 to 404.9)	3614.96<br>(2783.66 to 4580.8)	1370.18<br>(1059.09 to 1732.18)	3602.9<br>(2791.31 to 4549.34)	-0.08<br>(-0.61 to 0.45)
Guatemala	15445.73<br>(12061.81 to 19434.54)	3694.3<br>(2886.27 to 4648.35)	59407.47<br>(46569.23 to 74188.5)	3664.63<br>(2869.9 to 4579.1)	-0.01<br>(-0.1 to 0.07)
Guinea	12456.49<br>(9757.05 to 15595.72)	2651.97<br>(2074.54 to 3322.38)	17789.94<br>(13850.71 to 22379.08)	2496.69<br>(1943.39 to 3141.38)	-0.23<br>(-0.31 to -0.14)
Guinea-Bissau	1067.69<br>(830.22 to 1346.18)	2516.36<br>(1952.46 to 3169.81)	1690.72<br>(1314.38 to 2145.28)	2456.98<br>(1908.08 to 3116.36)	-0.09<br>(-0.44 to 0.26)
Guyana	1566.07<br>(1222.2 to 1966.83)	3179.76<br>(2483.55 to 3986.29)	2589.69<br>(2013.79 to 3259.55)	3141.69<br>(2448.13 to 3943.25)	-0.04<br>(-0.28 to 0.19)
Haiti	12291.77<br>(9619.04 to 15398.34)	3334.73<br>(2610.21 to 4166.5)	24481.3<br>(19191.52 to 30855.46)	3099.96<br>(2434.31 to 3896.95)	-0.28<br>(-0.37 to -0.18)

Honduras	10867.12 (8494.1 to 13551.33)	3734.99 (2915.57 to 4655.54)	30259.63 (23679.81 to 37872.29)	3675.08 (2872.59 to 4602.23)	-0.07 (-0.15 to 0.02)
Hungary	87923.33 (68469.23 to 110277.78)	3686.71 (2867.54 to 4625.18)	136488.39 (106326 to 171889.5)	3652.3 (2844.61 to 4600.79)	-0.02 (-0.06 to 0.01)
Iceland	2378.76 (1886.16 to 2903.47)	4361.87 (3456.49 to 5328.9)	4455.07 (3533.25 to 5453.59)	3937.75 (3121.43 to 4819.97)	-0.37 (-0.57 to -0.18)
India	1339842.43 (1044967.69 to 1696964.04)	2497.84 (1950.9 to 3160.15)	3938968.82 (3061050.01 to 5001190.01)	2482.36 (1928.3 to 3151.81)	-0.15 (-0.17 to -0.14)
Indonesia	455850.34 (354364.13 to 576915.17)	3863.27 (3004.27 to 4884.08)	1023891.13 (790476.33 to 1301932.01)	3766.7 (2910.54 to 4786.14)	-0.09 (-0.1 to -0.08)
Iran (Islamic Republic of)	135222.14 (105494.94 to 170912.73)	4591.21 (3592.86 to 5772.9)	481638.52 (375492.73 to 606974.17)	4439.23 (3462.57 to 5591.15)	-0.09 (-0.11 to -0.06)
Iraq	54009.28 (42217.08 to 67621.92)	4579.4 (3581.77 to 5729.2)	118334.69 (92238.9 to 148513.74)	4366.34 (3404.51 to 5464.77)	-0.2 (-0.23 to -0.16)
Ireland	28216.29 (21921.85 to 35427.95)	3978.23 (3087.11 to 4996.12)	52773.35 (40762.95 to 66340.31)	3625.84 (2798.33 to 4560.66)	-0.32 (-0.38 to -0.26)
Israel	33523.06 (26094.2 to 42229.26)	3877.2 (3013.23 to 4880.22)	85252.5 (66077.1 to 107713.76)	3662.91 (2837.97 to 4627.26)	-0.21 (-0.26 to -0.16)
Italy	648542.85 (508483.92 to 803191.06)	3942.19 (3086.99 to 4892.56)	1407328.98 (1093344.44 to 1778889.31)	4361.16 (3381.11 to 5519.76)	0.04 (-0.16 to 0.24)

Jamaica	12370.99 (9692.8 to 15498.23)	3670.09 (2874.36 to 4600.93)	19701.47 (15317.27 to 24728.6)	3487.45 (2714.17 to 4371.33)	-0.21 (-0.29 to -0.13)
Japan	1077507.37 (844875.9 to 1355880.61)	3706.61 (2903.08 to 4667.07)	3326449.52 (2602773.86 to 4194371.13)	3857.34 (3022.35 to 4861.2)	0.34 (0.26 to 0.41)
Jordan	6913.74 (5397.2 to 8701.35)	4460.43 (3482.74 to 5596.37)	38367.12 (30089.18 to 48038.8)	4519.31 (3546.72 to 5656.87)	0.09 (0 to 0.19)
Kazakhstan	65116.56 (50911.3 to 81474.48)	3718.97 (2905.22 to 4653.4)	83639.7 (65282.21 to 105043.15)	3627.59 (2826.86 to 4543.54)	-0.07 (-0.11 to -0.03)
Kenya	37377.11 (29189.41 to 47137.45)	3490.54 (2723.75 to 4402.52)	86918.17 (67991.24 to 109701.45)	3440.76 (2688.41 to 4339.96)	-0.07 (-0.11 to -0.02)
Kiribati	154.18 (119.88 to 194.38)	4025.97 (3137.53 to 5056.45)	285.52 (222.15 to 360.31)	4029.23 (3139.71 to 5054.05)	0.14 (-0.77 to 1.07)
Kuwait	3211.82 (2529.54 to 4000.67)	4663.31 (3677.58 to 5797.3)	14670.71 (11484.6 to 18457.83)	4430.63 (3474.03 to 5560.7)	-0.17 (-0.3 to -0.04)
Kyrgyzstan	16337.2 (12756.45 to 20404.69)	3686.38 (2876.18 to 4602.82)	22312.73 (17466.66 to 27808.57)	3718.98 (2909.12 to 4624)	0.07 (-0.01 to 0.15)
Lao People's Democratic Republic	9107.74 (7107.4 to 11456.9)	3832.45 (2987.2 to 4801.42)	20453.11 (15947.64 to 25662.66)	3746.59 (2919.91 to 4694.38)	-0.06 (-0.18 to 0.06)
Latvia	22714.53 (17652.64 to 28528.56)	3761.89 (2922.35 to 4726.35)	31055.81 (24339.09 to 38984.72)	3786.02 (2963.65 to 4759.02)	0.06 (-0.01 to 0.13)

Lebanon	14331.88 (11229.06 to 17949.06)	4781.22 (3747.87 to 5973.55)	53015.58 (41688.23 to 66099.04)	4758.96 (3741.55 to 5939.08)	-0.01 (-0.08 to 0.05)
Lesotho	4175.17 (3271.21 to 5216.17)	3618.75 (2827.99 to 4522.56)	4254.42 (3333.52 to 5349.24)	3580.07 (2800.73 to 4501.94)	-0.04 (-0.2 to 0.13)
Liberia	3744.26 (2926.74 to 4687.03)	2471.97 (1926.73 to 3099.5)	5576.48 (4358.6 to 7019.33)	2388.6 (1868.47 to 3001.08)	-0.14 (-0.3 to 0.03)
Libya	12624.01 (9905.03 to 15753.67)	4598.33 (3610.88 to 5730.87)	28376.13 (22149.56 to 35495.51)	4382.37 (3423.84 to 5475.31)	-0.16 (-0.23 to -0.09)
Lithuania	28480.3 (22270.38 to 35757.58)	3677.32 (2874.49 to 4619.4)	44226.83 (34531.55 to 55399.65)	3715.42 (2899.04 to 4658.9)	0.05 (-0.01 to 0.11)
Luxembourg	3080.64 (2388.45 to 3880.8)	3175.48 (2456.89 to 4005.23)	5477.07 (4270.47 to 6968.31)	2719.08 (2118.8 to 3461.5)	-0.48 (-0.66 to -0.31)
Madagascar	20596.03 (16206.04 to 25827.74)	3444.82 (2706.9 to 4311.68)	34817.35 (27173.19 to 44003.27)	3283.6 (2561.71 to 4133.69)	-0.15 (-0.22 to -0.08)
Malawi	15182.68 (11880.39 to 19119.1)	3473.73 (2709.64 to 4371.51)	28038.09 (21987.16 to 35168.54)	3459.13 (2706.22 to 4334.58)	0 (-0.09 to 0.08)
Malaysia	53298.61 (41659.54 to 66921.9)	3974.26 (3107.56 to 4983.37)	146354.75 (113353.88 to 184444.96)	3750.85 (2903.95 to 4725.73)	-0.14 (-0.18 to -0.11)
Maldives	368.28 (287.34 to 464.56)	3687.17 (2873.02 to 4638.21)	1669.75 (1304.22 to 2096.83)	3808.24 (2973.56 to 4778.21)	0.06 (-0.47 to 0.59)

Mali	11512.71<br>(8979.84 to 14443.49)	2610.12<br>(2031.44 to 3266.8)	24687.65<br>(19269.74 to 30965.01)	2501.05<br>(1949.92 to 3133.61)	-0.15<br>(-0.24 to -0.05)
Malta	2797.97<br>(2174.86 to 3527.99)	3979.4<br>(3094.45 to 5014.19)	7214.19<br>(5597.03 to 9094.02)	3700.16<br>(2866.94 to 4668.64)	-0.23<br>(-0.39 to -0.06)
Marshall Islands	62.54<br>(47.91 to 79.16)	3528.87<br>(2711.49 to 4458.33)	105.3<br>(80.59 to 134.66)	3398.14<br>(2618.87 to 4312.09)	0.22<br>(-1.75 to 2.23)
Mauritania	3503.16<br>(2736.14 to 4409.77)	2650.4<br>(2064.48 to 3334.49)	6971.95<br>(5436.41 to 8778.8)	2464.71<br>(1920.36 to 3106.29)	-0.24<br>(-0.4 to -0.08)
Mauritius	3681.62<br>(2864.52 to 4645.65)	3778.54<br>(2936.71 to 4754.18)	10750.64<br>(8364.47 to 13563.51)	3753.41<br>(2922.75 to 4728.28)	0.03<br>(-0.1 to 0.17)
Mexico	198296.86<br>(154499.86 to 251109.84)	3242.87<br>(2525.21 to 4109.73)	579284.4<br>(449874.19 to 734079.68)	3042.79<br>(2362.79 to 3856.57)	-0.15<br>(-0.16 to -0.13)
Micronesia (Federated States of)	235.32<br>(182.32 to 296.19)	3958.09<br>(3070.01 to 4964.39)	300.07<br>(231.87 to 379.9)	3988.39<br>(3092.34 to 5021.25)	0.02<br>(-0.7 to 0.74)
Monaco	606.42<br>(471.45 to 764.88)	3986.68<br>(3092.63 to 5034.12)	758.9<br>(583.92 to 958.12)	3677.83<br>(2825.22 to 4650.33)	-0.29<br>(-0.73 to 0.16)
Mongolia	5105.4<br>(4005.77 to 6392.85)	3741.68<br>(2936.43 to 4681.4)	9541.22<br>(7463.09 to 11941.6)	3780.45<br>(2955.66 to 4731.43)	0.08<br>(-0.06 to 0.22)
Montenegro	3772.47<br>(2944.92 to 4730.18)	3773.78<br>(2944.48 to 4730.02)	5588.22<br>(4349.69 to 7013.77)	3658.37<br>(2846.5 to 4587.82)	-0.15<br>(-0.3 to -0.01)

Morocco	94732.4<br>(74368.92 to 118826.49)	4594.23<br>(3605.74 to 5756.47)	201920.35<br>(157378.89 to 252906.42)	4298.91<br>(3347.71 to 5378.61)	-0.25<br>(-0.28 to -0.22)
Mozambique	24129.63<br>(18888.73 to 30299.88)	3556.95<br>(2775.65 to 4456.08)	39682.4<br>(31027.67 to 49826.11)	3459.53<br>(2702.51 to 4332.62)	-0.09<br>(-0.16 to -0.03)
Myanmar	114747.36<br>(90148.7 to 144012.84)	4088.19<br>(3210.06 to 5119.4)	243890.28<br>(190522.54 to 307251.28)	3790.01<br>(2959.6 to 4768.43)	-0.27<br>(-0.3 to -0.24)
Namibia	2623.03<br>(2050.65 to 3292.63)	3509.27<br>(2736.85 to 4391.89)	5378.66<br>(4204.23 to 6754.45)	3391.52<br>(2644.24 to 4259.19)	-0.11<br>(-0.32 to 0.09)
Nauru	15.97<br>(12.31 to 20.21)	3660.55<br>(2832.52 to 4617.51)	21.57<br>(16.63 to 27.35)	3739.5<br>(2892.03 to 4736.74)	NA
Nepal	31602.48<br>(24745.69 to 39603.26)	2915.36<br>(2281.23 to 3646.31)	79222.61<br>(61688.87 to 99697.48)	2595.97<br>(2017.35 to 3264.66)	-0.48<br>(-0.53 to -0.43)
Netherlands	150466.09<br>(122863.56 to 179443.18)	4075.43<br>(3327.73 to 4864.39)	274552.43<br>(218080.68 to 337001.82)	4036.81<br>(3202.49 to 4960.56)	0.13<br>(0.01 to 0.24)
New Zealand	28758.69<br>(22438.55 to 36145.94)	4143.93<br>(3230.03 to 5213.06)	61334.79<br>(47393.48 to 77567.16)	3903.28<br>(3015.61 to 4937.14)	-0.26<br>(-0.32 to -0.21)
Nicaragua	8661.83<br>(6799.56 to 10827.68)	3758.09<br>(2949.07 to 4697.99)	26586.61<br>(20815.35 to 33270.38)	3752.11<br>(2936.04 to 4695.08)	0.01<br>(-0.07 to 0.1)
Niger	7950.27<br>(6223.48 to 9992.58)	2662.33<br>(2083.02 to 3347.35)	22647.2<br>(17648.06 to 28530.76)	2499.66<br>(1946.19 to 3146.67)	-0.22<br>(-0.33 to -0.11)

Nigeria	134604.44<br>(104695.69 to 170814.73)	2355.15<br>(1828.27 to 2993.08)	228682.97<br>(176876.84 to 290873.86)	2129.93<br>(1646.74 to 2710.56)	-0.31<br>(-0.33 to -0.28)
Niue	14.66<br>(11.32 to 18.55)	3637.6<br>(2805.84 to 4604.78)	12.05<br>(9.23 to 15.31)	3526.68<br>(2701.93 to 4477.31)	-0.08<br>(-3.18 to 3.13)
North Macedonia	9568.87<br>(7427.26 to 12087.67)	3664.97<br>(2846.95 to 4624.54)	16575.91<br>(12829.91 to 20987.19)	3640.91<br>(2820.77 to 4601.22)	-0.01<br>(-0.12 to 0.1)
Northern Mariana Islands	52.49<br>(40.17 to 66.68)	3608.8<br>(2771.22 to 4565.83)	202.04<br>(154.2 to 257.7)	3552.55<br>(2719.31 to 4509.57)	0.04<br>(-1.83 to 1.94)
Norway	59468.58<br>(46736.89 to 74478.08)	4361.51<br>(3422.44 to 5469.2)	72425.78<br>(55884.93 to 91834.27)	3665.48<br>(2826.27 to 4648.52)	-0.66<br>(-0.7 to -0.62)
Oman	3531.97<br>(2758.84 to 4452.37)	4482.19<br>(3499.62 to 5635.35)	8139.23<br>(6281.16 to 10276.01)	4182.42<br>(3226.83 to 5270.11)	-0.19<br>(-0.34 to -0.04)
Pakistan	203508.96<br>(158689.96 to 257598.47)	2641.06<br>(2056.39 to 3345.45)	345824.8<br>(268303.59 to 439686.6)	2466.19<br>(1914.52 to 3133.41)	-0.27<br>(-0.29 to -0.24)
Palau	43.81<br>(33.48 to 55.58)	3511.68<br>(2690.77 to 4451.46)	84.43<br>(64.17 to 107.44)	3396.04<br>(2589.8 to 4303.53)	0.04<br>(-2.19 to 2.31)
Palestine	5436.4<br>(4251.77 to 6787.53)	4631.35<br>(3622.88 to 5772.75)	12889.43<br>(10104.38 to 16178.85)	4445.08<br>(3486.46 to 5564.56)	-0.17<br>(-0.29 to -0.04)
Panama	8750.75<br>(6833.98 to 10980.04)	3597.23<br>(2808.66 to 4511.77)	27270.94<br>(21271.64 to 34290.27)	3550.55<br>(2772.12 to 4461.2)	-0.05<br>(-0.14 to 0.03)

Papua New Guinea	6943.43<br>(5422.23 to 8745.05)	3970.58<br>(3101.61 to 4977.46)	18037.75<br>(14039.38 to 22720.59)	3719.13<br>(2897.72 to 4672.65)	-0.29<br>(-0.42 to -0.15)
Paraguay	14841.73<br>(11662.27 to 18608.69)	4170.11<br>(3277.22 to 5225.97)	34822.96<br>(27338.42 to 43622.45)	3929.45<br>(3087.22 to 4918.64)	-0.23<br>(-0.3 to -0.17)
Peru	47209.61<br>(36991.88 to 58963.88)	2524.18<br>(1978.24 to 3150.01)	137764.01<br>(107431.35 to 172615.38)	2477.54<br>(1934.21 to 3102.77)	-0.1<br>(-0.14 to -0.07)
Philippines	149899.65<br>(117183.18 to 188715.97)	3941.33<br>(3079.05 to 4967.37)	392596.96<br>(305766.54 to 496749.55)	3774.4<br>(2940.37 to 4769.87)	-0.2<br>(-0.24 to -0.17)
Poland	270700.08<br>(210085.47 to 341621.95)	3892.61<br>(3021.56 to 4913.62)	490468.54<br>(379554.22 to 621278.46)	3683.44<br>(2850.94 to 4664.09)	-0.18<br>(-0.2 to -0.17)
Portugal	90149.1<br>(70021.69 to 113474.64)	3869.46<br>(3002.84 to 4867.49)	200316.38<br>(155935.41 to 251758.92)	3792.84<br>(2949.63 to 4773.55)	-0.04<br>(-0.07 to 0)
Puerto Rico	21105.92<br>(16579.03 to 26414.28)	3292.47<br>(2582.29 to 4123.85)	49757.37<br>(38833.23 to 62882.16)	3223.97<br>(2519.19 to 4072.14)	-0.09<br>(-0.15 to -0.02)
Qatar	361.98<br>(282.92 to 456.44)	4331.31<br>(3392.43 to 5436.25)	2733.45<br>(2122.01 to 3452.04)	4256.33<br>(3298.42 to 5356.96)	-0.01<br>(-0.56 to 0.53)
Republic of Korea	155890<br>(124543.52 to 190290.69)	4304.84<br>(3446.79 to 5245.21)	677527.26<br>(538806.9 to 833295.36)	4242.29<br>(3373.46 to 5217.28)	0.04<br>(-0.06 to 0.13)
Republic of Moldova	21675.72<br>(16980.3 to 27211.06)	3638.61<br>(2851.56 to 4562.84)	38138.63<br>(29740.86 to 47861.61)	3668.87<br>(2861.54 to 4598.89)	0.03<br>(-0.04 to 0.1)

Romania	150169 (117339.68 to 188802.78)	3657.44 (2853.8 to 4597)	258794.74 (201431.16 to 325755.89)	3646.73 (2838.14 to 4590.05)	-0.04 (-0.07 to -0.01)
Russian Federation	1047024.56 (814675.82 to 1321260.75)	3847.88 (2993.43 to 4859.74)	1564471.03 (1221156.56 to 1974856.61)	3777.25 (2948.26 to 4765.2)	-0.1 (-0.11 to -0.08)
Rwanda	10593.12 (8303.73 to 13309.1)	3519.62 (2750.19 to 4414.3)	24658.66 (19329 to 30863.46)	3521.12 (2758.13 to 4402.65)	0.05 (-0.06 to 0.16)
Saint Kitts and Nevis	195.91 (152.35 to 247.51)	3095.69 (2399.24 to 3912.72)	261.58 (202.88 to 330.27)	3054.2 (2375.13 to 3847.98)	-0.14 (-0.91 to 0.64)
Saint Lucia	445.43 (347.61 to 559.64)	3261.75 (2541.52 to 4098.62)	1234.26 (961.02 to 1554.4)	3155.46 (2456.85 to 3973.68)	-0.16 (-0.63 to 0.31)
Saint Vincent and the Grenadines	384.28 (301.35 to 480.95)	3318.91 (2600.5 to 4157.36)	708.61 (553.08 to 889.56)	3169.31 (2471.96 to 3978.93)	-0.16 (-0.67 to 0.36)
Samoa	396.3 (306.43 to 500.85)	3690.04 (2859.56 to 4653.68)	676.37 (520.52 to 857.17)	3563.22 (2742.56 to 4511.71)	-0.16 (-0.63 to 0.32)
San Marino	286.58 (224.91 to 358.38)	4032.48 (3160.33 to 5048.24)	595.59 (460.96 to 750.62)	3585.5 (2775.03 to 4523.51)	-0.44 (-0.99 to 0.11)
Sao Tome and Principe	229.7 (179.34 to 289.24)	2506.95 (1955.22 to 3155.78)	313.39 (242.75 to 397.65)	2339.45 (1812.88 to 2967.4)	-0.25 (-0.87 to 0.38)
Saudi Arabia	30140.26 (23406.54 to 37893.6)	4296.55 (3336.24 to 5395.05)	62969.13 (48730.6 to 80078.65)	4029.86 (3125.32 to 5108.08)	-0.22 (-0.27 to -0.16)

Senegal	10949.97<br>(8539.77 to 13762.13)	2623.19<br>(2043.44 to 3299.8)	23899.62<br>(18596.68 to 30225.24)	2445.36<br>(1900.28 to 3089.57)	-0.27<br>(-0.36 to -0.18)
Serbia	52909.62<br>(40980.85 to 66842.21)	3657.19<br>(2844.92 to 4603.35)	109348.62<br>(84749.04 to 137891.21)	3652.72<br>(2829.47 to 4606.31)	0<br>(-0.04 to 0.05)
Seychelles	368.69<br>(285.77 to 465.58)	3818.17<br>(2959.69 to 4821.43)	603.95<br>(466.84 to 762.6)	3675<br>(2841.74 to 4637.8)	-0.15<br>(-0.62 to 0.31)
Sierra Leone	7456.75<br>(5860.11 to 9370.92)	2665.45<br>(2087.23 to 3355.21)	11527.11<br>(9019.72 to 14576.97)	2521.32<br>(1968.5 to 3188.92)	-0.2<br>(-0.31 to -0.09)
Singapore	9035.77<br>(7217.55 to 10989.41)	2944.57<br>(2359.87 to 3572.45)	42444.47<br>(34664.34 to 51084.56)	3066.96<br>(2507.94 to 3682.01)	0.12<br>(0.04 to 0.21)
Slovakia	35813.74<br>(27970.51 to 45020.07)	3676.73<br>(2870.77 to 4617.58)	59475.05<br>(46435.77 to 74884.04)	3633.16<br>(2836.29 to 4572.64)	-0.05<br>(-0.1 to 0)
Slovenia	15391.35<br>(11988.44 to 19387.32)	3622.11<br>(2818.71 to 4564.59)	32575.04<br>(25355.79 to 40889.79)	3635.89<br>(2830.44 to 4565.21)	0.06<br>(-0.01 to 0.13)
Solomon Islands	507.15<br>(391.77 to 641.32)	3722.27<br>(2881.97 to 4680.27)	1328.42<br>(1029.28 to 1677.96)	3737.88<br>(2896.66 to 4715.92)	0.06<br>(-0.49 to 0.62)
Somalia	7589.29<br>(5952.3 to 9525.73)	3549.67<br>(2782.63 to 4439.18)	18337.59<br>(14425.83 to 23021.81)	3528.66<br>(2768.66 to 4424.85)	-0.02<br>(-0.15 to 0.11)
South Africa	110152.02<br>(85927.59 to 139296.16)	3650.91<br>(2847.04 to 4614.1)	211927.67<br>(165139.15 to 268412.45)	3464.77<br>(2695.88 to 4386.3)	-0.15<br>(-0.18 to -0.13)

South Sudan	11099.57 (8725.87 to 13917.59)	3480.52 (2730.98 to 4368.01)	13270.07 (10356.8 to 16730.25)	3262.53 (2542.62 to 4102.61)	-0.2 (-0.3 to -0.1)
Spain	371172.69 (307832.44 to 437169.25)	3707.77 (3075.1 to 4370.87)	701126.5 (554223.98 to 870493.74)	3338.63 (2637.61 to 4149.49)	-0.29 (-0.33 to -0.26)
Sri Lanka	53642.45 (42117.18 to 67357.94)	3737.9 (2933.14 to 4688.63)	146754.38 (113841.79 to 185182.09)	3613.97 (2805.17 to 4555.35)	-0.13 (-0.17 to -0.08)
Sudan	55947.22 (43832.04 to 69820.49)	4518.44 (3541.76 to 5633.43)	99164.88 (77854.57 to 124800.44)	4204.82 (3301.28 to 5286.5)	-0.26 (-0.3 to -0.22)
Suriname	1308.71 (1023.59 to 1635.72)	3442.65 (2697.54 to 4294.45)	3184.64 (2489.61 to 4002.63)	3287.32 (2571.56 to 4130.01)	-0.19 (-0.41 to 0.03)
Sweden	129064.7 (102629.67 to 159681.31)	4207.58 (3338.58 to 5214.23)	182779.07 (141859.14 to 230033)	4006.42 (3103.57 to 5054.38)	-0.01 (-0.09 to 0.06)
Switzerland	85275.67 (66848.78 to 106499.37)	4113.57 (3220.06 to 5146.37)	139426.57 (108021.69 to 175387.24)	3682.04 (2850.47 to 4636.36)	-0.41 (-0.45 to -0.38)
Syrian Arab Republic	30901.02 (24255.12 to 38739.7)	4677.5 (3673.8 to 5848.6)	70165.33 (54836.11 to 88454.05)	4328.9 (3380.15 to 5446)	-0.28 (-0.33 to -0.22)
Taiwan (Province of China)	60492.6 (47560.4 to 75183.46)	2888.7 (2273.83 to 3579.64)	240328.37 (189504.07 to 294220.75)	3185.2 (2513.11 to 3892.56)	0.53 (0.47 to 0.6)
Tajikistan	14422.19 (11288.06 to 18086.86)	3635.4 (2845.93 to 4555.96)	21985.23 (17129.35 to 27842.42)	3409.07 (2657.96 to 4302.82)	-0.22 (-0.3 to -0.14)

Thailand	164508.26 (130728.4 to 204288.04)	3402.82 (2703.64 to 4220.01)	636452.54 (496939.5 to 799672.45)	3442.75 (2690.06 to 4323.74)	0.15 (0.12 to 0.18)
Timor-Leste	1175.09 (921.48 to 1476.47)	4025.01 (3157.87 to 5040.53)	4286.83 (3357.21 to 5381.11)	3794.97 (2967.48 to 4764.75)	-0.2 (-0.44 to 0.05)
Togo	3685.33 (2881.17 to 4636.26)	2575.41 (2010.63 to 3237.68)	9783.11 (7588.2 to 12388.43)	2497.19 (1935.19 to 3152.55)	-0.1 (-0.25 to 0.05)
Tokelau	7.47 (5.78 to 9.43)	3664.21 (2831.91 to 4621.71)	9.04 (6.98 to 11.45)	3585.11 (2768.46 to 4543.84)	NA
Tonga	285.07 (221.17 to 359.33)	3810.76 (2958.66 to 4798.57)	461.91 (358.63 to 580.91)	3686.87 (2862.19 to 4636.46)	-0.11 (-0.64 to 0.42)
Trinidad and Tobago	4311.68 (3380.36 to 5391.7)	3297.9 (2581.44 to 4128.92)	10096.77 (7867.43 to 12699.06)	3232.8 (2519.71 to 4061.79)	-0.06 (-0.22 to 0.09)
Tunisia	33180.27 (26080.26 to 41382.22)	4815.19 (3780.41 to 5991.93)	89161.54 (69893.9 to 111388.05)	4543.11 (3562.16 to 5670.92)	-0.21 (-0.26 to -0.15)
Turkey	238529.84 (187365.41 to 298098.99)	5022.4 (3950.08 to 6263.76)	665586.45 (519946.59 to 833568.13)	4708.69 (3677.87 to 5891.13)	-0.26 (-0.27 to -0.24)
Turkmenistan	9167.83 (7151.68 to 11444.04)	3669.2 (2863.31 to 4574.92)	17793.23 (13888.38 to 22348.75)	3487.36 (2728.27 to 4370.02)	-0.19 (-0.28 to -0.1)
Tuvalu	28.5 (22.1 to 35.92)	3821.66 (2974.83 to 4793.37)	49.82 (38.47 to 62.92)	3726.24 (2878.11 to 4695.91)	NA

Uganda	27102.79<br>(21319.7 to 33842.85)	3438.63<br>(2698.46 to 4289.29)	56981.2<br>(44559.7 to 71569.9)	3392.87<br>(2649.68 to 4254.78)	-0.06<br>(-0.12 to 0)
Ukraine	428476.14<br>(331888.31 to 542669.41)	3842.87<br>(2976.41 to 4868.25)	513894.98<br>(397472.73 to 651450.62)	3717.54<br>(2873.37 to 4710.81)	-0.13<br>(-0.15 to -0.12)
United Arab Emirates	1576.4<br>(1222.19 to 1994.88)	4149.15<br>(3218.69 to 5228.64)	8467.79<br>(6493.64 to 10782.24)	3740.38<br>(2877.39 to 4719.04)	-0.33<br>(-0.6 to -0.07)
United Kingdom	608798.88<br>(475051.56 to 762378)	3561.58<br>(2774.85 to 4466.42)	892122.41<br>(692500.05 to 1121274.64)	3401.71<br>(2633.86 to 4280.4)	-0.12<br>(-0.18 to -0.06)
United Republic of Tanzania	46703.89<br>(37126.18 to 57764.74)	3509.15<br>(2788.15 to 4331.98)	100920.08<br>(81037.71 to 124251.93)	3250.2<br>(2612.7 to 3994.71)	-0.26<br>(-0.31 to -0.21)
United States of America	2774801.5<br>(2178139.21 to 3482926.29)	4585.31<br>(3597.22 to 5758.02)	4752151.97<br>(3710337.66 to 5964869.67)	4390.64<br>(3429.7 to 5509.06)	-0.14<br>(-0.18 to -0.1)
United States Virgin Islands	368.34<br>(287.82 to 463.74)	3194.58<br>(2495.16 to 4015.27)	1021.76<br>(791.3 to 1291.87)	3109.62<br>(2406.33 to 3933.13)	-0.05<br>(-0.59 to 0.49)
Uruguay	25064.4<br>(19578.19 to 31388.53)	3605.66<br>(2815.3 to 4515.34)	38327.57<br>(29972.29 to 48296.65)	3400.94<br>(2659.53 to 4289.83)	-0.25<br>(-0.31 to -0.18)
Uzbekistan	62826.43<br>(49132.45 to 78926.37)	3482.89<br>(2723.23 to 4374.76)	107574.24<br>(83384.99 to 135629.98)	3460.64<br>(2686.78 to 4355.68)	-0.01<br>(-0.05 to 0.02)
Vanuatu	228.73<br>(175.42 to 290.76)	3546.62<br>(2734.35 to 4483.39)	623.08<br>(479.15 to 789.27)	3457.29<br>(2659.26 to 4367.05)	-0.01<br>(-0.79 to 0.78)

Venezuela (Bolivarian Republic of)	62132.17<br>(49027.99 to 77266.44)	4248.29<br>(3351.14 to 5281.68)	189949.09<br>(149492.58 to 237278.79)	4111.91<br>(3239.92 to 5131.71)	-0.11<br>(-0.14 to -0.08)
Viet Nam	237060.39<br>(186117.78 to 298266.32)	3936.74<br>(3088.88 to 4949.39)	504132<br>(392512.16 to 636320.21)	3708.72<br>(2889.37 to 4678.54)	-0.24<br>(-0.26 to -0.22)
Yemen	27103.57<br>(21236.18 to 33850.35)	4887.63<br>(3825.22 to 6084.04)	71522.3<br>(55947.5 to 89662.33)	4470.87<br>(3493.47 to 5593.69)	-0.3<br>(-0.36 to -0.24)
Zambia	10957.72<br>(8608.21 to 13759.2)	3348.91<br>(2626.73 to 4198.03)	24567.59<br>(19319.39 to 30684.03)	3378.25<br>(2655.4 to 4215.89)	0.06<br>(-0.04 to 0.16)
Zimbabwe	17213.94<br>(13497.38 to 21676.7)	3459.69<br>(2706.52 to 4356.72)	23466.35<br>(18440.63 to 29510.33)	3337.13<br>(2617.48 to 4193.15)	-0.11<br>(-0.19 to -0.03)

ASPR, age-standardized prevalence rate; 95% UI, 95% Uncertainty Intervals; 95% CI, 95% Confidence Interval.

S5 Table Trends in death cases and ASMR of Alzheimer's disease and other dementias from 1992 to 2021. (S5 Table expands on Table 3 by adding data from 21 regions.)

Characteristics	1992		2021		1992 to 2021
	Death cases, n (95% UI)	ASMR per 100 000, n (95% UI)	Death cases, n (95% UI)	ASMR per 100 000, n (95% UI)	Net Drift (%/year)
Global	707375.35 (170043.68 to 1959602.03)	147.57 (35.66 to 402.45)	1943723.27 (484157.15 to 5169896.02)	148.24 (37 to 392.52)	0 (-0.01 to 0.01)
Sex					
Male	212663.2 (49450.94 to 607770.61)	118.68 (27.68 to 333.47)	622675.85 (147603.8 to 1759059.42)	121.94 (28.99 to 340.71)	0.07 (0.05 to 0.09)
Female	494712.15 (120666.56 to 1347844.4)	162.8 (39.83 to 438.84)	1321047.42 (336176.84 to 3415771.88)	164.29 (41.79 to 425.37)	0.01 (0 to 0.03)
SDI					
High SDI	300099.7 (74295.19 to 800754.41)	159.42 (39.5 to 423.11)	717978.89 (186248.75 to 1812752.85)	154.51 (39.91 to 393.7)	-0.07 (-0.09 to -0.04)
High-middle SDI	181523.14 (43418.13 to 508584.31)	152.61 (36.58 to 421.4)	488133.01 (119543.42 to 1324387.31)	155.71 (38.15 to 420.95)	0 (-0.02 to 0.03)
Middle SDI	140604.87 (33077.66 to 400081.8)	139.93 (33 to 390.46)	490156.43 (118840.27 to 1339405.74)	144.69 (35.22 to 390.96)	-0.04 (-0.06 to -0.01)

Low-middle SDI	62607.42 (14398.64 to 180795.45)	105.03 (24.29 to 298.4)	187922.62 (44170.65 to 528620.28)	117.77 (27.85 to 327.01)	0.39 (0.36 to 0.43)
Low SDI	21809.89 (4930.35 to 63529.7)	115.12 (26.11 to 329.09)	57943.17 (13319.93 to 165598)	129.99 (30.16 to 364.6)	0.37 (0.32 to 0.43)
Region					
Andean Latin America	2458.85 (561.74 to 6954.44)	85.48 (19.55 to 241.12)	7625.07 (1753.64 to 20667.07)	82.83 (19.07 to 224.15)	-0.12 (-0.31 to 0.06)
Australasia	5426.7 (1313.53 to 14660.96)	144.04 (34.86 to 386.59)	15218.19 (3790.53 to 39442.19)	136.61 (33.97 to 355.86)	-0.13 (-0.29 to 0.02)
Caribbean	3550.79 (829.48 to 10030.69)	95.67 (22.31 to 268.71)	9023.06 (2099.31 to 24935.07)	93.1 (21.67 to 258.51)	-0.07 (-0.24 to 0.09)
Central Asia	7559.7 (1766.78 to 21110.19)	122.36 (28.67 to 340.2)	10932.25 (2565.24 to 30777.09)	118.21 (27.93 to 329.22)	-0.11 (-0.23 to 0.02)
Central Europe	23292.55 (5426.91 to 66150.58)	122.31 (28.49 to 343.76)	49733.55 (11874.82 to 137224.9)	120.52 (28.72 to 332.65)	-0.03 (-0.1 to 0.04)
Central Latin America	10638.68 (2490.35 to 29932.01)	100.89 (23.69 to 280.47)	38507.05 (9216.15 to 104683.44)	99.15 (23.75 to 269.89)	-0.04 (-0.12 to 0.05)
Central Sub-Saharan Africa	2691.95 (612.68 to 7765.19)	179.77 (41.6 to 500.94)	7996.98 (1821.45 to 22598.18)	205.65 (48.15 to 566.13)	0.5 (0.34 to 0.66)

East Asia	132245.38<br>(31273.14 to 377110.6)	180.48<br>(42.74 to 505.44)	504860.84<br>(122452.93 to 1385295.14)	179.2<br>(43.6 to 486.32)	-0.19<br>(-0.21 to -0.16)
Eastern Europe	44989.35<br>(10455.75 to 127449.86)	126.7<br>(29.45 to 355.82)	75073.02<br>(17735.52 to 207807.98)	123.61<br>(29.25 to 341.96)	-0.01<br>(-0.07 to 0.04)
Eastern Sub-Saharan Africa	8561.03<br>(1947.23 to 24464.48)	142.35<br>(32.61 to 397.22)	23081.83<br>(5400.48 to 63754.97)	160.14<br>(37.97 to 434.33)	0.36<br>(0.27 to 0.46)
High-income Asia Pacifc	46928.43<br>(11733.19 to 124565.01)	162.99<br>(41.04 to 426.12)	199893.95<br>(54575.75 to 477193)	156.77<br>(42.37 to 381.48)	-0.03<br>(-0.09 to 0.02)
High-income North America	113218.75<br>(28209.93 to 297168.1)	171.34<br>(42.64 to 449.3)	216216.87<br>(54832.25 to 554090.77)	166.49<br>(42.15 to 428.85)	-0.13<br>(-0.17 to -0.09)
North Africa and Middle East	27405.95<br>(6493.6 to 76635.47)	163.33<br>(39.03 to 447.38)	73205.52<br>(17392.78 to 201418.03)	150.94<br>(36.15 to 409.58)	-0.32<br>(-0.37 to -0.27)
Oceania	271.32<br>(61.15 to 784.61)	134.81<br>(30.69 to 383.66)	721.34<br>(165.36 to 2063.83)	124.02<br>(28.98 to 350.33)	-0.27<br>(-0.77 to 0.23)
South Asia	45045.19<br>(10003.75 to 132304.32)	84.16<br>(18.58 to 244.32)	163874.29<br>(37829.64 to 464463.46)	101.23<br>(23.44 to 283.78)	0.61<br>(0.57 to 0.65)
Southeast Asia	32588.79<br>(7430.48 to 92856.21)	124.4<br>(28.52 to 347.47)	97451.54<br>(23026.34 to 269439.19)	133.29<br>(31.71 to 363.03)	0.19<br>(0.14 to 0.23)
Southern Latin America	7961.05<br>(1897.61 to 22105.3)	121.14<br>(28.94 to 333.7)	18726.98<br>(4578.64 to 49941.48)	118.19<br>(28.88 to 315.54)	-0.03<br>(-0.15 to 0.1)

Southern Sub-Saharan Africa	4188.78 (975.47 to 11676.51)	129.23 (30.07 to 357.97)	7787.48 (1814.66 to 22158.75)	133.71 (31.29 to 374.66)	0.09 (-0.06 to 0.24)
Tropical Latin America	17429.17 (4249.61 to 47609.05)	164.47 (40.59 to 439.54)	64913.18 (16503.18 to 168845.5)	160.55 (40.87 to 417.23)	-0.05 (-0.11 to 0.02)
Western Europe	162056.06 (40147.24 to 432764.62)	157.71 (39.01 to 419.24)	339096.67 (87576.18 to 856946.46)	152.09 (39.08 to 387.48)	-0.1 (-0.13 to -0.06)
Western Sub-Saharan Africa	8866.9 (2016.05 to 25347.48)	106.15 (24.11 to 300.11)	19783.61 (4420.15 to 57447.83)	113.36 (25.56 to 323.86)	0.31 (0.22 to 0.41)

SDI, socio-demographic index; ASMR, age-standardized mortality rate; 95% UI, 95% Uncertainty Interval; 95% CI, 95% Confidence Interval.

S6 Table Trends in death cases and ASMR of Alzheimer's disease and other dementias from 1992 to 2021 across countries and territories.

Location	Death cases 1992, (95%UI)	ASMR 1992, (95%UI)	Death cases 2021, (95%UI)	ASMR 2021, (95%UI)	Net Drift (%/year)
Afghanistan	1276.47 (294.13 to 3714.54)	206.75 (48.61 to 586.35)	1591.07 (370.46 to 4374.41)	195.98 (46.32 to 531.34)	-0.15 (-0.45 to 0.14)
Albania	317.87 (73.42 to 876.2)	125.89 (29.14 to 345.05)	788.57 (177.06 to 2255.68)	122.65 (27.46 to 347.47)	-0.17 (-0.7 to 0.36)
Algeria	1682.39 (388.11 to 4847.65)	163.77 (37.99 to 466.43)	5543.47 (1267.07 to 15269.3)	154.32 (35.33 to 423.68)	-0.17 (-0.42 to 0.09)
American Samoa	2.6 (0.6 to 7.35)	141.71 (33.02 to 390.12)	6.49 (1.48 to 18.17)	132.32 (30.22 to 365.45)	-1.02 (-7.26 to 5.63)
Andorra	12.03 (2.84 to 31.58)	150.5 (35.99 to 390.72)	46.29 (11.63 to 121.79)	142.03 (35.3 to 378.99)	-0.33 (-4.29 to 3.8)
Angola	423.22 (95.56 to 1228.22)	167.86 (38.15 to 474.03)	1543.28 (352.23 to 4444.24)	196.85 (46.31 to 552.77)	0.55 (0.16 to 0.94)
Antigua and Barbuda	9.87 (2.25 to 28.03)	96.67 (22 to 274.83)	12.25 (2.84 to 35.09)	92.91 (21.52 to 265.15)	-0.72 (-5.14 to 3.9)
Argentina	5611.72 (1330.82 to 15617.69)	122.85 (29.19 to 338.9)	11978.37 (2926.91 to 31976.66)	119.07 (29.08 to 317.98)	-0.02 (-0.18 to 0.13)
Armenia	392.64 (90.53 to 1114.5)	118.91 (27.57 to 335.12)	900 (214.03 to 2427.89)	125.79 (29.96 to 338.62)	0.3 (-0.22 to 0.82)

Australia	4449.16 (1077.65 to 12014.63)	142.02 (34.4 to 381.11)	12818.87 (3190.22 to 33106.95)	134.93 (33.52 to 350.36)	-0.11 (-0.28 to 0.06)
Austria	3156.83 (757.91 to 8656.4)	149.82 (35.77 to 409.65)	6063.91 (1528.23 to 15640.48)	144.12 (36.17 to 374.6)	-0.07 (-0.33 to 0.19)
Azerbaijan	774.54 (181.61 to 2151.9)	124.75 (29.22 to 344.71)	1275.68 (295.16 to 3662.24)	121 (28.32 to 343.4)	-0.09 (-0.47 to 0.28)
Bahamas	19.88 (4.67 to 56.37)	95.56 (22.5 to 269.03)	47.66 (10.78 to 135.12)	92.81 (21.09 to 261.97)	-0.29 (-2.36 to 1.83)
Bahrain	16.09 (3.7 to 46.6)	172.36 (40.04 to 486.15)	69.24 (15.49 to 200.06)	151.06 (34.51 to 429.34)	-1.04 (-3.53 to 1.51)
Bangladesh	4851.91 (1088.87 to 13995.47)	91.33 (20.44 to 261.45)	16422.58 (3674.54 to 48884.78)	101.64 (22.83 to 301.5)	0.23 (0.1 to 0.35)
Barbados	49.9 (11.54 to 142.64)	92.79 (21.33 to 264.81)	81.46 (18.34 to 226.5)	94.09 (21.19 to 261.35)	0.01 (-1.67 to 1.71)
Belarus	2409.85 (560.33 to 6789.1)	123.78 (28.83 to 347.25)	3406.29 (792.23 to 9570.26)	121.21 (28.21 to 340.16)	-0.02 (-0.27 to 0.23)
Belgium	4695.82 (1151.12 to 12420.2)	173.14 (42.44 to 456.68)	8813.28 (2308.82 to 21987.55)	156.15 (40.67 to 393.82)	-0.2 (-0.42 to 0.01)
Belize	15.64 (3.69 to 42.3)	94.81 (22.36 to 257.13)	37.96 (8.9 to 103.24)	95.33 (22.43 to 258.44)	-0.13 (-2.8 to 2.61)

Benin	248.7<br>(56.2 to 706.26)	106.13<br>(23.96 to 301.28)	542.91<br>(122.66 to 1553.18)	108.58<br>(24.63 to 307.21)	-0.06<br>(-0.64 to 0.53)
Bermuda	8.54<br>(1.97 to 23.97)	98.86<br>(22.79 to 276.05)	27.22<br>(6.53 to 72.3)	96.87<br>(23.21 to 258.27)	-0.82<br>(-4.86 to 3.39)
Bhutan	19.2<br>(4.13 to 54.77)	92.28<br>(19.84 to 261.42)	94.33<br>(21.38 to 269.36)	115.58<br>(26.22 to 328.37)	0.64<br>(-1.11 to 2.42)
Bolivia (Plurinational State of)	308.33<br>(69.85 to 882.88)	91.41<br>(20.64 to 259.4)	924.64<br>(209.76 to 2570.72)	92.07<br>(20.99 to 253.04)	0.03<br>(-0.44 to 0.5)
Bosnia and Herzegovina	560.72<br>(127.97 to 1565.01)	120.2<br>(27.72 to 330.93)	1198.16<br>(275.26 to 3220.24)	115.27<br>(26.58 to 307.09)	-0.17<br>(-0.59 to 0.25)
Botswana	59.99<br>(12.95 to 176.84)	137.97<br>(30.17 to 406.38)	170.19<br>(39.07 to 480.07)	133.03<br>(30.85 to 366.45)	-0.19<br>(-1.24 to 0.88)
Brazil	16928.8<br>(4128.35 to 46220.78)	165.18<br>(40.8 to 440.84)	63590.81<br>(16179.46 to 165451.81)	160.76<br>(40.95 to 417.89)	-0.05<br>(-0.12 to 0.01)
Brunei Darussalam	12.71<br>(2.95 to 36.28)	131.85<br>(30.9 to 371.79)	33.17<br>(7.69 to 90.98)	137.1<br>(32.57 to 368.79)	0.04<br>(-3 to 3.18)
Bulgaria	1474.09<br>(341.23 to 4203.63)	117.44<br>(26.59 to 334.92)	2780.8<br>(650.76 to 7874.48)	118.03<br>(27.36 to 335.16)	-0.07<br>(-0.42 to 0.27)
Burkina Faso	485.74<br>(111.31 to 1376.52)	129.08<br>(29.54 to 361.55)	1008.62<br>(227.06 to 2877.1)	119.41<br>(27.22 to 334.44)	-0.37<br>(-0.79 to 0.04)

Burundi	273.32<br>(61.09 to 772.71)	133.48<br>(29.93 to 368.68)	555.96<br>(124.41 to 1613.17)	147.59<br>(33.62 to 419.77)	0.37<br>(-0.2 to 0.94)
Cabo Verde	42<br>(9.63 to 117.03)	97.94<br>(22.5 to 272.65)	72.41<br>(16.67 to 198.23)	103.28<br>(23.77 to 282.79)	0.32<br>(-1.55 to 2.23)
Cambodia	488.27<br>(111.49 to 1388.28)	125.89<br>(28.85 to 354.54)	1670.82<br>(377.4 to 4803.88)	151.53<br>(34.64 to 428.25)	0.7<br>(0.34 to 1.07)
Cameroon	483.97<br>(111.71 to 1367.82)	120.51<br>(27.88 to 337.54)	1159.41<br>(250.98 to 3489.61)	113.34<br>(25.01 to 336.1)	-0.24<br>(-0.63 to 0.16)
Canada	7163.48<br>(1743.93 to 19402.1)	129.85<br>(31.61 to 350.5)	18607.24<br>(4672.8 to 48242.87)	124.55<br>(31.24 to 324.68)	-0.18<br>(-0.32 to -0.04)
Central African Republic	128.78<br>(28.95 to 357.18)	192.38<br>(44.22 to 519.42)	218.13<br>(47.03 to 609.48)	188.49<br>(41.48 to 512)	-0.04<br>(-0.84 to 0.76)
Chad	309.56<br>(68.16 to 881.21)	101.19<br>(22.06 to 286.36)	510.74<br>(111.43 to 1459.23)	104.51<br>(23 to 293.71)	0.18<br>(-0.36 to 0.72)
Chile	1558.66<br>(373.41 to 4269.49)	113.76<br>(27.27 to 309.57)	5178.16<br>(1270.47 to 13673.4)	114.37<br>(28.03 to 302.42)	-0.04<br>(-0.29 to 0.22)
China	128370.02<br>(30364.13 to 366236.61)	183.54<br>(43.44 to 514.15)	489054.9<br>(118825.5 to 1341700.19)	181.67<br>(44.29 to 492.83)	-0.19<br>(-0.22 to -0.17)
Colombia	2454.52<br>(582.66 to 6813.18)	106.19<br>(25.32 to 292.27)	10990.57<br>(2712.21 to 28783.06)	105.11<br>(25.68 to 278.48)	0.04<br>(-0.14 to 0.21)

Comoros	23.43<br>(5.27 to 64.53)	148.42<br>(34.05 to 398.49)	81.98<br>(18.99 to 229.91)	165.92<br>(38.9 to 458.08)	0.17<br>(-1.5 to 1.87)
Congo	144.68<br>(33.37 to 400.74)	204.96<br>(47.89 to 555.57)	372.18<br>(84.4 to 1029.5)	203.23<br>(46.61 to 545.79)	0.06<br>(-0.78 to 0.9)
Cook Islands	1.9<br>(0.44 to 5.42)	146.58<br>(34.2 to 410.35)	5.11<br>(1.2 to 13.87)	128.97<br>(30.45 to 348.78)	0.08<br>(-7.6 to 8.39)
Costa Rica	306.05<br>(72.4 to 846.17)	108.21<br>(25.63 to 298.45)	1074.21<br>(264.3 to 2803.94)	106.2<br>(26 to 280.18)	-0.01<br>(-0.56 to 0.53)
Coted'Ivoire	312.1<br>(71.39 to 872.22)	113.48<br>(26.27 to 311.26)	971.33<br>(211.14 to 2828.23)	112.47<br>(24.76 to 322.51)	0.04<br>(-0.41 to 0.49)
Croatia	945.81<br>(217.54 to 2698.44)	123.08<br>(28.32 to 348.93)	2045.32<br>(476.93 to 5636.89)	119.82<br>(27.86 to 330.7)	-0.11<br>(-0.46 to 0.25)
Cuba	1479.56<br>(347.09 to 4178.54)	93.29<br>(21.77 to 262.77)	3480.99<br>(804.8 to 9868.13)	90.66<br>(21.05 to 257.88)	-0.08<br>(-0.35 to 0.2)
Cyprus	129.43<br>(30.07 to 372.66)	170.35<br>(38.6 to 489.21)	438.52<br>(104.01 to 1217.65)	152.25<br>(35.52 to 424.82)	-0.79<br>(-1.79 to 0.22)
Czechia	2439.95<br>(569.81 to 6943.68)	120.28<br>(28.03 to 339.72)	4886.33<br>(1176.97 to 13272.12)	119.98<br>(28.86 to 326.17)	0.05<br>(-0.19 to 0.28)
Democratic People's Republic of Korea	2035.66<br>(441.15 to 5816.61)	137.27<br>(29.75 to 387.46)	5785.23<br>(1301.93 to 16198.88)	142.56<br>(32.13 to 395.26)	0.24<br>(0.04 to 0.44)

Democratic Republic of the Congo	1846.76<br>(413.83 to 5327.02)	178.22<br>(40.6 to 495.69)	5599.2<br>(1249.6 to 15721.79)	208.87<br>(48.04 to 571.53)	0.61<br>(0.42 to 0.81)
Denmark	2139.9<br>(514.11 to 5907.32)	138.15<br>(33.08 to 381.08)	3505.15<br>(859.91 to 9163.44)	142.26<br>(34.85 to 372.88)	-0.04<br>(-0.36 to 0.27)
Djibouti	13.56<br>(3.05 to 39.65)	155.09<br>(35.28 to 439.78)	66.73<br>(14.54 to 187.38)	169.46<br>(38.03 to 463.76)	0.09<br>(-1.83 to 2.05)
Dominica	8.64<br>(1.97 to 24.87)	98.09<br>(22.28 to 281.43)	11.35<br>(2.64 to 32.36)	96.93<br>(22.67 to 275.01)	0.38<br>(-4.12 to 5.1)
Dominican Republic	456.35<br>(107.46 to 1261.53)	97.83<br>(22.94 to 268.95)	1517.65<br>(345.43 to 4169.9)	96.49<br>(21.97 to 265.07)	-0.02<br>(-0.43 to 0.4)
Ecuador	617.56<br>(143.24 to 1756.12)	85.36<br>(19.69 to 242.13)	1938.4<br>(424.04 to 5472.81)	82.1<br>(17.9 to 231.96)	-0.09<br>(-0.45 to 0.27)
Egypt	3149.76<br>(718.54 to 9072.51)	157.54<br>(36.12 to 447.63)	6417.76<br>(1457.76 to 18410.83)	143.66<br>(33.31 to 401.36)	-0.37<br>(-0.53 to -0.21)
El Salvador	561.64<br>(134.47 to 1523.26)	109.4<br>(26.21 to 297.81)	1402.15<br>(342.12 to 3606.69)	107.61<br>(25.94 to 280.03)	-0.11<br>(-0.56 to 0.35)
Equatorial Guinea	24.65<br>(5.48 to 70.95)	175.26<br>(39.42 to 493.48)	78.57<br>(17.27 to 220.35)	194.78<br>(43.76 to 537.04)	0.04<br>(-1.58 to 1.68)
Eritrea	74.17<br>(16.79 to 213.76)	143.88<br>(33.07 to 404.32)	297.21<br>(65.69 to 856.03)	177.02<br>(39.62 to 491.86)	0.48<br>(-0.44 to 1.4)

Estonia	364.08<br>(84.28 to 1039.13)	122.87<br>(28.42 to 348.93)	783.36<br>(187.59 to 2094.63)	129.5<br>(30.85 to 347.49)	0.1<br>(-0.56 to 0.76)
Eswatini	33.42<br>(7.53 to 95.03)	138.17<br>(31.28 to 391.73)	53.4<br>(11.44 to 153.1)	137.62<br>(30.64 to 381.05)	-0.05<br>(-1.57 to 1.5)
Ethiopia	1995.3<br>(448.8 to 5864.17)	155.99<br>(35.67 to 443.91)	7338.45<br>(1738.12 to 19927.36)	168.9<br>(40.5 to 449.82)	0.23<br>(0.02 to 0.43)
Fiji	36.53<br>(8.26 to 106.92)	140.28<br>(31.97 to 403.94)	75.64<br>(16.97 to 210.02)	132.74<br>(30.34 to 367.07)	-0.07<br>(-1.56 to 1.46)
Finland	2035.8<br>(498.18 to 5409.39)	166.03<br>(40.67 to 439.42)	4584.68<br>(1165.41 to 11611.2)	154.18<br>(39.08 to 393.05)	-0.18<br>(-0.48 to 0.13)
France	21682.51<br>(5240.46 to 58463.76)	135.2<br>(32.55 to 364.48)	46513.25<br>(11698.26 to 118623.31)	127.92<br>(32.16 to 328.69)	-0.16<br>(-0.26 to -0.06)
Gabon	123.85<br>(29.25 to 330.39)	208.44<br>(49.56 to 544.67)	185.63<br>(42.77 to 512.77)	208.91<br>(48.87 to 565.48)	-0.11<br>(-1 to 0.78)
Gambia	35.46<br>(7.92 to 101.89)	117<br>(26.33 to 328.98)	113.46<br>(25.28 to 332.63)	118.64<br>(26.76 to 342.68)	0.15<br>(-1.25 to 1.57)
Georgia	1066.14<br>(250.07 to 2980.53)	120.76<br>(28.34 to 336.02)	1563.69<br>(383.37 to 4144.98)	124.34<br>(30.25 to 335.7)	0.17<br>(-0.21 to 0.55)
Germany	38958.92<br>(9938.76 to 101128.43)	172.92<br>(44.19 to 446.54)	78481.37<br>(20731.71 to 195161.02)	172.57<br>(45.26 to 432.28)	-0.03<br>(-0.11 to 0.04)

Ghana	516.56<br>(116.31 to 1506.34)	99.19<br>(22.52 to 285.43)	1572.14<br>(350.57 to 4628.76)	114.12<br>(25.67 to 332.05)	0.51<br>(0.15 to 0.87)
Greece	3430.41<br>(820.35 to 9519.89)	146.06<br>(34.84 to 402.45)	8914.48<br>(2192.35 to 23401.91)	142.89<br>(35 to 377.6)	-0.05<br>(-0.28 to 0.18)
Greenland	3.7<br>(0.85 to 10.76)	156.44<br>(35.84 to 444.36)	9.36<br>(2.2 to 25.13)	151.33<br>(36.01 to 397.47)	0.16<br>(-5.38 to 6.02)
Grenada	14.57<br>(3.33 to 40.49)	94.87<br>(21.69 to 264.39)	12.42<br>(2.82 to 35.6)	98.61<br>(22.56 to 279.47)	-0.18<br>(-4.45 to 4.27)
Guam	8.26<br>(1.94 to 23.39)	133.92<br>(31.77 to 370.15)	47.62<br>(13.3 to 110.69)	107.7<br>(29.18 to 260.07)	-0.08<br>(-3.06 to 2.99)
Guatemala	339.33<br>(77.09 to 984.68)	111.49<br>(25.1 to 320.47)	1521.24<br>(351.86 to 4205.95)	107.65<br>(24.82 to 298.03)	-0.11<br>(-0.57 to 0.35)
Guinea	375.94<br>(84.24 to 1058.33)	100.36<br>(22.44 to 281.69)	644.79<br>(139.38 to 1837.63)	108.9<br>(23.66 to 306.83)	0.2<br>(-0.28 to 0.69)
Guinea-Bissau	32.04<br>(7.05 to 92.45)	111.49<br>(24.61 to 318.02)	53.07<br>(11.46 to 157.15)	116.17<br>(25.44 to 341.57)	0.06<br>(-1.49 to 1.63)
Guyana	37.2<br>(8.58 to 106.53)	88.33<br>(20.4 to 251.49)	62.91<br>(14.02 to 178.43)	91.57<br>(20.38 to 257.77)	-0.25<br>(-1.86 to 1.38)
Haiti	265.02<br>(59.59 to 765.79)	100.24<br>(22.72 to 287.96)	579.66<br>(122.44 to 1711.76)	97.05<br>(20.48 to 283.25)	-0.09<br>(-0.61 to 0.44)

Honduras	300.57<br>(68.91 to 846.33)	122.06<br>(28.04 to 341.24)	901.02<br>(206.56 to 2578.94)	135.21<br>(31.22 to 382.44)	0.36<br>(-0.11 to 0.85)
Hungary	2429.63<br>(562.4 to 6923.05)	122.03<br>(28.07 to 344.69)	4551.06<br>(1077.02 to 12402.18)	118.39<br>(27.95 to 323.12)	0<br>(-0.24 to 0.24)
Iceland	81.99<br>(20.06 to 217.7)	145.87<br>(35.64 to 388.03)	182.94<br>(46.48 to 458.32)	143.37<br>(36.29 to 362.48)	0.2<br>(-1.34 to 1.76)
India	32976.65<br>(7287.32 to 97251.85)	79.78<br>(17.55 to 232.07)	132098.67<br>(30587.14 to 370232.97)	99.96<br>(23.25 to 277.07)	0.76<br>(0.71 to 0.81)
Indonesia	9924.32<br>(2181.48 to 28330.19)	108.38<br>(23.87 to 306.43)	25906.72<br>(5913.59 to 75391.7)	135.23<br>(31.18 to 389.19)	0.48<br>(0.4 to 0.56)
Iran (Islamic Republic of)	3522.2<br>(837.15 to 9808.02)	160.93<br>(38.72 to 438.68)	14841.36<br>(3554.07 to 40114.84)	148.38<br>(35.69 to 398.65)	-0.27<br>(-0.4 to -0.13)
Iraq	1778.13<br>(429.22 to 4796.18)	154.25<br>(37.32 to 415.51)	3277.94<br>(754.61 to 9085.54)	151.9<br>(35.5 to 417.03)	-0.29<br>(-0.53 to -0.05)
Ireland	912.43<br>(216.57 to 2542.28)	148.91<br>(35.25 to 412.08)	2111.39<br>(530.93 to 5383.78)	142.09<br>(35.69 to 362.73)	-0.09<br>(-0.5 to 0.33)
Israel	1065.61<br>(257.73 to 2922.8)	144.51<br>(34.84 to 394.02)	3603.46<br>(911.44 to 9172.69)	143.25<br>(36.11 to 366.42)	-0.03<br>(-0.38 to 0.32)
Italy	27736.34<br>(6876.3 to 73275.61)	185.52<br>(46.08 to 486.46)	64673.34<br>(17071.21 to 160725.79)	175.47<br>(45.99 to 440.97)	-0.18<br>(-0.26 to -0.1)

Jamaica	327.26 (77.72 to 911.1)	95.71 (22.71 to 266.66)	645.01 (154.72 to 1733.55)	94.35 (22.37 to 259.17)	-0.09 (-0.75 to 0.57)
Japan	41419.38 (10405.15 to 109173.54)	160.03 (40.44 to 416.35)	172633.28 (47424.31 to 409607.51)	155.2 (42.1 to 377.19)	-0.01 (-0.07 to 0.04)
Jordan	190.08 (45.79 to 521.86)	152.09 (36.99 to 411.98)	903.87 (210.54 to 2562.22)	137.75 (32.7 to 384.48)	-0.48 (-1.05 to 0.09)
Kazakhstan	1945.13 (450.53 to 5455.28)	124.72 (28.98 to 347.72)	2118.84 (481.81 to 6148.12)	114.52 (26.03 to 329.68)	-0.38 (-0.64 to -0.12)
Kenya	1198.06 (269.28 to 3317.83)	142.15 (32.33 to 389.62)	3112.43 (698.49 to 8232.82)	169.16 (38.68 to 435.74)	0.57 (0.32 to 0.82)
Kiribati	3.89 (0.86 to 11.14)	141.97 (30.83 to 400.9)	7.77 (1.71 to 22.14)	161.32 (35.7 to 450.54)	0.93 (-4.17 to 6.3)
Kuwait	89.73 (22.45 to 235.13)	150.89 (38.3 to 389.04)	468.69 (116.65 to 1242.48)	143.64 (35.81 to 381.53)	-0.42 (-1.26 to 0.42)
Kyrgyzstan	495.97 (115.41 to 1373.51)	126.31 (29.43 to 347.31)	626.74 (148.86 to 1720.07)	118.35 (28.2 to 322.6)	-0.11 (-0.63 to 0.4)
Lao People's Democratic Republic	186.43 (41.08 to 533.47)	116.5 (25.88 to 328.35)	540.43 (120.4 to 1591.84)	127.11 (28.73 to 370.16)	0.27 (-0.34 to 0.89)
Latvia	676.22 (156.92 to 1884.32)	122.76 (28.47 to 341.14)	1051.07 (248.05 to 2900.57)	122.53 (28.83 to 338.53)	-0.03 (-0.53 to 0.47)

Lebanon	360.69<br>(84.3 to 993.7)	144.68<br>(34.12 to 394.79)	1651.1<br>(400.4 to 4473.07)	140.49<br>(34 to 381.26)	-0.13<br>(-0.61 to 0.35)
Lesotho	124.67<br>(27.46 to 342.84)	130.64<br>(28.48 to 357.98)	117.96<br>(26.4 to 346.23)	138.02<br>(31.57 to 393.64)	0.36<br>(-0.56 to 1.3)
Liberia	114.87<br>(25.6 to 333.96)	103.43<br>(23.07 to 295.04)	195.04<br>(41.34 to 572.38)	105.17<br>(22.51 to 303.91)	0.09<br>(-0.81 to 0.99)
Libya	475.63<br>(115.83 to 1214.12)	177.21<br>(43.25 to 452.23)	980.67<br>(227.84 to 2691.22)	165.27<br>(38.63 to 451.28)	-0.32<br>(-0.79 to 0.15)
Lithuania	884.82<br>(207.23 to 2505.39)	121.83<br>(28.54 to 344.05)	1543.08<br>(364.83 to 4282.9)	123.87<br>(29.17 to 344.93)	-0.03<br>(-0.46 to 0.4)
Luxembourg	91.33<br>(20.84 to 257.52)	105.67<br>(23.99 to 296.72)	232.11<br>(56.38 to 608.15)	104.48<br>(25.32 to 275.34)	-0.11<br>(-1.38 to 1.18)
Madagascar	505.95<br>(115.19 to 1466.97)	110.16<br>(25.11 to 317.19)	842.11<br>(187.43 to 2411.99)	116.27<br>(26.29 to 324.76)	0.15<br>(-0.25 to 0.57)
Malawi	424.57<br>(96.61 to 1216.99)	139.93<br>(32.02 to 393.06)	946.86<br>(211.93 to 2700.44)	159.49<br>(36.24 to 447.68)	0.44<br>(0.01 to 0.87)
Malaysia	1770.15<br>(430.85 to 4764.28)	139.62<br>(34.08 to 374.37)	4679.42<br>(1107.71 to 13074.52)	143.61<br>(34.18 to 396.89)	-0.03<br>(-0.24 to 0.19)
Maldives	7.27<br>(1.62 to 21.25)	106.63<br>(24.08 to 305.21)	46.71<br>(11.14 to 130.19)	115.04<br>(27.63 to 319.89)	0.44<br>(-2.54 to 3.5)

Mali	386.74<br>(86.79 to 1089.3)	128.9<br>(29.01 to 357.78)	889.5<br>(198.64 to 2561.86)	128.66<br>(29.23 to 361.84)	0.1<br>(-0.33 to 0.53)
Malta	92.83<br>(22.42 to 252.11)	149.65<br>(36.35 to 401.39)	290.87<br>(74.68 to 736.75)	141.55<br>(36.24 to 360.05)	-0.34<br>(-1.53 to 0.85)
Marshall Islands	1.9<br>(0.43 to 5.51)	153.9<br>(34.88 to 440.64)	2.69<br>(0.59 to 7.86)	142.29<br>(31.97 to 406.43)	-1.75<br>(-11.13 to 8.62)
Mauritania	111.88<br>(25.52 to 318.98)	110.84<br>(25.23 to 312.82)	263.01<br>(56.65 to 779.45)	112.31<br>(24.27 to 329.51)	0.04<br>(-0.82 to 0.9)
Mauritius	102.85<br>(23.95 to 287.28)	133.92<br>(31.41 to 367.89)	321.29<br>(76.77 to 884.1)	119.52<br>(28.66 to 327.66)	-0.08<br>(-0.94 to 0.79)
Mexico	4562.98<br>(1052.01 to 13096.59)	88.54<br>(20.34 to 251.97)	15431.96<br>(3607.14 to 44290.16)	87.11<br>(20.39 to 249.07)	-0.08<br>(-0.21 to 0.05)
Micronesia (Federated States of)	7.09<br>(1.62 to 20.48)	156.38<br>(35.89 to 444.89)	8.56<br>(1.91 to 24.71)	154.48<br>(35.14 to 439.8)	0.24<br>(-3.94 to 4.61)
Monaco	23.98<br>(5.67 to 65.19)	152.52<br>(35.86 to 416.15)	37.99<br>(9.45 to 93.85)	160.59<br>(39.71 to 401.04)	0.05<br>(-3.91 to 4.18)
Mongolia	148.69<br>(34.63 to 421.91)	136.97<br>(32.1 to 384.1)	244.9<br>(56.58 to 694.8)	127.66<br>(30.08 to 353.64)	-0.18<br>(-1.04 to 0.68)
Montenegro	112.35<br>(26.23 to 310.33)	119.93<br>(28.07 to 329.56)	150.85<br>(34.73 to 422.34)	120.58<br>(27.77 to 334.76)	-0.06<br>(-1.08 to 0.97)

Morocco	2766.03<br>(652.08 to 7730.14)	155.01<br>(36.66 to 430.61)	6042.8<br>(1398.53 to 16901.79)	153.97<br>(35.75 to 427.12)	0.01<br>(-0.17 to 0.19)
Mozambique	747.56<br>(174.11 to 2117.19)	156.44<br>(36.72 to 435.58)	1419.11<br>(317.99 to 4156.95)	175.37<br>(39.71 to 503.82)	0.49<br>(0.16 to 0.82)
Myanmar	2314.96<br>(520.67 to 6794.01)	113.62<br>(25.58 to 328.34)	6793.14<br>(1532.8 to 19695.17)	126.57<br>(28.7 to 364.51)	0.28<br>(0.1 to 0.45)
Namibia	67.81<br>(15.44 to 190.86)	128.98<br>(29.39 to 358.79)	173.24<br>(38.36 to 504.93)	142.88<br>(31.95 to 413.22)	0.36<br>(-0.65 to 1.37)
Nauru	0.41<br>(0.09 to 1.23)	148.24<br>(33.8 to 429.93)	0.59<br>(0.13 to 1.72)	148.3<br>(33.04 to 430.9)	NA
Nepal	687.43<br>(151.86 to 1997.5)	82.26<br>(18.19 to 237.09)	2472.62<br>(546.92 to 7144.52)	101.96<br>(22.73 to 292.49)	0.74<br>(0.43 to 1.05)
Netherlands	5947.06<br>(1501.22 to 15436.97)	167.58<br>(42.27 to 434.16)	11634.49<br>(2962.49 to 29841.17)	164.83<br>(41.89 to 423.65)	0.09<br>(-0.1 to 0.27)
New Zealand	977.54<br>(237.19 to 2636.05)	154.05<br>(37.37 to 412.4)	2399.32<br>(606.72 to 6256.96)	146.37<br>(36.98 to 382.65)	-0.19<br>(-0.56 to 0.18)
Nicaragua	229.88<br>(55.13 to 619.02)	107.77<br>(25.95 to 288.18)	661.83<br>(159.85 to 1776.39)	103.45<br>(24.93 to 277.01)	-0.1<br>(-0.69 to 0.5)
Niger	224.14<br>(50.62 to 636.01)	109.62<br>(24.64 to 305.69)	714.95<br>(156.46 to 2088.55)	113.82<br>(25.42 to 324.32)	0.2<br>(-0.33 to 0.73)

Nigeria	4484.01 (1027.84 to 12820.37)	102.4 (23.42 to 289.01)	9422.3 (2127.73 to 27149.34)	111.93 (25.56 to 317.24)	0.5 (0.36 to 0.64)
Niue	0.66 (0.15 to 1.86)	163.25 (37.36 to 456.35)	0.45 (0.11 to 1.24)	146.97 (34.97 to 400.92)	NA
North Macedonia	271.76 (62.9 to 767.25)	115.46 (26.84 to 324.52)	371.29 (84.98 to 1061.5)	115.87 (26.44 to 329.54)	-0.07 (-0.72 to 0.59)
Northern Mariana Islands	1.35 (0.31 to 3.88)	136.67 (31.54 to 382.01)	5.54 (1.29 to 15.6)	134.35 (31.77 to 372.54)	0.01 (-8.01 to 8.72)
Norway	2173.19 (532.35 to 5811.36)	156.71 (38.31 to 419.23)	3236.36 (807.08 to 8364.99)	146.67 (36.47 to 381.68)	-0.32 (-0.66 to 0.02)
Oman	108.14 (25.32 to 292.78)	166.26 (39.8 to 439.43)	222.54 (54.53 to 636.59)	153.96 (38.32 to 426.21)	-0.32 (-1.2 to 0.57)
Pakistan	6510 (1438.41 to 18812.23)	102.8 (22.49 to 296.42)	12786.08 (2879.63 to 35886.4)	115.35 (26.03 to 319.91)	0.24 (0.13 to 0.36)
Palau	1.3 (0.29 to 3.71)	144.59 (32.58 to 409.01)	2.17 (0.48 to 6.33)	127.75 (28.53 to 371.78)	-2.28 (-11.61 to 8.03)
Palestine	150.02 (34.33 to 436.63)	157.66 (36.12 to 454.75)	325.78 (75.15 to 937.91)	145.51 (33.61 to 410.42)	-0.32 (-1.09 to 0.45)
Panama	242.16 (57.62 to 667.05)	103.2 (24.62 to 283.3)	854.72 (210.6 to 2209.93)	103.61 (25.39 to 269.29)	0 (-0.61 to 0.62)

Papua New Guinea	145.53<br>(31.82 to 413.7)	125.05<br>(27.84 to 350.59)	427.98<br>(95.22 to 1270.55)	120.98<br>(27.14 to 356.94)	-0.11<br>(-0.79 to 0.58)
Paraguay	500.37<br>(120.59 to 1385.99)	146.5<br>(35.42 to 405.25)	1322.37<br>(317.7 to 3454.44)	150.93<br>(36.23 to 394.5)	0.01<br>(-0.42 to 0.44)
Peru	1532.96<br>(347.88 to 4308.84)	84.65<br>(19.24 to 237.46)	4762.03<br>(1112.31 to 12756.28)	81.77<br>(19.09 to 219.5)	-0.16<br>(-0.4 to 0.08)
Philippines	3332.22<br>(771.95 to 9531.14)	116.6<br>(26.94 to 327.88)	10936.17<br>(2550.59 to 30479.62)	126.49<br>(29.61 to 348.17)	0.3<br>(0.14 to 0.47)
Poland	7660.72<br>(1795.42 to 21475.81)	126.54<br>(29.67 to 351.61)	17121.55<br>(4144.09 to 46338.25)	124.15<br>(30 to 337.24)	-0.04<br>(-0.17 to 0.08)
Portugal	2899.13<br>(681.98 to 8099.76)	151.21<br>(35.45 to 418.67)	8544.71<br>(2137.31 to 22122.25)	146.19<br>(36.43 to 380.5)	-0.02<br>(-0.25 to 0.21)
Puerto Rico	564.91<br>(131.87 to 1601.29)	98.9<br>(22.96 to 278.87)	1749.12<br>(429.54 to 4586.54)	93.98<br>(22.84 to 250.67)	-0.13<br>(-0.58 to 0.32)
Qatar	9.36<br>(2.09 to 26.87)	175.67<br>(40.1 to 495.09)	61<br>(13.76 to 175.94)	156.51<br>(36.75 to 439.58)	-0.18<br>(-2.91 to 2.63)
Republic of Korea	5219.33<br>(1278.31 to 14199.73)	203.45<br>(50.05 to 543.2)	25773.58<br>(6676.2 to 63457.79)	171.09<br>(44.4 to 420.02)	-0.34<br>(-0.47 to -0.22)
Republic of Moldova	551.46<br>(128.28 to 1571.83)	121.25<br>(28.1 to 342.17)	1192.95<br>(283.93 to 3194.35)	112<br>(26.57 to 301.8)	-0.14<br>(-0.58 to 0.3)

Romania	3768.89 (873.86 to 10923.82)	119.19 (27.49 to 342.73)	8508.83 (1986.3 to 23830.53)	118.13 (27.49 to 330.61)	0.01 (-0.17 to 0.19)
Russian Federation	28282.03 (6571.91 to 80091.09)	127.24 (29.58 to 357.07)	50769.6 (11998.09 to 139682.1)	124.02 (29.34 to 340.99)	-0.03 (-0.09 to 0.04)
Rwanda	296.57 (67.45 to 859.16)	147.6 (34.16 to 419.41)	879.77 (202.17 to 2512.99)	173.21 (40.68 to 485.32)	0.52 (0.02 to 1.03)
Saint Kitts and Nevis	4.43 (1.01 to 12.86)	90.18 (20.41 to 257.27)	6.55 (1.48 to 18.64)	91.37 (20.89 to 257.28)	1.17 (-4.87 to 7.59)
Saint Lucia	10.52 (2.41 to 30.34)	98.89 (22.37 to 285.3)	36.02 (8.37 to 100.36)	96.56 (22.43 to 268.96)	-0.24 (-3.83 to 3.48)
Saint Vincent and the Grenadines	9.82 (2.25 to 28.12)	102.51 (23.39 to 290.75)	18.1 (4.1 to 51.16)	93.33 (21.02 to 263.77)	0.49 (-3.38 to 4.51)
Samoa	13.98 (3.2 to 39.33)	162.77 (37.61 to 451.3)	24.84 (5.72 to 71.07)	155.96 (36.22 to 440.67)	-0.8 (-3.7 to 2.19)
San Marino	10.13 (2.48 to 26.2)	139.44 (34.13 to 360.94)	26.79 (7.04 to 66.16)	126.75 (32.66 to 319.28)	-0.48 (-5.25 to 4.53)
Sao Tome and Principe	8.15 (1.84 to 23.61)	105.02 (23.84 to 302.01)	11.26 (2.6 to 32.26)	104.09 (24.15 to 295.21)	-0.73 (-4.75 to 3.47)
Saudi Arabia	957.57 (223.85 to 2661.14)	169.43 (39.95 to 463.27)	1657.93 (383.08 to 4756.45)	152.34 (35.64 to 427.63)	-0.41 (-0.71 to -0.11)

Senegal	362.13 (81.78 to 1023.78)	112.54 (25.33 to 314.14)	962.47 (208.28 to 2786.57)	122.79 (26.64 to 351.58)	0.38 (-0.08 to 0.83)
Serbia	1369.39 (301.57 to 4018.12)	120.62 (26.57 to 352.88)	3466.17 (796.83 to 9773.92)	117.29 (27.01 to 330.48)	-0.1 (-0.37 to 0.17)
Seychelles	12.04 (2.81 to 33.47)	129.82 (30.32 to 359.69)	17.81 (4.06 to 49.64)	122.24 (27.97 to 338.74)	0.12 (-3.39 to 3.76)
Sierra Leone	216.84 (48.46 to 624.96)	99.01 (21.91 to 282.64)	356.56 (80.22 to 1048.71)	100.59 (22.77 to 292.33)	0.18 (-0.5 to 0.87)
Singapore	277.01 (66.45 to 761.06)	109.67 (26.45 to 297.22)	1453.92 (371.89 to 3713.88)	105.48 (27.03 to 269.51)	-0.1 (-0.62 to 0.43)
Slovakia	1069.29 (245.9 to 2987.25)	122.26 (28.21 to 339.74)	1922.57 (455.11 to 5209.76)	121.78 (28.83 to 329.27)	-0.02 (-0.36 to 0.33)
Slovenia	485.02 (113.52 to 1337.35)	120.48 (28.28 to 331.05)	1218.17 (298.31 to 3228.28)	122.62 (29.95 to 326.81)	-0.04 (-0.56 to 0.49)
Solomon Islands	12.38 (2.72 to 37.04)	138.76 (30.75 to 408.77)	36.28 (8.02 to 105.36)	139 (31.16 to 400.68)	0.18 (-2.42 to 2.86)
Somalia	199.17 (44.32 to 575.39)	134.13 (29.84 to 382.46)	477.4 (104.94 to 1396.01)	144.85 (32.48 to 423.89)	0.47 (-0.13 to 1.07)
South Africa	3374.13 (775.92 to 9396.07)	126.92 (29.16 to 351.9)	6613.16 (1545.66 to 18716.04)	132.99 (31.18 to 371.64)	0.11 (-0.05 to 0.28)

South Sudan	325.13<br>(71.52 to 924.65)	135.73<br>(29.76 to 382.33)	434.39<br>(92.87 to 1223.1)	138.78<br>(30.05 to 386.38)	0.18<br>(-0.37 to 0.73)
Spain	14367.82<br>(3587.17 to 38480.93)	156.02<br>(38.84 to 415.87)	36099.6<br>(9399.23 to 90550.63)	146.6<br>(38.06 to 370.76)	-0.19<br>(-0.3 to -0.08)
Sri Lanka	1236.36<br>(287.34 to 3525.93)	111.66<br>(25.93 to 315.19)	3803.16<br>(848.76 to 10426.48)	111.35<br>(25.13 to 302.89)	0<br>(-0.24 to 0.24)
Sudan	1378.25<br>(319.32 to 3964.56)	147.8<br>(34.16 to 416.93)	2740.04<br>(613.86 to 7572.87)	137.6<br>(31.13 to 376.65)	-0.27<br>(-0.52 to -0.01)
Suriname	37.95<br>(8.88 to 105.73)	99.13<br>(23.27 to 276.58)	89.21<br>(20.13 to 242.41)	95.98<br>(21.74 to 260.69)	-0.19<br>(-1.79 to 1.44)
Sweden	4439.97<br>(1082.87 to 11802.11)	145.17<br>(35.34 to 385.99)	6915.03<br>(1743.71 to 17521.75)	136.83<br>(34.46 to 348.95)	-0.03<br>(-0.28 to 0.22)
Switzerland	3066.24<br>(756.88 to 8139.65)	145.93<br>(35.89 to 387.91)	6350.97<br>(1652.97 to 15723.91)	145.26<br>(37.77 to 362.43)	-0.19<br>(-0.45 to 0.07)
Syrian Arab Republic	850.44<br>(198.45 to 2424.51)	149.46<br>(35.03 to 423.6)	1678.84<br>(378.45 to 4868.83)	140.44<br>(31.71 to 404.78)	-0.18<br>(-0.51 to 0.14)
Taiwan (Province of China)	1839.7<br>(435.48 to 5140.78)	117.36<br>(27.89 to 321.02)	10020.71<br>(2500.41 to 25811.22)	124.5<br>(31.05 to 322.9)	-0.12<br>(-0.32 to 0.08)
Tajikistan	457.12<br>(103.72 to 1244.47)	125.08<br>(28.43 to 340.08)	664.83<br>(155.17 to 1868.94)	127.16<br>(29.94 to 352.23)	0.07<br>(-0.43 to 0.58)

Thailand	5390.39 (1248.28 to 14842.91)	134 (31.21 to 364.68)	23369.49 (5634.08 to 60413.49)	124.21 (29.96 to 321.24)	-0.27 (-0.38 to -0.16)
Timor-Leste	25.53 (5.64 to 74.04)	111.17 (24.74 to 319.99)	104.56 (23.72 to 306.06)	123.2 (28.06 to 356.37)	0.44 (-1.06 to 1.96)
Togo	115.81 (26.02 to 332.06)	109.59 (24.58 to 310.21)	319.43 (69.13 to 929.02)	118.32 (25.94 to 338.41)	0.32 (-0.45 to 1.09)
Tokelau	0.28 (0.07 to 0.83)	166.18 (39.03 to 481.56)	0.36 (0.08 to 0.99)	149.46 (34.77 to 409.58)	NA
Tonga	9.45 (2.25 to 26.39)	153.37 (36.94 to 421.07)	18.14 (4.26 to 48.96)	152.46 (35.89 to 410.34)	-0.24 (-4.16 to 3.83)
Trinidad and Tobago	100.49 (23.06 to 288.94)	96.31 (21.96 to 274.55)	277 (62.99 to 771.32)	92.66 (21.17 to 257.21)	-0.01 (-0.96 to 0.96)
Tunisia	846.96 (195.64 to 2384.91)	166.95 (38.85 to 460.37)	2689.57 (623.81 to 7395.52)	153.37 (35.78 to 418.59)	-0.3 (-0.61 to 0)
Turkey	7087.03 (1677.65 to 19248.54)	172.11 (41.16 to 461.49)	19915.63 (4750.8 to 55413.36)	154.24 (36.86 to 426.62)	-0.47 (-0.58 to -0.36)
Turkmenistan	259.31 (60.37 to 732.43)	123.22 (28.82 to 344.02)	563.51 (130.29 to 1568.36)	116.11 (26.95 to 323.08)	-0.29 (-0.89 to 0.32)
Tuvalu	0.79 (0.18 to 2.31)	159.32 (36.7 to 465.27)	1.56 (0.35 to 4.55)	152.13 (34.55 to 437.91)	NA

Uganda	756.48<br>(176.18 to 2092.66)	131.42<br>(30.61 to 358.86)	2041.34<br>(467.95 to 5771.85)	158.37<br>(37.06 to 438.11)	0.68<br>(0.35 to 1)
Ukraine	11820.89<br>(2731.61 to 33749.41)	127.21<br>(29.32 to 360.02)	16326.67<br>(3742.31 to 45797.97)	123.78<br>(28.38 to 346.57)	0.04<br>(-0.08 to 0.15)
United Arab Emirates	46.77<br>(10.99 to 127.74)	164.41<br>(39.74 to 432.4)	144.9<br>(31.71 to 416.74)	142.48<br>(33.25 to 391.9)	-0.49<br>(-1.73 to 0.75)
United Kingdom	22773.56<br>(5468.17 to 62029.75)	140<br>(33.56 to 380.12)	37497.13<br>(9376.07 to 97777.97)	133.36<br>(33.26 to 349.62)	0.03<br>(-0.07 to 0.13)
United Republic of Tanzania	1413.84<br>(327 to 3992.82)	150.29<br>(35.17 to 411.16)	3807.62<br>(896.46 to 10729.01)	152.21<br>(36.32 to 421.84)	0.02<br>(-0.21 to 0.25)
United States of America	106049.02<br>(26450.97 to 277739.87)	175.17<br>(43.64 to 458.5)	197596.87<br>(50125.48 to 505904.7)	171.94<br>(43.55 to 442.4)	-0.1<br>(-0.14 to -0.07)
United States Virgin Islands	9.07<br>(2.11 to 26)	101.82<br>(23.71 to 287.97)	25.17<br>(5.76 to 68.63)	89.53<br>(20.42 to 245.54)	-0.32<br>(-3.82 to 3.32)
Uruguay	790.28<br>(187.77 to 2190.41)	122.13<br>(29.01 to 336.78)	1569.41<br>(384.09 to 4158.82)	124.74<br>(30.45 to 332.28)	0.11<br>(-0.36 to 0.58)
Uzbekistan	2020.15<br>(479.65 to 5584.39)	118.79<br>(28.2 to 328.42)	2974.06<br>(691.28 to 8425.68)	113.76<br>(26.69 to 318.44)	-0.09<br>(-0.33 to 0.16)
Vanuatu	5.86<br>(1.28 to 17)	136.09<br>(29.65 to 393.23)	17.17<br>(3.86 to 50.4)	135.87<br>(30.58 to 395.46)	0.65<br>(-3.44 to 4.91)

Venezuela (Bolivarian Republic of)	1641.54<br>(394.55 to 4515.57)	123.02<br>(29.65 to 335.55)	5669.36<br>(1349.31 to 15037.95)	122.03<br>(28.99 to 325.41)	-0.04<br>(-0.26 to 0.19)
Viet Nam	7750.7<br>(1772.08 to 21244.51)	147.27<br>(33.89 to 399.73)	19125.9<br>(4480.06 to 53654.26)	160.72<br>(37.98 to 446.02)	0.23<br>(0.12 to 0.33)
Yemen	649.07<br>(146.73 to 1858.51)	160.66<br>(36.87 to 449.11)	1913.02<br>(417.41 to 5670.22)	153.76<br>(33.76 to 452.21)	-0.18<br>(-0.5 to 0.15)
Zambia	307.46<br>(69.56 to 873.27)	129.9<br>(29.36 to 365.65)	760.36<br>(167.01 to 2161.33)	144.75<br>(32.6 to 401.89)	0.42<br>(-0.08 to 0.92)
Zimbabwe	528.75<br>(122.45 to 1453.44)	145.49<br>(34.13 to 398.55)	659.53<br>(146.25 to 1908.71)	136.05<br>(30.57 to 384.53)	-0.06<br>(-0.48 to 0.35)

ASMR, age-standardized mortality rate; 95% UI, 95% Uncertainty Intervals; 95% CI, 95% Confidence Interval

S7 Table Trends in DALYs cases and Age standardized DALY rate of Alzheimer's disease and other dementias from 1992 to 2021. (S7 Table expands on Table 4 by adding data from 21 regions.)

Characteristics	1992		2021		1992 to 2021
	DALYs, n (95% UI)	ASR-DALYs per 100 000, n (95% UI)	DALYs, n (95% UI)	ASR-DALYs per 100 000, n (95% UI)	Net Drift (%/year)
Global	14101639.87 (6459705.64 to 31769303.63)	2595.87 (1173.17 to 5832.09)	35625499.28 (16274212.21 to 78478461.82)	2621.1 (1192.88 to 5766.16)	0 (-0.01 to 0)
Sex					
Male	4592937.56 (2075089.91 to 10660013.61)	2107.06 (936.04 to 4894.38)	12202362.18 (5523213.91 to 28099714.48)	2161.17 (967.82 to 4977.54)	0.05 (0.04 to 0.07)
Female	9508702.31 (4365032.26 to 21096167.49)	2886.72 (1312.06 to 6394.16)	23423137.1 (10758455.48 to 50304931.19)	2935.7 (1349.85 to 6306.48)	0.01 (0 to 0.02)
SDI					
High SDI	5384336.44 (2488746.12 to 11766194.75)	2760.32 (1266.98 to 6032.22)	11616224.56 (5340754.1 to 24601306.68)	2678.83 (1243.05 to 5685.58)	-0.05 (-0.07 to -0.04)
High-middle SDI	3688764.12 (1682649.8 to 8378239.47)	2685.15 (1202.32 to 6094.16)	9085513.25 (4192100.49 to 19979625.3)	2800.58 (1286.23 to 6161.8)	0.07 (0.05 to 0.08)
Middle SDI	3112813.37 (1414609.82 to 7111172.97)	2543.57 (1136.51 to 5804.25)	9884977.88 (4551968.54 to 21972268.5)	2645.21 (1207.99 to 5862.83)	0 (-0.02 to 0.02)

Low-middle SDI	1398381.06 (636239.72 to 3210488.09)	1949.64 (877.73 to 4465.75)	3800505.27 (1677834.08 to 8797773.54)	2089.61 (912.95 to 4816.92)	0.22 (0.21 to 0.24)
Low SDI	502223.29 (223512.04 to 1174710.82)	2075.71 (903.79 to 4869.4)	1208919.44 (519188.02 to 2880007.91)	2222.14 (936.36 to 5274.99)	0.22 (0.18 to 0.27)
Region					
Andean Latin America	49070.15 (22447.27 to 110321.53)	1609.42 (733.35 to 3618.88)	146877.87 (67997.4 to 320273.18)	1571.64 (727.62 to 3422.91)	-0.11 (-0.15 to -0.08)
Australasia	102381.45 (48044.13 to 222706.36)	2558.8 (1187.14 to 5576.9)	249388.84 (112527.74 to 535090.97)	2353.7 (1069.28 to 5054.96)	-0.26 (-0.28 to -0.23)
Caribbean	75423.34 (36250.22 to 164477.75)	1859.9 (882.68 to 4080.01)	170681.63 (81007.33 to 370500.27)	1817.14 (865.92 to 3940.4)	-0.13 (-0.16 to -0.1)
Central Asia	150531.7 (70013.13 to 335220.94)	2264.79 (1046.32 to 5047.93)	225177.7 (104560.79 to 504349.19)	2200.77 (1019.22 to 4908.53)	-0.1 (-0.13 to -0.08)
Central Europe	493597.9 (231983.77 to 1104364.6)	2273.96 (1053.39 to 5101.5)	921527.72 (427860.39 to 2017539.41)	2240.33 (1038.36 to 4910.24)	-0.04 (-0.06 to -0.03)
Central Latin America	231496.84 (111326.99 to 505559.01)	1978.53 (945.74 to 4320.9)	764313.2 (366392.23 to 1641202.05)	1943.63 (932.8 to 4169.05)	-0.06 (-0.08 to -0.04)
Central Sub-Saharan Africa	65012.75 (28370.25 to 151120.94)	3128.64 (1333.44 to 7238.01)	170426.14 (71167.45 to 407456.26)	3443.11 (1423.61 to 8141.58)	0.36 (0.33 to 0.4)
East Asia	2932136.45 (1307870.31 to 6769135.05)	3091.57 (1341.23 to 7148.6)	10141787.57 (4719921.86 to 22293156.49)	3230.29 (1485.14 to 7096.23)	-0.02 (-0.04 to -0.01)

Eastern Europe	948058.71 (446961.72 to 2126535.89)	2345.76 (1089.72 to 5278.84)	1418607.18 (658267.72 to 3145082.24)	2301.92 (1068.24 to 5097.54)	-0.05 (-0.06 to -0.03)
Eastern Sub-Saharan Africa	193617.34 (86060.59 to 447965.13)	2497.5 (1084.16 to 5785.88)	467896.07 (198432.47 to 1093448.02)	2676.26 (1115.92 to 6231.84)	0.24 (0.22 to 0.26)
High-income Asia Pacifc	858259.3 (387545.78 to 1888169.61)	2726.27 (1216.95 to 5977.72)	3006938.83 (1388524.54 to 6129199.24)	2683.52 (1257.15 to 5511.44)	0.08 (0.03 to 0.12)
High-income North America	2009137.81 (930018.83 to 4374116.75)	3036.03 (1401.53 to 6615.37)	3619805.69 (1650183.78 to 7809416.35)	2894.84 (1328 to 6249.9)	-0.17 (-0.19 to -0.15)
North Africa and Middle East	591756.77 (272739.16 to 1330294.22)	2979.15 (1354.81 to 6672.98)	1517932.38 (703886.04 to 3354155.45)	2771.37 (1273.93 to 6113.84)	-0.28 (-0.31 to -0.25)
Oceania	6771.27 (3104.64 to 15307.44)	2474.56 (1115.93 to 5599.82)	16702.73 (7627.07 to 37757.53)	2304.85 (1046.27 to 5177.78)	-0.29 (-0.4 to -0.17)
South Asia	1039312.24 (468956.91 to 2424637.9)	1585.81 (708.75 to 3693.32)	3339100.26 (1443205.17 to 7889173.85)	1786.26 (763.16 to 4204.34)	0.39 (0.36 to 0.42)
Southeast Asia	730888.84 (338793.36 to 1635724.16)	2347.51 (1073.33 to 5241.49)	2014034.41 (913325.69 to 4569248.45)	2424.37 (1089.03 to 5465.59)	0.07 (0.05 to 0.09)
Southern Latin America	157224.14 (74163.16 to 348123.47)	2197.86 (1023 to 4879.55)	334775.65 (155211.45 to 726602.48)	2136.86 (990.99 to 4640.56)	-0.08 (-0.1 to -0.05)
Southern Sub-Saharan Africa	85499.28 (39021.42 to 191328.72)	2347.41 (1058.17 to 5271.18)	162746.56 (72897.26 to 376833.37)	2371.49 (1044.46 to 5479.85)	0.02 (-0.01 to 0.05)

Tropical Latin America	362075.27 (163316.19 to 820252.53)	2960.51 (1321.57 to 6657.66)	1201314.69 (545027.63 to 2634375)	2921.78 (1325.83 to 6398.39)	-0.05 (-0.07 to -0.03)
Western Europe	2836233.89 (1306423.19 to 6207360.97)	2672.61 (1221.35 to 5854.62)	5346876.17 (2449226.94 to 11316885.14)	2584.51 (1194.92 to 5482.16)	-0.11 (-0.13 to -0.08)
Western Sub-Saharan Africa	183154.42 (79459.04 to 429606.54)	1803.72 (761.05 to 4253.06)	388587.98 (163133.93 to 936482.8)	1858.43 (762.61 to 4479.99)	0.17 (0.15 to 0.19)

SDI, socio-demographic index; DALYs, disability-adjusted life-years; ASR, age-standardized rate; 95% UI, 95% Uncertainty Interval; 95% CI, 95% Confidence Interval.

S8 Table Trends in DALYs cases and Age standardized DALY rate of Alzheimer's disease and other dementias from 1992 to 2021 across countries and territories.

Location	DALYs 1992, (95% UI)	ASR-DALYs 1992, (95% UI)	DALYs 2021, (95% UI)	ASR-DALYs 2021, (95% UI)	Net Drift (%/year)
Afghanistan	27165.69 (11326.85 to 65914.52)	3502.09 (1445.49 to 8421.59)	31790.17 (13575.91 to 74082.73)	3361.84 (1417.66 to 7823.68)	-0.15 (-0.21 to -0.09)
Albania	6410.71 (2956.33 to 14252.14)	2319.69 (1064.47 to 5144.55)	15933.15 (7418.57 to 35585.67)	2273.28 (1046.53 to 5085.9)	-0.06 (-0.16 to 0.04)
Algeria	41501.77 (19384.72 to 95688.47)	2956.1 (1324.02 to 6873.04)	120968.52 (56218.84 to 267776.22)	2785.38 (1262.11 to 6228.13)	-0.18 (-0.26 to -0.11)
American Samoa	59.21 (26.46 to 137.45)	2514.68 (1101.14 to 5783.22)	135.26 (61.5 to 303.31)	2351.28 (1054.68 to 5265.9)	-0.08 (-1.22 to 1.07)
Andorra	237.14 (110.64 to 503.04)	2612.36 (1199.79 to 5566.87)	731.12 (331.8 to 1594.3)	2438.86 (1119.17 to 5327.75)	-0.21 (-0.79 to 0.37)
Angola	10194.99 (4556.98 to 23923.75)	2994.24 (1308.73 to 6987.69)	34100.34 (14405.92 to 81626.25)	3320.26 (1385.22 to 7909.64)	0.37 (0.28 to 0.46)
Antigua and Barbuda	188.31 (88.86 to 420.99)	1867.41 (877.81 to 4186.39)	267.42 (127.32 to 599.24)	1807.25 (850.73 to 4065)	-0.07 (-0.76 to 0.62)
Argentina	110506.14 (51824.54 to 244918.08)	2217.17 (1025.46 to 4926.47)	214388.03 (99079.93 to 466462.35)	2144.77 (990.49 to 4669.21)	-0.09 (-0.11 to -0.06)

Armenia	8207.63 (3868.4 to 18419.82)	2240.94 (1051.09 to 5016)	17060.71 (7812.09 to 37367.99)	2314.88 (1059.76 to 5081.47)	0.19 (0.09 to 0.29)
Australia	84170.11 (39589.09 to 182682.19)	2528.85 (1175.97 to 5501.02)	208505.98 (93979.11 to 447287.91)	2316.47 (1051.39 to 4975.05)	-0.26 (-0.29 to -0.23)
Austria	56878.53 (26263.43 to 126769.31)	2610.54 (1194.98 to 5835.42)	97320.34 (44830.1 to 209381.3)	2483.6 (1154.48 to 5351.17)	-0.15 (-0.19 to -0.11)
Azerbaijan	15758.35 (7357.33 to 34638.25)	2308.45 (1066.99 to 5088.26)	26769.33 (12329.78 to 60684.19)	2231.61 (1027.64 to 5034.04)	-0.12 (-0.19 to -0.05)
Bahamas	419.16 (200.68 to 923.67)	1866.01 (885.97 to 4116.71)	1004.32 (475.48 to 2198.17)	1811.59 (856.42 to 3957.02)	-0.09 (-0.48 to 0.31)
Bahrain	416.88 (192.59 to 951.42)	3023.46 (1344.99 to 6961.04)	1806.42 (843.51 to 4102.36)	2707.58 (1223.77 to 6257.04)	-0.47 (-1.04 to 0.1)
Bangladesh	101609.85 (45152.28 to 234457.93)	1697.4 (747.28 to 3916.06)	332669.89 (143717.31 to 794124.11)	1802.29 (769.22 to 4325.91)	0.14 (0.11 to 0.16)
Barbados	1025.77 (499.82 to 2258.48)	1839.64 (881.41 to 4081.45)	1615.83 (762.36 to 3533.13)	1819.87 (855.42 to 3983.97)	-0.02 (-0.32 to 0.29)
Belarus	47601.87 (22378.5 to 106014.1)	2302.68 (1077.21 to 5133.65)	64652.28 (30112.33 to 142870.15)	2287.86 (1066.18 to 5050.8)	-0.01 (-0.06 to 0.03)
Belgium	83362.31 (38603.27 to 180460.14)	2974.38 (1365.99 to 6454.38)	137877.28 (62899.95 to 290655.05)	2666.22 (1229.34 to 5641.89)	-0.31 (-0.34 to -0.27)

Belize	303.59<br>(144.93 to 651.83)	1880.25<br>(899.63 to 4035.22)	767.19<br>(363.55 to 1671.81)	1861.28<br>(884.99 to 4036.68)	-0.02<br>(-0.49 to 0.45)
Benin	4950.5<br>(2159.46 to 11467.66)	1856.97<br>(794.78 to 4330.78)	10671.87<br>(4545.8 to 25256.31)	1830.25<br>(765.43 to 4333.59)	-0.08<br>(-0.2 to 0.04)
Bermuda	182.12<br>(87.11 to 397.82)	1922.19<br>(907.61 to 4218.01)	502.2<br>(239.11 to 1059.38)	1873.28<br>(895.49 to 3950.94)	-0.16<br>(-0.77 to 0.46)
Bhutan	447.9<br>(199.18 to 1012.26)	1732.71<br>(762.99 to 3908.76)	1704.08<br>(696.34 to 4095.53)	1956.53<br>(794.43 to 4699)	0.44<br>(0.07 to 0.82)
Bolivia (Plurinational State of)	6815.84<br>(3092.29 to 15621.4)	1697.78<br>(756.67 to 3900.27)	19807.39<br>(9025.1 to 44511.49)	1701.18<br>(765.72 to 3811.33)	0.01<br>(-0.09 to 0.1)
Bosnia and Herzegovina	11972.83<br>(5550.28 to 26642.55)	2242.92<br>(1037.24 to 4981.68)	23441.49<br>(11166.65 to 50456.99)	2172.79<br>(1030.9 to 4669.26)	-0.12<br>(-0.2 to -0.04)
Botswana	1433.06<br>(627.08 to 3389.8)	2460.61<br>(1054.08 to 5893.11)	3640.65<br>(1612.24 to 8444.69)	2360.64<br>(1037.24 to 5422.42)	-0.08<br>(-0.32 to 0.16)
Brazil	352515.57<br>(158996.67 to 799043.97)	2969.11<br>(1325.01 to 6677.2)	1177308.12<br>(534317.05 to 2583656.08)	2926.54<br>(1328.4 to 6413.75)	-0.05<br>(-0.08 to -0.03)
Brunei Darussalam	257.5<br>(117.12 to 588.56)	2242.51<br>(984.22 to 5179.98)	744.36<br>(340.69 to 1632.86)	2305.92<br>(1018.93 to 5105.55)	0.24<br>(-0.37 to 0.87)
Bulgaria	36427.71<br>(17513.05 to 79937.59)	2234.38<br>(1040.83 to 5001.54)	55753.47<br>(26726.45 to 122464.3)	2212.12<br>(1040.31 to 4919.69)	-0.07<br>(-0.15 to 0.01)

Burkina Faso	10300.4<br>(4358.95 to 24158.62)	2151.06<br>(878.63 to 5082.23)	20181.48<br>(8545.98 to 47858.99)	1983.92<br>(818.48 to 4709.21)	-0.33<br>(-0.41 to -0.24)
Burundi	6178.07<br>(2825.88 to 14053.94)	2400.4<br>(1070.35 to 5444.53)	11814.36<br>(5000.44 to 28405.54)	2509.35<br>(1043.36 to 6020.04)	0.18<br>(0.06 to 0.31)
Cabo Verde	764.85<br>(344.81 to 1718.78)	1765.36<br>(792.61 to 3974.28)	1238.71<br>(538.03 to 2839.95)	1790.88<br>(783.98 to 4087.57)	-0.01<br>(-0.34 to 0.32)
Cambodia	11870.79<br>(5438.88 to 26610.84)	2407.73<br>(1089.42 to 5429.9)	37055.68<br>(16258.71 to 86804.98)	2686.57<br>(1157.68 to 6285.79)	0.43<br>(0.35 to 0.51)
Cameroon	9865.18<br>(4130.05 to 23217.6)	2006.53<br>(822.23 to 4741.64)	23907.08<br>(10032.87 to 58842.71)	1887.94<br>(775.83 to 4669.47)	-0.22<br>(-0.3 to -0.13)
Canada	152703.84<br>(79278.55 to 310786.73)	2667.57<br>(1375.89 to 5449.2)	340830.56<br>(168115.05 to 697615.36)	2407.33<br>(1197.73 to 4921.37)	-0.4<br>(-0.42 to -0.37)
Central African Republic	3158.6<br>(1373.97 to 7198.17)	3361.69<br>(1446.39 to 7580.95)	5401.97<br>(2323.56 to 12363.98)	3304.03<br>(1385.06 to 7491.26)	-0.09<br>(-0.28 to 0.1)
Chad	6512.74<br>(2912.94 to 15033.29)	1801.91<br>(787.88 to 4195.94)	10854.97<br>(4604.99 to 25545.46)	1802.31<br>(752.2 to 4236.11)	0.02<br>(-0.09 to 0.13)
Chile	31696.45<br>(15120.33 to 69466.07)	2112.44<br>(996.61 to 4638.15)	94525.88<br>(44317.26 to 202475.2)	2099.84<br>(984.77 to 4500.8)	-0.02<br>(-0.06 to 0.03)
China	2849626.79<br>(1269995.06 to 6581634.61)	3135.23<br>(1357.54 to 7253.88)	9860689.08<br>(4592548.94 to 21646645.25)	3272.96<br>(1505.09 to 7181.81)	-0.03<br>(-0.04 to -0.01)

Colombia	52116.48 (24951.38 to 113620.74)	2083.03 (993.47 to 4538.14)	205361.14 (99748.22 to 433077.77)	2080.54 (1015.45 to 4402.4)	0 (-0.03 to 0.04)
Comoros	525.56 (233.3 to 1205.13)	2549.31 (1104.91 to 5803.31)	1580.58 (654.59 to 3802.05)	2747.67 (1122.89 to 6576.9)	0.28 (-0.09 to 0.65)
Congo	3378.13 (1466.63 to 7771.93)	3389.16 (1408.78 to 7845.41)	7903.94 (3287.25 to 18549.15)	3328.05 (1356.98 to 7683.15)	-0.05 (-0.25 to 0.15)
Cook Islands	39.46 (17.54 to 90.86)	2561.49 (1118.73 to 5894.48)	96.94 (44.28 to 213.44)	2304.05 (1043.38 to 5085.33)	-0.44 (-1.78 to 0.91)
Costa Rica	6105.93 (2928.8 to 13270.69)	2109.23 (1010.08 to 4581.87)	19951.8 (9555.05 to 41985.62)	2069 (997.94 to 4356.42)	-0.05 (-0.15 to 0.05)
Coted'Ivoire	6956.07 (3006.18 to 16119.61)	1894.05 (792.31 to 4380.24)	20089.01 (8528.52 to 48187.9)	1876.14 (780.14 to 4500.52)	-0.01 (-0.11 to 0.09)
Croatia	19810.52 (9249.53 to 44323.8)	2298.02 (1061.38 to 5167.16)	38077.12 (17801.94 to 83692.19)	2221.97 (1032.19 to 4898.15)	-0.1 (-0.17 to -0.04)
Cuba	30369.49 (14537.66 to 67240.18)	1785.23 (842.23 to 3978.21)	64215.67 (30155.36 to 142985.75)	1754.96 (829.78 to 3898.53)	-0.15 (-0.2 to -0.1)
Cyprus	2884.59 (1297.52 to 6697.58)	2869.04 (1222.23 to 6829.63)	8361.74 (3850.51 to 18588.74)	2573.63 (1144.1 to 5823.94)	-0.42 (-0.71 to -0.14)
Czechia	49106.97 (23029.41 to 110211.22)	2227.67 (1031.14 to 5018.86)	89763.99 (41818.19 to 194236.65)	2221.65 (1032.33 to 4815.96)	0 (-0.05 to 0.04)

Democratic People's Republic of Korea	43875.57 (19606.88 to 101147.74)	2455.96 (1081.02 to 5649.4)	113751.47 (50095.88 to 259972.83)	2504.71 (1090.8 to 5711.02)	0.12 (0.08 to 0.16)
Democratic Republic of the Congo	45269.16 (19668.34 to 104969.19)	3105.27 (1320.5 to 7211.06)	117701.65 (48586.15 to 279640.25)	3494.96 (1435.84 to 8199.47)	0.45 (0.41 to 0.5)
Denmark	36595.38 (16264.46 to 83236.55)	2353.62 (1039.87 to 5367.39)	53489.21 (22449.69 to 121000.52)	2245.65 (945.32 to 5083.41)	-0.25 (-0.3 to -0.19)
Djibouti	316.55 (137.53 to 761.32)	2665.87 (1137 to 6310.92)	1488.28 (625.51 to 3448.34)	2801.14 (1152.55 to 6501.55)	0.18 (-0.28 to 0.63)
Dominica	183.14 (86.83 to 400.52)	1908.15 (889.74 to 4212.9)	233.68 (110.55 to 518.11)	1861.51 (876.12 to 4127.56)	-0.11 (-0.82 to 0.6)
Dominican Republic	10088.61 (4833.73 to 21467.99)	1902.64 (897.46 to 4077.89)	29990.51 (14234.6 to 64448.83)	1883.79 (893.64 to 4047.47)	-0.07 (-0.15 to 0.01)
Ecuador	12656.6 (5835.61 to 28624.91)	1616.95 (738.8 to 3670.59)	39409.11 (18300.28 to 87019.71)	1565.85 (720.43 to 3475.78)	-0.08 (-0.15 to -0.01)
Egypt	73479.66 (33801.2 to 169014.13)	2823.81 (1264.97 to 6541.64)	153359.99 (70909.85 to 347434.47)	2629.45 (1196.02 to 5915.17)	-0.3 (-0.33 to -0.26)
El Salvador	10661.04 (5039.86 to 23375.83)	2114.28 (1002.49 to 4635.73)	25098.73 (11921.54 to 52552.21)	2098.62 (1006.01 to 4407.04)	-0.04 (-0.12 to 0.04)
Equatorial Guinea	574.02 (250.57 to 1343.98)	3091.57 (1324.51 to 7192.85)	1616.93 (681.46 to 3795.5)	3271.91 (1355.11 to 7644.79)	0.18 (-0.2 to 0.56)

Eritrea	1929.93<br>(857.74 to 4436.47)	2561.5<br>(1118.83 to 5865.21)	6622.81<br>(2736.99 to 15886.05)	2904.87<br>(1167.94 to 6894.21)	0.4<br>(0.17 to 0.64)
Estonia	7382.56<br>(3476.7 to 16405.78)	2286.92<br>(1062.72 to 5111.29)	13472.65<br>(6173.18 to 29392.33)	2343.98<br>(1076.02 to 5117.88)	0.08<br>(-0.04 to 0.19)
Eswatini	735.05<br>(322.39 to 1688.92)	2439.3<br>(1047.97 to 5635.3)	1272.14<br>(542.93 to 2968.67)	2442.88<br>(1032.83 to 5638.59)	0.11<br>(-0.24 to 0.46)
Ethiopia	49054.88<br>(21081.69 to 117648.82)	2705.56<br>(1136.27 to 6424.99)	141198.38<br>(59070.68 to 330829.11)	2799.17<br>(1155.34 to 6519.47)	0.12<br>(0.07 to 0.17)
Fiji	835.96<br>(372.32 to 1950.57)	2480.77<br>(1084.68 to 5807.83)	1784.87<br>(824.2 to 3914.36)	2350.51<br>(1060.52 to 5219.72)	-0.22<br>(-0.54 to 0.1)
Finland	35329.94<br>(15947.88 to 77971.75)	2777.56<br>(1243.27 to 6135.87)	70870.39<br>(31312.84 to 152024.69)	2555.39<br>(1139.02 to 5495.53)	-0.23<br>(-0.29 to -0.18)
France	363697.85<br>(163330.75 to 810411.65)	2254.41<br>(1005.94 to 5043.93)	694675.71<br>(308797.2 to 1496941.1)	2137.67<br>(966.3 to 4602.57)	-0.17<br>(-0.19 to -0.15)
Gabon	2437.85<br>(1033.6 to 5554.81)	3452.83<br>(1433.53 to 7791)	3701.31<br>(1569.77 to 8621.32)	3424.47<br>(1428.45 to 7906.68)	-0.07<br>(-0.25 to 0.12)
Gambia	766.33<br>(332.84 to 1771.45)	1990.09<br>(841.16 to 4615.3)	2224.33<br>(905.15 to 5432.42)	1979.15<br>(791.58 to 4836.97)	-0.06<br>(-0.36 to 0.24)
Georgia	21561.34<br>(10150.86 to 47837.65)	2268.69<br>(1061.27 to 5042.8)	27408.73<br>(12665.11 to 60132.91)	2316.39<br>(1074.39 to 5109.54)	0.09<br>(0.02 to 0.15)

Germany	687021.2 (327970.78 to 1450910.39)	2965.13 (1410.13 to 6270.52)	1280635.8 (608757.89 to 2624594.55)	2968.29 (1417.23 to 6101.06)	0.03 (-0.04 to 0.1)
Ghana	11489.13 (5042.89 to 26964.32)	1729.08 (741.29 to 4072.96)	32516.24 (13569.59 to 78771.43)	1901.24 (775.91 to 4637.02)	0.35 (0.27 to 0.42)
Greece	65513.67 (30578.16 to 145220)	2573.22 (1182.77 to 5727.94)	142639.77 (65299.75 to 308151.68)	2504.41 (1157.98 to 5409.47)	-0.09 (-0.13 to -0.05)
Greenland	87.91 (42.11 to 196.14)	2823.62 (1313.81 to 6374.44)	209.64 (100.4 to 449.38)	2730.05 (1284.07 to 5840.14)	-0.1 (-1.06 to 0.88)
Grenada	270.51 (129.37 to 598.7)	1859.08 (892.22 to 4111.09)	275.22 (130.09 to 608.84)	1894.23 (887.02 to 4196.51)	0.08 (-0.54 to 0.7)
Guam	196.2 (89.48 to 444.75)	2418.64 (1083.88 to 5454.54)	843.6 (413.82 to 1676.06)	2131.73 (1047.32 to 4302.66)	-0.39 (-1 to 0.22)
Guatemala	8288.23 (3926.88 to 18623.81)	2140.58 (990.79 to 4857.62)	32503.88 (15622.62 to 69724.04)	2088.89 (990.88 to 4518.03)	-0.08 (-0.18 to 0.02)
Guinea	7951.74 (3517.87 to 18184.37)	1798.39 (780.98 to 4144.77)	12548.64 (5352.53 to 29483.19)	1860.61 (783.4 to 4367.17)	0.07 (-0.03 to 0.16)
Guinea-Bissau	733.77 (314.13 to 1730.79)	1908.96 (796.53 to 4526.72)	1216.51 (506.25 to 2964.37)	1961.1 (799.45 to 4807.03)	0.08 (-0.28 to 0.45)
Guyana	826.82 (398.47 to 1806.03)	1746.05 (835.26 to 3836.47)	1408.99 (668.95 to 3122.77)	1782.56 (838.87 to 3935.27)	0 (-0.3 to 0.31)

Haiti	6735.08<br>(3175.83 to 15020.82)	1958.9<br>(912.45 to 4406.25)	13891.68<br>(6431.71 to 31684.42)	1866.73<br>(852.24 to 4250.56)	-0.16<br>(-0.27 to -0.04)
Honduras	6494.21<br>(3013.43 to 14402.8)	2320.9<br>(1069.67 to 5144.04)	19432.55<br>(8658.53 to 44637.06)	2481.04<br>(1089.55 to 5689.5)	0.22<br>(0.12 to 0.32)
Hungary	50379.08<br>(23583.05 to 113559.39)	2236.58<br>(1024.63 to 5068.69)	83255.08<br>(38916.49 to 180295.77)	2211.53<br>(1032.99 to 4796.71)	0.01<br>(-0.04 to 0.05)
Iceland	1455.75<br>(691.42 to 3127.57)	2645.37<br>(1257.88 to 5689.34)	2958.67<br>(1390.15 to 6137.21)	2506.66<br>(1188.92 to 5214.13)	-0.2<br>(-0.45 to 0.06)
India	786835.83<br>(356188.67 to 1832172.88)	1528.28<br>(687.75 to 3543.32)	2698981.73<br>(1168012.7 to 6338627.55)	1771.16<br>(759.19 to 4141.08)	0.5<br>(0.46 to 0.54)
Indonesia	242647.66<br>(115343.07 to 537173.98)	2167.94<br>(1020.17 to 4807.96)	603591.17<br>(276306.6 to 1391580.28)	2443.17<br>(1086.55 to 5689.39)	0.3<br>(0.29 to 0.32)
Iran (Islamic Republic of)	81083.54<br>(37489.31 to 182906.25)	2943.3<br>(1344.52 to 6614.89)	292167.17<br>(135124.57 to 639836.16)	2752.78<br>(1268.98 to 6021.1)	-0.22<br>(-0.25 to -0.2)
Iraq	33552.68<br>(15573.5 to 73203.09)	2858.02<br>(1326.83 to 6221.95)	69979.91<br>(32128.74 to 155697.41)	2753.75<br>(1246.86 to 6133.06)	-0.25<br>(-0.3 to -0.21)
Ireland	17389.09<br>(8075.73 to 38932.83)	2591.19<br>(1180.14 to 5835.33)	35649.16<br>(16520.15 to 75984.55)	2434.26<br>(1128.08 to 5194.26)	-0.18<br>(-0.26 to -0.11)
Israel	20591.45<br>(9518 to 45932.04)	2521.86<br>(1142.66 to 5648.59)	58884.82<br>(26750.84 to 125402.72)	2457.16<br>(1122.8 to 5239.61)	-0.11<br>(-0.17 to -0.04)

Italy	475480.04 (210703.42 to 1060721.62)	3004.73 (1314.62 to 6709.69)	1001075.62 (461253.64 to 2095365.86)	2957.36 (1373.82 to 6225.28)	-0.12 (-0.19 to -0.05)
Jamaica	6616.68 (3280.77 to 14080.61)	1962.14 (971.12 to 4181.99)	11491.07 (5550.54 to 24460.72)	1904.43 (938.06 to 4054.76)	-0.17 (-0.29 to -0.06)
Japan	746726.1 (336994.62 to 1638863.09)	2677.55 (1196.4 to 5858.16)	2524923.21 (1164548.64 to 5131178.36)	2652.07 (1243.99 to 5450.22)	0.09 (0.05 to 0.14)
Jordan	4103.06 (1917.84 to 9085.86)	2794.35 (1297.59 to 6150.78)	20731.81 (9953.87 to 45710.95)	2618 (1240.52 to 5770.01)	-0.28 (-0.4 to -0.17)
Kazakhstan	38968.15 (18041.18 to 87094.47)	2301.3 (1057.95 to 5140.62)	46923.69 (21946.92 to 105258.59)	2156.14 (993.49 to 4859.19)	-0.27 (-0.32 to -0.22)
Kenya	24785.4 (10915.37 to 56314.44)	2480.53 (1073.72 to 5655.91)	64097.92 (26650.36 to 145278.21)	2792.11 (1137.02 to 6282.29)	0.41 (0.36 to 0.47)
Kiribati	92.87 (41.72 to 211.87)	2606.13 (1152.94 to 5924.62)	180.75 (80.51 to 413.99)	2814.94 (1230.83 to 6441.85)	0.34 (-0.68 to 1.37)
Kuwait	1869.94 (885.12 to 4004.2)	2816.15 (1333.93 to 5974.35)	8826.26 (4149.35 to 18828.96)	2665.62 (1263 to 5672.1)	-0.32 (-0.49 to -0.16)
Kyrgyzstan	9857.85 (4579.37 to 21874.58)	2311.4 (1063.85 to 5129.67)	13138.78 (6172.05 to 28801.94)	2245.82 (1055.68 to 4893.35)	-0.05 (-0.15 to 0.04)
Lao People's Democratic Republic	4882.86 (2322.15 to 10697.26)	2261.07 (1056.68 to 5006.72)	12052.62 (5507.78 to 27640.33)	2360.62 (1064.2 to 5439.25)	0.18 (0.04 to 0.32)

Latvia	13352.77 (6280.73 to 29760.87)	2280.76 (1063.2 to 5095.93)	18896.68 (8833.09 to 41548.95)	2279.58 (1065.19 to 5009.54)	0 (-0.09 to 0.08)
Lebanon	7836.48 (3757.89 to 16813.91)	2748.51 (1304.63 to 5911.68)	30296.25 (14590.09 to 64751.41)	2678.08 (1292.63 to 5719.41)	-0.08 (-0.17 to 0.01)
Lesotho	2638.62 (1175.62 to 5810.15)	2401 (1054.52 to 5314.02)	2762.44 (1197.25 to 6593.96)	2501.48 (1080.72 to 5891.8)	0.23 (0.05 to 0.42)
Liberia	2439.51 (1065.11 to 5791.1)	1772.1 (750.06 to 4228.73)	3878.45 (1643.56 to 9424.62)	1781.03 (744.21 to 4319.63)	0.05 (-0.13 to 0.24)
Libya	8641.68 (3876.84 to 18598.1)	3153.31 (1420.01 to 6754.3)	18469.15 (8309.67 to 41142.37)	2923.65 (1313.47 to 6500.08)	-0.27 (-0.36 to -0.19)
Lithuania	17129.35 (7940.5 to 38116.55)	2260.24 (1042.14 to 5041.8)	27476.49 (12698.79 to 60454.14)	2286.93 (1055.59 to 5040.92)	-0.01 (-0.08 to 0.07)
Luxembourg	1781.37 (845.43 to 3903.14)	1918.25 (896.05 to 4232.55)	3749.33 (1704.86 to 8165.38)	1794.37 (823.42 to 3906.35)	-0.2 (-0.42 to 0.03)
Madagascar	11624.37 (5427.14 to 26036.69)	2078.89 (955 to 4704.07)	20405.5 (9365.22 to 46540.36)	2121.92 (960.03 to 4809.42)	0.07 (-0.02 to 0.15)
Malawi	9720.84 (4316.77 to 22542.14)	2454.51 (1062.93 to 5702.04)	19963.52 (8594.45 to 46752.96)	2696.1 (1136.04 to 6338.13)	0.32 (0.23 to 0.41)
Malaysia	34252.39 (15574.7 to 76340.13)	2586.15 (1176.83 to 5741.96)	95956.79 (42350.04 to 220691.31)	2582.38 (1125.14 to 5939.85)	-0.06 (-0.1 to -0.02)

Maldives	197.48<br>(93.24 to 445.25)	2124.11<br>(993.46 to 4793.9)	958.48<br>(449.49 to 2096.74)	2227.07<br>(1045.27 to 4875.9)	0.08<br>(-0.54 to 0.7)
Mali	8325.15<br>(3467.35 to 19567.04)	2114.69<br>(852.64 to 4995.4)	18690.46<br>(7564.71 to 45416.16)	2109.09<br>(835.2 to 5104.27)	0.04<br>(-0.06 to 0.13)
Malta	1763.51<br>(817.56 to 3899.24)	2616.01<br>(1199.61 to 5782.11)	4874.32<br>(2252.58 to 10314.74)	2460.7<br>(1137.28 to 5228.71)	-0.21<br>(-0.42 to 0)
Marshall Islands	42.01<br>(18.45 to 97.79)	2624.67<br>(1115.74 to 6164.69)	67.18<br>(29.23 to 158.16)	2441.89<br>(1034.91 to 5769.74)	0.03<br>(-2.05 to 2.14)
Mauritania	2348.69<br>(1033.03 to 5507.07)	1921.18<br>(823.1 to 4522.42)	5104.17<br>(2167.16 to 12389.61)	1906.99<br>(799.66 to 4642.83)	-0.05<br>(-0.22 to 0.13)
Mauritius	2206.85<br>(1018.58 to 4944.5)	2427.14<br>(1100.36 to 5447.01)	6380.09<br>(2975.97 to 14173.4)	2260.46<br>(1052.76 to 5019.43)	-0.13<br>(-0.3 to 0.03)
Mexico	103817.76<br>(50079.93 to 228096.83)	1768.65<br>(844.79 to 3904.21)	321208.72<br>(153014.15 to 710577.77)	1716.4<br>(814.98 to 3798.48)	-0.09<br>(-0.12 to -0.07)
Micronesia (Federated States of)	153.21<br>(68.44 to 358.22)	2763.65<br>(1212.69 to 6464.03)	190.74<br>(84.16 to 446.48)	2720.69<br>(1197.51 to 6348.16)	-0.01<br>(-0.83 to 0.82)
Monaco	405.74<br>(184.35 to 886.53)	2640.5<br>(1197.96 to 5787.49)	574.3<br>(258.36 to 1219.9)	2630.63<br>(1196.75 to 5602.57)	-0.06<br>(-0.65 to 0.53)
Mongolia	3093.5<br>(1416.43 to 6956.92)	2439.7<br>(1091.63 to 5515.13)	5449.72<br>(2562.35 to 12158.81)	2338.31<br>(1087.79 to 5198.33)	-0.14<br>(-0.31 to 0.02)

Montenegro	2228.43<br>(1039.58 to 4871.91)	2267.32<br>(1057.66 to 4948.65)	3201.99<br>(1501.47 to 7063.97)	2228.03<br>(1024.67 to 4956.14)	-0.15<br>(-0.34 to 0.04)
Morocco	57068.04<br>(26723.53 to 127527.36)	2879.95<br>(1335.99 to 6446.25)	124502.25<br>(56217.05 to 280323.64)	2789.23<br>(1246.78 to 6275.82)	-0.09<br>(-0.12 to -0.05)
Mozambique	16014.79<br>(7041.37 to 36922.02)	2635.7<br>(1128.93 to 6115.22)	29323.34<br>(12149.53 to 71238.54)	2853.29<br>(1158.05 to 6947.89)	0.35<br>(0.28 to 0.42)
Myanmar	59304.42<br>(28676.2 to 132613.25)	2271.94<br>(1081.04 to 5092.59)	144748.69<br>(65609.32 to 330502.53)	2360.57<br>(1058.68 to 5401.23)	0.09<br>(0.05 to 0.13)
Namibia	1625.15<br>(736.12 to 3699.98)	2351.02<br>(1041.88 to 5351.36)	3723.45<br>(1621.38 to 8836.49)	2512.87<br>(1080.03 to 5980.21)	0.24<br>(0.01 to 0.46)
Nauru	10.23<br>(4.52 to 24.67)	2600.09<br>(1122.99 to 6222.12)	13.66<br>(6.02 to 31.73)	2602.66<br>(1121.63 to 6103.7)	NA
Nepal	16745.14<br>(7946.43 to 37013.07)	1628.37<br>(766.59 to 3625.19)	51622<br>(22495.23 to 119863.63)	1803.26<br>(772.27 to 4202.09)	0.34<br>(0.28 to 0.41)
Netherlands	101853.78<br>(47177.38 to 217540.28)	2806.3<br>(1291.06 to 6010)	191067.71<br>(86597.58 to 409654.18)	2773.21<br>(1258.85 to 5953.4)	0.08<br>(0 to 0.15)
New Zealand	18211.33<br>(8453.34 to 40119.78)	2708.09<br>(1242.57 to 5966.06)	40882.86<br>(18712.42 to 88134.16)	2563.4<br>(1176.46 to 5529.07)	-0.22<br>(-0.29 to -0.15)
Nicaragua	4798.42<br>(2321.77 to 10256.1)	2117.35<br>(1023.21 to 4508.34)	14262.82<br>(6959.19 to 29687.47)	2069.33<br>(1003.39 to 4317.99)	-0.02<br>(-0.13 to 0.09)

Niger	5123.98 (2233.07 to 11781.19)	1898.5 (803.76 to 4390.64)	15597 (6660.18 to 37074.26)	1919.85 (795.23 to 4570.99)	0.09 (-0.03 to 0.2)
Nigeria	89982.58 (39041.09 to 212874.48)	1718.97 (725.06 to 4073.44)	176748.53 (73500.57 to 431387.42)	1791.36 (730.26 to 4355.34)	0.28 (0.25 to 0.31)
Niue	11.15 (4.71 to 26.03)	2771.36 (1167.92 to 6473.67)	8.32 (3.66 to 19.17)	2519.91 (1099.1 to 5802.1)	-0.23 (-3.76 to 3.44)
North Macedonia	5565.88 (2605.82 to 12427.7)	2178.26 (1019.03 to 4857.47)	8944.24 (4273.5 to 19797.94)	2169.51 (1004.87 to 4889.12)	-0.03 (-0.17 to 0.1)
Northern Mariana Islands	32.1 (14.48 to 73.65)	2431.92 (1075.31 to 5564.24)	125.58 (56.79 to 287.69)	2385.18 (1064.18 to 5439.91)	0.18 (-1.81 to 2.21)
Norway	37877.68 (17580.63 to 82719.24)	2770.97 (1282.99 to 6059.99)	51266.56 (23106.96 to 111591.33)	2492.02 (1134.58 to 5426.43)	-0.41 (-0.46 to -0.35)
Oman	2222.83 (1010.53 to 4937.2)	2971.26 (1339.97 to 6539.22)	4925.72 (2277.9 to 11392.17)	2749.4 (1258.87 to 6293.47)	-0.27 (-0.45 to -0.09)
Pakistan	133673.52 (58151.92 to 313737.59)	1827.21 (782.26 to 4308.39)	254122.57 (105141.93 to 598842.99)	1936.7 (787.85 to 4555.51)	0.11 (0.09 to 0.13)
Palau	28.51 (12.62 to 65.99)	2509.32 (1077.36 to 5849.97)	50.88 (22.55 to 118.7)	2259.73 (981.05 to 5356.45)	0.21 (-2.03 to 2.49)
Palestine	3186.73 (1475.63 to 7230.24)	2889.54 (1316.32 to 6589.67)	7218.34 (3412.9 to 16331.26)	2687.98 (1248.87 to 6066.09)	-0.29 (-0.44 to -0.14)

Panama	4892.22 (2361.4 to 10550.56)	2029.69 (978.41 to 4374.32)	15875.85 (7559.2 to 32999.05)	2021.95 (967.9 to 4203.85)	-0.02 (-0.13 to 0.09)
Papua New Guinea	3891.18 (1791.46 to 8687.76)	2392.93 (1094.38 to 5321.45)	10393.06 (4699.84 to 24059.46)	2278.54 (1021.35 to 5289.84)	-0.23 (-0.38 to -0.07)
Paraguay	9559.7 (4372.64 to 21578.75)	2712.63 (1240.46 to 6116.67)	24006.57 (10640.77 to 52575.89)	2706.33 (1204.5 to 5914.01)	-0.04 (-0.12 to 0.04)
Peru	29597.71 (13461.83 to 66668.51)	1590.8 (724.02 to 3577.05)	87661.37 (40292.22 to 190164.22)	1550.9 (717 to 3360.14)	-0.15 (-0.2 to -0.11)
Philippines	80238.64 (38375.04 to 176513.59)	2272.06 (1065.79 to 5036.96)	235978.54 (108231.71 to 524684.48)	2375.2 (1079.47 to 5262.16)	0.14 (0.1 to 0.17)
Poland	157521.31 (73684.08 to 352311.91)	2356.06 (1091.81 to 5269.65)	308882.26 (142676.19 to 681485.37)	2289.8 (1060.25 to 5056.85)	-0.08 (-0.1 to -0.06)
Portugal	56234.97 (25820.61 to 126392.9)	2591.43 (1162.55 to 5861.19)	137959.87 (62898.34 to 295742.14)	2522.7 (1159.11 to 5415.17)	-0.04 (-0.08 to 0)
Puerto Rico	11844.88 (5660.28 to 25982.93)	1922.82 (906.51 to 4244.85)	29993.51 (14346.39 to 62663.39)	1846.03 (886.91 to 3879.48)	-0.16 (-0.24 to -0.08)
Qatar	219.22 (97.57 to 504.72)	2996.01 (1308.8 to 6929.82)	1510.68 (686.5 to 3430.78)	2726.85 (1214.41 to 6211.68)	-0.39 (-0.94 to 0.17)
Republic of Korea	105728.79 (47389.8 to 238811.54)	3299.54 (1430.01 to 7448.81)	454889.65 (209429.4 to 943000.2)	2911.27 (1332.63 to 6033.94)	-0.24 (-0.27 to -0.2)

Republic of Moldova	12374.16<br>(5825.02 to 27550.18)	2245.27<br>(1033.19 to 5045.95)	22724.72<br>(10773.66 to 48862.22)	2161.35<br>(1028.54 to 4650.69)	-0.13<br>(-0.21 to -0.04)
Romania	84690.74<br>(40269.76 to 190623.83)	2227.6<br>(1036.27 to 5055.66)	158153.44<br>(73723.17 to 348683.56)	2221.77<br>(1033.83 to 4909.41)	-0.01<br>(-0.05 to 0.02)
Russian Federation	603508.94<br>(284545.34 to 1354574.22)	2358.92<br>(1095.69 to 5310.59)	958024.27<br>(446207.68 to 2144315.13)	2308.31<br>(1076.63 to 5160.68)	-0.06<br>(-0.08 to -0.05)
Rwanda	6898.77<br>(3066.62 to 16201.13)	2556.13<br>(1106.71 to 6016.42)	18188.16<br>(7587.19 to 43798.16)	2865.74<br>(1174.01 to 6883.02)	0.4<br>(0.29 to 0.51)
Saint Kitts and Nevis	102.74<br>(49.85 to 223.94)	1764.98<br>(829.33 to 3890.53)	148.57<br>(69.64 to 331.27)	1783.59<br>(840.23 to 3947.07)	0.03<br>(-0.93 to 1)
Saint Lucia	240.03<br>(114.68 to 528.23)	1899.61<br>(883.95 to 4252.53)	710.69<br>(336.42 to 1560.34)	1845.14<br>(869.89 to 4059.26)	-0.12<br>(-0.69 to 0.46)
Saint Vincent and the Grenadines	213.56<br>(101.57 to 475.95)	1963.85<br>(913.63 to 4407.74)	389.57<br>(187.44 to 852.87)	1817.22<br>(864.35 to 4008.97)	-0.21<br>(-0.83 to 0.42)
Samoa	281.2<br>(122.07 to 658.91)	2774.64<br>(1194.33 to 6454.46)	478.5<br>(204.33 to 1126.01)	2653.75<br>(1120.91 to 6221.94)	-0.14<br>(-0.67 to 0.39)
San Marino	177.47<br>(83.27 to 373.02)	2480.62<br>(1163.78 to 5217.42)	407.38<br>(190.16 to 838.35)	2240.81<br>(1063.07 to 4616.75)	-0.3<br>(-1.05 to 0.44)
Sao Tome and Principe	157.16<br>(68.3 to 371.68)	1800.89<br>(772.96 to 4262.6)	222.88<br>(95.66 to 527.57)	1770.14<br>(746.66 to 4182.45)	-0.01<br>(-0.73 to 0.71)

Saudi Arabia	19293.75 (8658.08 to 43781.17)	2935.91 (1300.67 to 6664.29)	37710.65 (16831.43 to 86668.88)	2657.99 (1167.36 to 6102.58)	-0.36 (-0.42 to -0.3)
Senegal	7475.07 (3215.93 to 17514.34)	1929.71 (810.76 to 4525)	18498.7 (7579.94 to 45019.2)	2025.65 (817.56 to 4931.15)	0.19 (0.1 to 0.29)
Serbia	30585.57 (14095.6 to 68426.83)	2237.95 (1020.85 to 5051.94)	65545.67 (30430.4 to 145183.48)	2198.59 (1019.05 to 4872.77)	-0.09 (-0.14 to -0.04)
Seychelles	230.7 (105.75 to 513.95)	2419 (1104.3 to 5390.44)	359.82 (163.7 to 794.92)	2264.8 (1024.05 to 5005.84)	-0.21 (-0.79 to 0.38)
Sierra Leone	4533.55 (2053.17 to 10309.02)	1751.12 (772.31 to 4022.49)	7432.01 (3244.02 to 17618.96)	1756.79 (752.91 to 4187.58)	0.04 (-0.1 to 0.17)
Singapore	5546.92 (2596.18 to 12337.76)	1929.91 (887.96 to 4293.05)	26381.62 (12654.8 to 55120.26)	1904.92 (914.82 to 3974.87)	-0.02 (-0.12 to 0.08)
Slovakia	21339.17 (9885.34 to 48024.79)	2259.74 (1038.21 to 5093.81)	36392.3 (16849.96 to 79300.09)	2244.59 (1035.77 to 4893.56)	-0.02 (-0.09 to 0.04)
Slovenia	9356.52 (4297.7 to 20797.61)	2238.49 (1025.17 to 4973.12)	20770.37 (9626.28 to 44387.55)	2240.99 (1047.13 to 4785.26)	0.01 (-0.09 to 0.1)
Solomon Islands	312.68 (138.98 to 745.16)	2505.3 (1099.33 to 5987.09)	824.22 (366.02 to 1917.3)	2499.97 (1092.72 to 5853.83)	0.06 (-0.54 to 0.66)
Somalia	4731.75 (2108.8 to 11028.52)	2418.78 (1060.78 to 5633.58)	12203.2 (5298.89 to 28670.8)	2586.06 (1107.46 to 6170.68)	0.33 (0.19 to 0.47)

South Africa	67637.79<br>(31091.97 to 151367.28)	2321.05<br>(1056.8 to 5206.31)	135783.66<br>(61053.91 to 313429.58)	2357.71<br>(1042.29 to 5431.99)	0.03<br>(-0.01 to 0.06)
South Sudan	7010.94<br>(3096.88 to 16357.39)	2379.3<br>(1026.73 to 5560.59)	9071.21<br>(3820.94 to 21033.54)	2398.2<br>(996.91 to 5571.93)	0.07<br>(-0.04 to 0.18)
Spain	256199.42<br>(116074.57 to 566270.64)	2639.85<br>(1183.09 to 5847.34)	543185.81<br>(242318.26 to 1152551.84)	2436.68<br>(1100.09 to 5184.36)	-0.22<br>(-0.25 to -0.2)
Sri Lanka	29057.91<br>(13929.6 to 64612.25)	2169.06<br>(1018.35 to 4853.96)	82743.65<br>(38427.93 to 179540.76)	2134.72<br>(982.12 to 4641.42)	-0.06<br>(-0.1 to -0.01)
Sudan	31659.34<br>(14997.4 to 73323.84)	2766.88<br>(1283.89 to 6402.52)	57939.63<br>(27093.85 to 128000.57)	2570.08<br>(1193.82 to 5658.23)	-0.27<br>(-0.32 to -0.22)
Suriname	748.24<br>(356.79 to 1644.06)	1948.59<br>(934.63 to 4269.39)	1800.6<br>(864.82 to 3851.53)	1874.96<br>(901.14 to 4005.04)	-0.12<br>(-0.41 to 0.18)
Sweden	78513.22<br>(37671.28 to 166647.62)	2574.51<br>(1228.48 to 5484.16)	114512.46<br>(54495.73 to 236715.8)	2428.88<br>(1162.03 to 5023.61)	-0.05<br>(-0.11 to 0.01)
Switzerland	54092.54<br>(25009.44 to 116427.53)	2608.97<br>(1203.1 to 5627.57)	99017.11<br>(45207.91 to 207263.74)	2481.61<br>(1146.9 to 5198.01)	-0.27<br>(-0.31 to -0.23)
Syrian Arab Republic	17876.86<br>(8353.41 to 40444)	2804.29<br>(1302.94 to 6345.24)	39205.33<br>(18379.03 to 88602.68)	2627.91<br>(1204.52 to 6011.55)	-0.22<br>(-0.28 to -0.15)
Taiwan (Province of China)	38634.1<br>(17505.65 to 87844.74)	2014.02<br>(893.61 to 4557.22)	167347.02<br>(76095.64 to 359173.63)	2163.46<br>(990.38 to 4643.15)	0.07<br>(0 to 0.13)

Tajikistan	8898.68<br>(4075.92 to 19622.66)	2297.76<br>(1046.84 to 5071.26)	13906.91<br>(6234.52 to 31889.4)	2272.54<br>(1015.03 to 5170.99)	-0.02<br>(-0.12 to 0.07)
Thailand	109636.08<br>(48295.36 to 248886.36)	2382.79<br>(1041.1 to 5393.93)	427148.7<br>(194452.13 to 927574.48)	2290.68<br>(1047.41 to 4961.51)	-0.16<br>(-0.19 to -0.13)
Timor-Leste	616.79<br>(293.31 to 1355.7)	2214.35<br>(1047.56 to 4864.77)	2420.32<br>(1120.38 to 5535.15)	2315.28<br>(1046.3 to 5344.91)	0.21<br>(-0.1 to 0.52)
Togo	2472.38<br>(1064.41 to 5809.79)	1882.22<br>(790.83 to 4424.16)	6962.39<br>(2949.37 to 16674.02)	1977.21<br>(818.28 to 4755.81)	0.17<br>(0 to 0.33)
Tokelau	5.52<br>(2.33 to 13.42)	2841.87<br>(1186.79 to 6894.5)	6.41<br>(2.8 to 14.73)	2589.52<br>(1124.39 to 5957.28)	NA
Tonga	190.24<br>(84.53 to 436.88)	2681.28<br>(1181.47 to 6125.8)	322.72<br>(142.73 to 717.94)	2622.26<br>(1154.35 to 5835.05)	-0.08<br>(-0.7 to 0.55)
Trinidad and Tobago	2281.78<br>(1105.6 to 5011.44)	1874.85<br>(885.96 to 4157.43)	5653.24<br>(2701.97 to 12355.37)	1824.59<br>(873.94 to 3973.89)	-0.03<br>(-0.22 to 0.16)
Tunisia	19328.33<br>(9042.85 to 42756.33)	3058.97<br>(1395.19 to 6784.91)	53736.93<br>(25060.32 to 118239.62)	2827.34<br>(1309.72 to 6206.61)	-0.28<br>(-0.34 to -0.22)
Turkey	144439.18<br>(66331.12 to 316276.8)	3161.01<br>(1447.23 to 6887.97)	394366.23<br>(184988.14 to 874606.83)	2864.64<br>(1337.61 to 6354.68)	-0.4<br>(-0.47 to -0.33)
Turkmenistan	5455.84<br>(2535.32 to 12236.48)	2288.5<br>(1055.1 to 5126.13)	11002.22<br>(5084.42 to 24501.19)	2166.25<br>(1011.17 to 4805.84)	-0.26<br>(-0.37 to -0.14)

Tuvalu	18.8<br>(8.3 to 44.3)	2779.64<br>(1203.22 to 6618.5)	32.94<br>(14.66 to 77.58)	2650.23<br>(1156.3 to 6254.74)	NA
Uganda	16570.86<br>(7469.65 to 36974.37)	2315.77<br>(1014.13 to 5208.79)	40592.56<br>(17201.27 to 96588.49)	2638.65<br>(1102.22 to 6242.24)	0.47<br>(0.4 to 0.54)
Ukraine	246709.06<br>(116026.01 to 554219.87)	2342.26<br>(1084.38 to 5284.77)	313360.09<br>(143149.61 to 696183.11)	2299.23<br>(1045.62 to 5124.06)	-0.03<br>(-0.05 to -0.01)
United Arab Emirates	1020.46<br>(454.32 to 2327.41)	2884.53<br>(1283.06 to 6411.43)	4702.81<br>(2124.9 to 10623.69)	2486.63<br>(1101.31 to 5585.47)	-0.45<br>(-0.74 to -0.16)
United Kingdom	398572.91<br>(183252.51 to 885211.18)	2382.23<br>(1085.45 to 5302.88)	610383.99<br>(278517.94 to 1327082.42)	2276.66<br>(1041.96 to 4961.21)	-0.05<br>(-0.11 to 0.01)
United Republic of Tanzania	31198.83<br>(13750.52 to 72048.83)	2589.03<br>(1112.12 to 5913.67)	74352.53<br>(31414.45 to 177154.9)	2560.83<br>(1068.6 to 6050.74)	-0.05<br>(-0.1 to 0)
United States of America	1856300.9<br>(850857.39 to 4061791.01)	3070.63<br>(1403.81 to 6724.76)	3278708.72<br>(1481016.26 to 7109464.34)	2957.48<br>(1343.8 to 6417.71)	-0.13<br>(-0.15 to -0.12)
United States Virgin Islands	208.39<br>(98.88 to 464.73)	1941.35<br>(903.55 to 4343.87)	545.62<br>(265.63 to 1157.4)	1753.96<br>(836.39 to 3767.29)	-0.4<br>(-1 to 0.2)
Uruguay	15013.91<br>(7044.85 to 33210.05)	2216.48<br>(1031.41 to 4910.78)	25843.27<br>(11712.8 to 56441.65)	2205.65<br>(1009.39 to 4817.96)	-0.02<br>(-0.1 to 0.06)
Uzbekistan	38730.38<br>(18013.41 to 86053.14)	2188.8<br>(1013.04 to 4877.74)	63517.62<br>(29347.64 to 143128.93)	2128.62<br>(981.79 to 4776.47)	-0.07<br>(-0.12 to -0.03)

Vanuatu	142.95<br>(63.07 to 332.64)	2430.86<br>(1048.84 to 5700.12)	397.19<br>(175.62 to 942.44)	2405.1<br>(1047.1 to 5739.17)	0.03<br>(-0.82 to 0.89)
Venezuela (Bolivarian Republic of)	34322.56<br>(16607.59 to 73772.87)	2402.65<br>(1157.79 to 5153.13)	110617.71<br>(52518.12 to 234797.73)	2373.49<br>(1132 to 5021.11)	-0.08<br>(-0.12 to -0.04)
Viet Nam	154685.98<br>(69445.45 to 343488.2)	2662.75<br>(1189.6 to 5908.07)	361830.71<br>(156700.28 to 843956.27)	2764.14<br>(1193.35 to 6412.21)	0.09<br>(0.07 to 0.11)
Yemen	15463.19<br>(7169.81 to 35704.26)	3014.16<br>(1382.3 to 6873.28)	42302.4<br>(19573.06 to 97404.57)	2829.35<br>(1290.62 to 6550.83)	-0.23<br>(-0.3 to -0.16)
Zambia	6909.46<br>(3096.38 to 15623.48)	2307.34<br>(1010.63 to 5261.91)	16586.39<br>(7223.5 to 38540.75)	2504.81<br>(1072.32 to 5808.08)	0.34<br>(0.23 to 0.45)
Zimbabwe	11429.62<br>(5006.11 to 25762.18)	2511.42<br>(1075.04 to 5704.71)	15564.22<br>(6774.02 to 36442.02)	2419.09<br>(1040.68 to 5666.99)	-0.07<br>(-0.16 to 0.01)

DALYs, disability-adjusted life-years; ASR, age-standardized rate; 95% UI, 95% Uncertainty Intervals; 95% CI, 95% Confidence Interval.

S9 Table Statistics and projections of ADOD cases and ASIR in the population aged 55 years and older (1992-2046).

Prediction model	Timeperiod	Number of cases			ASIR		
		Both	Male	Female	Both	Male	Female
Nordpred model prediction	1992	3856614	1336054	2520560	667.22	571.41	730.59
	1993	3971003	1376490	2594513	668.92	572.16	733.16
	1994	4083983	1416604	2667380	670.12	572.66	735.07
	1995	4196136	1457513	2738622	670.71	572.91	736.14
	1996	4305695	1497751	2807944	670.8	572.74	736.67
	1997	4417716	1538345	2879371	670.76	572.29	737.23
	1998	4533420	1579687	2953733	670.59	571.72	737.64
	1999	4655168	1623156	3032013	670.31	571.11	737.89
	2000	4786224	1671252	3114971	669.88	570.65	737.8
	2001	4925204	1723768	3201436	669.47	570.56	737.47
	2002	5071935	1780835	3291100	669.21	570.79	737.23
	2003	5221844	1839240	3382604	669.04	571.16	737
	2004	5381028	1900680	3480348	668.93	571.58	736.8
	2005	5545173	1963518	3581655	668.46	571.66	736.27
	2006	5719548	2031461	3688086	667.37	571.27	735.06
	2007	5900235	2103341	3796894	665.9	570.72	733.34
	2008	6082827	2176400	3906427	664.28	570.09	731.41
	2009	6273771	2252699	4021072	662.78	569.5	729.63

2010	6473139	2331017	4142122	661.54	569.09	728.19
2011	6686143	2414276	4271867	660.82	569.02	727.38
2012	6914173	2504776	4409396	660.75	569.58	727.37
2013	7147681	2597266	4550416	661.2	570.54	727.94
2014	7386031	2690365	4695666	662.01	571.75	728.89
2015	7622984	2781428	4841556	662.5	572.54	729.53
2016	7877127	2877188	4999938	662.99	572.73	730.52
2017	8150728	2979336	5171391	663.9	572.86	732.23
2018	8429236	3084131	5345105	664.66	572.86	733.77
2019	8715434	3193437	5521998	664.88	572.71	734.55
2020	8980609	3297067	5683542	665.67	574.16	735.08
2021	9405626	3447541	5958085	679.44	585.06	751.18
2022	9723582	3586751	6136831	671.29	577.74	743.02
2023	10055036	3711760	6343276	672.51	578.43	744.83
2024	10422480	3851180	6571300	673.71	579.11	746.64
2025	10808018	3997642	6810376	674.78	579.78	748.18
2026	11202611	4146236	7056375	675.88	580.45	749.72
2027	11596918	4292808	7304109	677.01	581.12	751.26
2028	12011102	4446131	7564970	678.14	581.79	752.8
2029	12460146	4612621	7847525	679.26	582.46	754.35

	2030	12921950	4784682	8137268	680.01	583	755.31
	2031	13391993	4958577	8433417	680.77	583.54	756.28
	2032	13859337	5129871	8729466	681.55	584.08	757.25
	2033	14339685	5305402	9034283	682.32	584.62	758.22
	2034	14847383	5491275	9356109	683.09	585.16	759.18
	2035	15359833	5680500	9679333	683.38	585.49	759.5
	2036	15876339	5870881	10005458	683.68	585.83	759.83
	2037	16387227	6058759	10328468	683.98	586.16	760.15
	2038	16908448	6250978	10657470	684.28	586.49	760.47
	2039	17453468	6453115	11000353	684.56	586.83	760.79
	2040	18004317	6658716	11345601	684.62	587.03	760.87
	2041	18554754	6864683	11690071	684.68	587.22	760.96
	2042	19094170	7066896	12027274	684.75	587.42	761.04
	2043	19639081	7272279	12366802	684.81	587.62	761.12
	2044	20202976	7486280	12716696	684.86	587.82	761.21
	2045	20772432	7703254	13069177	684.91	588.02	761.29
	2046	21334937	7918006	13416931	684.96	588.22	761.37
BAPC model prediction	1992	3856602	1336047	2520555	667.22	571.4	730.58
	1993	3970980	1376483	2594497	668.94	572.21	733.16
	1994	4083952	1416598	2667354	670.16	572.74	735.08

1995	4196121	1457503	2738618	670.74	573	736.16
1996	4305723	1497754	2807969	670.83	572.81	736.7
1997	4417746	1538354	2879392	670.78	572.32	737.25
1998	4533426	1579684	2953742	670.59	571.72	737.65
1999	4655164	1623162	3032002	670.31	571.09	737.89
2000	4786242	1671276	3114966	669.88	570.63	737.8
2001	4925244	1723791	3201453	669.47	570.55	737.48
2002	5071973	1780850	3291123	669.22	570.79	737.24
2003	5221883	1839252	3382631	669.05	571.17	737.02
2004	5380985	1900650	3480334	668.93	571.59	736.8
2005	5545116	1963493	3581623	668.46	571.67	736.27
2006	5719529	2031462	3688068	667.37	571.27	735.05
2007	5900243	2103350	3796893	665.9	570.72	733.34
2008	6082852	2176406	3906446	664.28	570.08	731.41
2009	6273782	2252699	4021083	662.77	569.49	729.63
2010	6473170	2331028	4142141	661.53	569.07	728.19
2011	6686228	2414319	4271909	660.81	569.02	727.39
2012	6914238	2504813	4409425	660.76	569.59	727.37
2013	7147712	2597289	4550423	661.21	570.55	727.94
2014	7385966	2690335	4695631	662.01	571.75	728.88

2015	7622864	2781347	4841517	662.5	572.52	729.52
2016	7877048	2877129	4999918	662.98	572.7	730.51
2017	8150599	2979274	5171325	663.89	572.83	732.22
2018	8429114	3084097	5345016	664.65	572.85	733.76
2019	8715709	3193613	5522095	664.89	572.73	734.56
2020	8981715	3297546	5684170	665.75	574.24	735.16
2021	9404699	3447107	5957592	679.39	585.02	751.11
2022	9899529	3650339	6249190	683.38	587.43	756.57
2023	10338216	3815355	6522861	691.42	593.95	765.84
2024	10822764	3998697	6824067	699.58	600.59	775.23
2025	11339682	4194017	7145665	707.94	607.39	784.78
2026	11878895	4396619	7482276	716.62	614.43	794.58
2027	12427021	4601072	7825949	725.41	621.54	804.33
2028	13007806	4817184	8190622	734.39	628.78	814.17
2029	13639535	5052647	8586888	743.58	636.16	824.15
2030	14309316	5301960	9007356	753.04	643.76	834.28
2031	15005431	5560425	9445006	762.83	651.6	844.56
2032	15709956	5821520	9888436	772.65	659.49	854.61
2033	16443995	6093285	10350710	782.66	667.46	864.66
2034	17227703	6383887	10843816	792.95	675.58	874.85

2035	18051399	6689735	11361664	803.61	683.94	885.23
2036	18903550	7006971	11896579	814.64	692.58	895.72
2037	19765375	7329321	12436054	825.74	701.25	905.91
2038	20660620	7665598	12995022	837.08	710.01	916.09
2039	21611759	8024495	13587263	848.84	718.95	926.45
2040	22609895	8402787	14207107	861.15	728.2	937.09
2041	23641753	8796151	14845602	874.02	737.79	947.93
2042	24686080	9197201	15488879	887.17	747.49	958.63
2043	25769387	9615912	16153475	900.79	757.37	969.4
2044	26916920	10061821	16855098	915.06	767.54	980.46
2045	28118316	10530552	17587764	930.14	778.09	991.9
2046	29358369	11016524	18341845	946.02	789.03	1003.67

ASIR, age-standardized incidence rate; ADOD, Alzheimer's disease and other dementias; BAPC, Bayesian age-period-cohort.

S10 Table Statistics and projections of ADOD cases and ASMR in the population aged 55 years and older (1992-2046).

Prediction model	Timeperiod	Number of cases			ASMR		
		Both	Male	Female	Both	Male	Female
Nordpred model prediction	1992	707375	212663	494712	147.57	118.68	162.8
	1993	732534	220152	512382	147.8	118.72	163.16
	1994	757246	227449	529797	147.89	118.65	163.38
	1995	782658	235069	547589	148.13	118.67	163.8
	1996	807115	242549	564566	148.19	118.67	163.96
	1997	830874	250090	580785	148.04	118.62	163.83
	1998	856430	257949	598482	148.02	118.64	163.85
	1999	883615	266010	617605	148	118.5	163.96
	2000	911960	275185	636774	147.79	118.45	163.73
	2001	941040	284548	656492	147.61	118.38	163.56
	2002	971939	294732	677207	147.67	118.5	163.66
	2003	1003496	305100	698396	147.77	118.72	163.76
	2004	1034809	315304	719505	147.43	118.6	163.36
	2005	1072199	327290	744908	147.45	118.62	163.42
	2006	1109488	339930	769559	147.06	118.49	162.98
	2007	1150985	354173	796812	146.93	118.56	162.85
	2008	1195209	368924	826286	146.96	118.63	162.97
	2009	1240972	384538	856434	146.73	118.65	162.73

2010	1291992	401556	890436	146.71	118.83	162.7
2011	1344173	418883	925290	146.65	118.93	162.65
2012	1397045	437361	959684	146.48	119.07	162.44
2013	1452867	457174	995692	146.64	119.6	162.5
2014	1510876	476624	1034252	147.03	120.14	162.9
2015	1570264	496855	1073409	147.3	120.65	163.09
2016	1631793	516748	1115045	147.41	120.71	163.34
2017	1694126	538284	1155842	147.42	120.82	163.36
2018	1756567	560001	1196567	147.3	120.82	163.27
2019	1819952	583124	1236828	147.06	120.92	162.92
2020	1874988	601258	1273730	146.75	120.73	162.62
2021	1943723	622676	1321047	148.24	121.94	164.29
2022	2032700	665958	1366742	147.34	121.63	163.63
2023	2100662	691048	1409614	147.41	121.82	163.74
2024	2182065	721106	1460959	147.46	122.01	163.85
2025	2267942	752443	1515498	147.49	122.13	163.97
2026	2353415	783494	1569920	147.54	122.26	164.09
2027	2435434	813095	1622339	147.61	122.38	164.21
2028	2524039	845107	1678932	147.68	122.5	164.33
2029	2625642	881890	1743752	147.74	122.62	164.45

	2030	2733430	920501	1812930	147.76	122.68	164.54
	2031	2843896	959683	1884213	147.78	122.74	164.63
	2032	2953658	998091	1955567	147.82	122.79	164.71
	2033	3070504	1038790	2031714	147.87	122.85	164.8
	2034	3200051	1083932	2116119	147.9	122.9	164.88
	2035	3335459	1130633	2204826	147.88	122.89	164.92
	2036	3473268	1177728	2295540	147.87	122.87	164.95
	2037	3609456	1223731	2385724	147.86	122.85	164.98
	2038	3750958	1271313	2479645	147.85	122.84	165.01
	2039	3903311	1322618	2580693	147.84	122.82	165.05
	2040	4060822	1375744	2685078	147.82	122.8	165.06
	2041	4219229	1429136	2790092	147.79	122.77	165.07
	2042	4374513	1481464	2893049	147.77	122.75	165.08
	2043	4534228	1535495	2998734	147.74	122.73	165.09
	2044	4703709	1593187	3110522	147.71	122.7	165.1
	2045	4877053	1652476	3224576	147.68	122.68	165.11
	2046	5049273	1711611	3337662	147.64	122.66	165.11
BAPC model prediction	1992	707476	212702	494774	147.6	118.72	162.83
	1993	732512	220131	512381	147.79	118.72	163.16
	1994	757271	227458	529813	147.89	118.65	163.38

1995	782615	235064	547552	148.11	118.64	163.77
1996	807050	242533	564517	148.15	118.61	163.93
1997	830920	250091	580829	148.02	118.55	163.83
1998	856451	257933	598518	147.99	118.55	163.85
1999	883573	266034	617539	147.97	118.44	163.94
2000	911990	275193	636797	147.78	118.41	163.74
2001	941186	284612	656575	147.63	118.38	163.58
2002	971952	294741	677211	147.67	118.49	163.66
2003	1003345	305031	698314	147.75	118.68	163.74
2004	1034875	315313	719562	147.45	118.61	163.38
2005	1072077	327248	744829	147.45	118.64	163.41
2006	1109594	339976	769617	147.08	118.53	163
2007	1151035	354178	796857	146.95	118.58	162.87
2008	1195180	368928	826252	146.96	118.65	162.96
2009	1241011	384555	856456	146.75	118.68	162.74
2010	1291951	401527	890424	146.72	118.86	162.7
2011	1344109	418877	925233	146.65	118.96	162.64
2012	1397149	437437	959711	146.5	119.11	162.44
2013	1452997	457206	995792	146.66	119.61	162.52
2014	1510926	476652	1034274	147.02	120.12	162.9

2015	1570130	496723	1073408	147.28	120.6	163.09
2016	1631725	516758	1114967	147.4	120.68	163.32
2017	1694044	538265	1155779	147.41	120.79	163.35
2018	1756467	559988	1196479	147.29	120.82	163.25
2019	1819979	583088	1236892	147.06	120.92	162.93
2020	1875713	601586	1274127	146.8	120.79	162.67
2021	1943235	622467	1320768	148.21	121.91	164.26
2022	2044526	668455	1376071	148.2	122.08	164.74
2023	2119506	695348	1424158	148.73	122.57	165.42
2024	2208866	727454	1481412	149.27	123.07	166.13
2025	2304225	761577	1542648	149.85	123.58	166.88
2026	2400379	795751	1604628	150.49	124.11	167.67
2027	2493651	828675	1664976	151.14	124.63	168.44
2028	2594491	864355	1730136	151.8	125.16	169.2
2029	2709913	905307	1804606	152.48	125.7	169.99
2030	2834243	949169	1885074	153.2	126.26	170.81
2031	2963114	994181	1968932	153.97	126.83	171.65
2032	3092257	1038801	2053456	154.75	127.39	172.46
2033	3230110	1086290	2143820	155.54	127.94	173.26
2034	3383161	1139033	2244127	156.36	128.51	174.06

2035	3546199	1194882	2351317	157.22	129.08	174.89
2036	3714402	1251980	2462422	158.12	129.66	175.74
2037	3882619	1308583	2574036	159.04	130.23	176.55
2038	4058690	1367610	2691080	159.97	130.79	177.35
2039	4249256	1431539	2817717	160.93	131.35	178.14
2040	4449216	1498575	2950640	161.93	131.91	178.95
2041	4653555	1567070	3086484	162.97	132.48	179.77
2042	4856969	1635438	3221531	164.03	133.03	180.55
2043	5068309	1706808	3361501	165.11	133.57	181.31
2044	5294386	1783571	3510815	166.23	134.11	182.07
2045	5529387	1863669	3665718	167.41	134.66	182.85
2046	5768084	1945295	3822789	168.65	135.21	183.64

ASMR, age-standardized mortality rate; ADOD, Alzheimer's disease and other dementias; BAPC, Bayesian age-period-cohort.

Supplementary Materials

Statistical analysis

(1) Due to the inherent linear relationship among age, period, and cohort (i.e., birth cohort = period − age), it is statistically impossible to independently estimate the effects of these three factors, a challenge referred to as the 'identifiability problem.' In this study, we utilized a specialized tool developed by the National Cancer Institute for Age-Period-Cohort (APC) analysis. This method effectively addresses the identifiability issue by generating estimable APC parameters and functions without imposing arbitrary constraints on the model. A detailed description of the methodology is provided in previously published literature [1]. In the Age-Period-Cohort (APC) model, the Period Relative Risk (RR) reflects the relative risk of Alzheimer's Disease and Other Dementias (ADOD) for each observation period, compared to the reference period, with adjustments made for age effects and nonlinear cohort effects. Similarly, the Cohort RR represents the relative risk for each birth cohort relative to the reference cohort, while controlling for age effects and nonlinear period effects. An RR value greater than 1 indicates an increased risk of ADOD incidence, prevalence, or mortality, whereas an RR value less than 1 suggests a reduction in the risk of ADOD incidence, prevalence, or mortality. The selection of the reference period (or cohort) is arbitrary and does not influence the interpretation of the results. For additional details, refer to the studies by Huang [2]and Su [3].

(2) Nordpred employs an enhanced Poisson age-period model with a power-link function, originally developed for cancer prediction but adapted for dementia in this study [4]. The BAPC model utilizes a Bayesian hierarchical framework, fitted using Integrated Nested Laplace Approximation (INLA), which assumes that adjacent age, period, and cohort groups exhibit similar effects [5]. The model's probabilistic predictions are well-calibrated, with prediction intervals exhibiting moderate width. While these methods are statistically robust, they rely on the assumption of consistent future trends, which may underestimate nonlinear or policy-driven changes.

References

1. Rosenberg PS, Check DP, Anderson WF. A web tool for age-period-cohort analysis of cancer incidence and mortality rates. Cancer Epidemiol Biomarkers Prev. 2014;23(11):2296–302.doi: 10.1158/1055-9965.Epi-14-0300.
2. Huang D, Lai H, Shi X, Jiang J, Zhu Z, Peng J, et al. Global temporal trends and projections of acute hepatitis E incidence among women of childbearing age: Age-period-cohort analysis 2021. J Infect. 2024;89(4):106250.doi: 10.1016/j.jinf.2024.106250.
3. Su Z, Zou Z, Hay SI, Liu Y, Li S, Chen H, et al. Global, regional, and national time trends in mortality for congenital heart disease, 1990-2019: An age-period-cohort analysis for the Global Burden of Disease 2019 study. EClinicalMedicine. 2022;43:101249.doi: 10.1016/j.eclinm.2021.101249.
4. Møller B, Fekjaer H, Hakulinen T, Sigvaldason H, Storm HH, Talbäck M, et al. Prediction of cancer incidence in the Nordic countries: empirical comparison of different approaches. Stat Med. 2003;22(17):2751–66.doi: 10.1002/sim.1481.
5. Riebler A, Held L. Projecting the future burden of cancer: Bayesian age-period-cohort analysis with integrated nested Laplace approximations. Biom J. 2017;59(3):531–49.doi: 10.1002/bimj.201500263.
